# Molecular analyses of pseudoscorpions in a subterranean biodiversity hotspot reveal cryptic diversity and microendemism

**DOI:** 10.1038/s41598-022-26298-5

**Published:** 2023-01-09

**Authors:** Dora Hlebec, Martina Podnar, Mladen Kučinić, Danilo Harms

**Affiliations:** 1 Department of Biology, Faculty of Science, University of Zagreb, Zagreb, Croatia; 2 Section of Arachnology, Department of Invertebrates, Museum of Nature Hamburg - Zoology, Leibniz Institute for the Analysis of Biodiversity Change, Hamburg, Germany; 3 Croatian Biospeleological Society, Zagreb, Croatia; 4 Croatian Natural History Museum, Zagreb, Croatia

**Keywords:** Ecology, Evolution, Molecular biology, Zoology

## Abstract

Nested within the Mediterranean biodiversity hotspot, the Dinaric Karst of the western Balkans is one of the world’s most heterogeneous subterranean ecosystems and renowned for its highly diverse and mostly endemic fauna. The evolutionary processes leading to both endemism and diversity remain insufficiently understood, and large-scale analyses on taxa that are abundant in both subterranean and surface habitats remain infrequent. Here, we provide the first comprehensive molecular study on Croatian pseudoscorpions, a lineage of arachnids that is common and diverse in both habitats. Phylogenetic reconstructions using 499 *COI* sequences derived from 128 morphospecies collected across the Dinaric Karst show that: (i) occurrence in karstic microhabitats boosters speciation and endemism in the most diverse genera *Chthonius* C.L. Koch, 1843 (37 morphospecies) and *Neobisium* Chamberlin, 1930 (34 morphospecies), (ii) evidence for ongoing diversification is found in many species and species complexes through low optimal thresholds (OTs) and species delineation analyses, and (iii) landscape features, such as mountain ranges, correlate with patterns of genetic diversity in the diverse genus *Neobisium*. We present two synonymies: *Protoneobisium* Ćurčić, 1988 = *Neobisium*, syn. nov., and *Archaeoroncus* Ćurčić and Rađa, 2012 = *Roncus* L. Koch, 1873, syn. nov. Overall, our study suggests that karstic microhabitats promote diversification in soil- and cave-dwelling arthropods at all taxonomic levels, but also provide important refugia for invertebrates in past and present periods of environmental change.

## Introduction

The Dinaric Karst of the western Balkans is one of the world’s largest karstic areas and located in Mediterranean Basin biodiversity hotspot. This karstic area covers about 60,000 km^2^ and extends from north-eastern Italy through Slovenia, Croatia, Bosnia and Herzegovina, Serbia, the Kosovo, and Montenegro to Albania in the south-east, and comprises a network of more than 20,000 caves and pits^1^. The dynamic geo-climatic history of the Dinaric Karst has led to high habitat complexity in terrestrial^2^, freshwater^3^, and subterranean ecosystems^4^, thereby boosting *in-situ* speciation and lineage isolation^5,6^. With more than 900 obligate subterranean species and many short-range endemics, the region hosts a unique assemblage of subterranean fauna^7^ and is considered a primary hotspot for subterranean fauna^4^. Diversity in the Balkans is generally very high because the region has acted as a major refugium for both the flora and fauna during the Pleistocene glacial cycles^8^, supporting relict species^9^ and recently evolved karstic lineages^10^. Most studies on the karstic biota in the Balkans have focused on flagship aquatic taxa such as world’s largest cave amphibian, the olm *Proteus anguinus* Laurenti, 1768^11^, and subterranean amphipod crustaceans^12^. The status of cryptic taxa^13,14^, especially terrestrial invertebrate fauna^15^ is often unclear and hampers a deeper understanding of evolutionary processes that lead to high diversity in this biodiversity hotspot.

**Figure 1:**
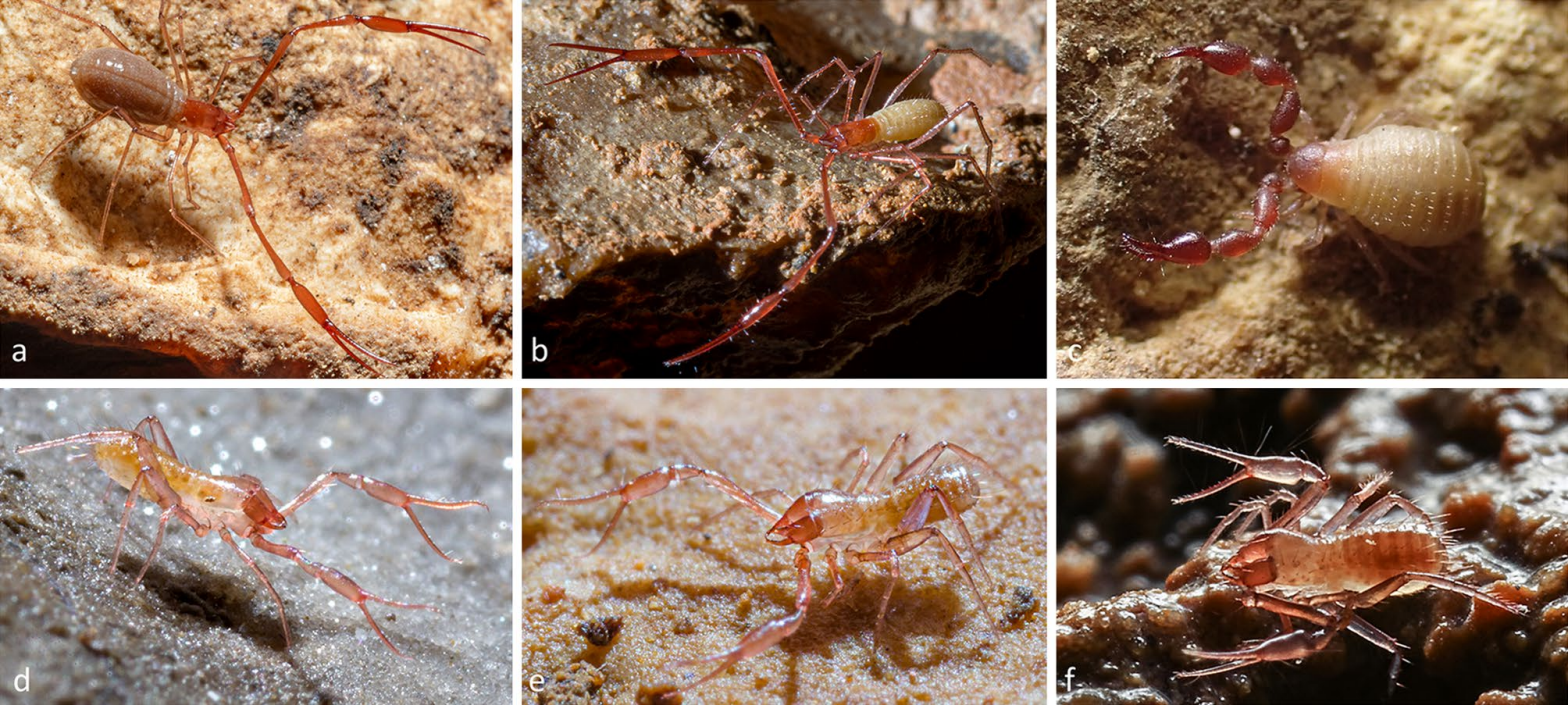
Life habitus of species within the most speciose families in the Dinaric Karst. (**a**, **b**) Neobisiidae Chamberlin, 1930; (**c**) Chernetidae Menge, 1855; (**d**, **e**, **f**) Chthoniidae Daday, 1889. Photos are courtesy of Petra Bregović (**a**, **b**, **d** and **e**), Branko Jalžić (**c**), and Tin Rožman (**f**).

Pseudoscorpions (Arachnida: Pseudoscorpiones de Geer, 1778) represent such a lesser-known terrestrial invertebrate lineage^16^, despite their extraordinary diversity^17^, with more than 100 described endemics in subterranean habitats across the Dinaric Karst. Many pseudoscorpions have poor dispersal abilities, small distributional ranges, and specific habitat requirements^18^. The order has a high proportion of subterranean endemics across the world and these species often show strong troglomorphic adaptations such as full-eye regression, elongated appendages, and loss of body pigment (Fig. 1). Some species can even be found in both subterranean and surface habitats^19^, thereby providing ideal systems to study aspects of subterranean diversification and processes leading to endemism.

The most recent checklist of Croatian pseudoscorpions^20^ lists 99 species and 10 subspecies. Since 2004 additional species have been described, and presently there are 126 species and 21 subspecies in 29 genera listed in the World Pseudoscorpiones Catalog^21^, of which 84 species and subspecies have their type locality in Croatia. The taxonomic literature pertaining to Croatian pseudoscorpions is fragmentary and contains incomplete distributional data, is biased towards single-specimen descriptions, and is burdened with taxonomic redundancy and radical changes in generic classification schemes^17,22^. The type specimens of many Dinaric species are either part of private collections, lost, or have not been deposited in the designated depositories as stated in their original descriptions^23–25^, thereby impeding or even completely preventing taxonomic work. However, the application of DNA barcoding in conjunction with species descriptions offer an avenue towards reevaluating species diversity in the Dinaric Karst both morphologically and genetically.

While DNA barcoding is known to facilitate rapid identification of specimens in the presence of accurate, high quality, and deep coverage reference sequences of described species^26^, there are obvious limitations when dealing with genetically understudied groups such as pseudoscorpions. Currently, the BOLD database contains more than 2700 pseudoscorpion sequences and of these only 1523 (representing 356 species) have been identified to species level (accessed 04 June 2022). The unidentified sequences are classified as “dark taxa”^27^ and do not correspond to any current morphospecies. Although DNA barcoding is often applied for biodiversity assessment using BOLD’s unique identifiers called BINs (Barcode Index Numbers)^28^, the use of a single (*COI*) gene also often leads to over-splitting of species^29^.

In the present study, we provide the first insights into patterns of genetic (inter- and intraspecific) diversity and endemism for pseudoscorpions from the Dinaric Karst using *COI* barcodes and concatenated datasets with the *28S* nuclear ribosomal RNA gene. We hypothesize that: (i) isolation in karstic microhabitats across the Dinaric Karst promotes diversification and speciation in pseudoscorpion lineages, (ii) genetic diversity might be higher than the current morphological taxonomy suggests, and (iii) patterns of endemism and species distribution correspond to geomorphological features. To test these, we created an extensive DNA barcode reference library comprising 499 lineages (455 hypogean and 44 epigean) to: (i) test current morphological species concepts and known distributional ranges using a combination of morphology and genetics, (ii) estimate optimal identification thresholds for species delineation in Dinaric pseudoscorpions, and (iii) identify refugia with high genetic and taxonomic diversity for conservation management.

## Materials and methods

### Sampling and taxonomic assignment

A total of 2015 pseudoscorpion specimens from 435 localities in Croatia (including 249 specimens representing type localities of 20 species), and 181 specimens from 49 localities in Bosnia and Herzegovina, and Montenegro were collected and preserved in 96% ethanol. Specimens were collected from both surface and subterranean habitats, including 1985 specimens from the Dinaric Karst. For morphological identification, adult specimens were cleared in 70% lactic acid if necessary and examined to morphospecies level using Leica M205 C stereomicroscope and Leica DM2500 compound microscope and relevant taxonomic literature^30–34^. Whenever possible, specimens were compared with topotypic vouchers (those collected from or near the type locality). Specimens were also re-checked against the literature if conventional morphology and DNA barcoding results mismatched. The abbreviation aff. was used for specimens that were morphologically close to described species but did not entirely match the species diagnoses. Images were taken using Canon EOS 7D Mark II attached to a BK PLUS Lab System by Dun, Inc., with the software Capture One Pro 9.3 64 Bit ver. 9.3.0.85 and stacked using Zerene stacker (Zerene Systems LLC 2016). Details about the specimens are listed in Supplementary Table 1 online.

### Molecular methods

**Figure 2:**
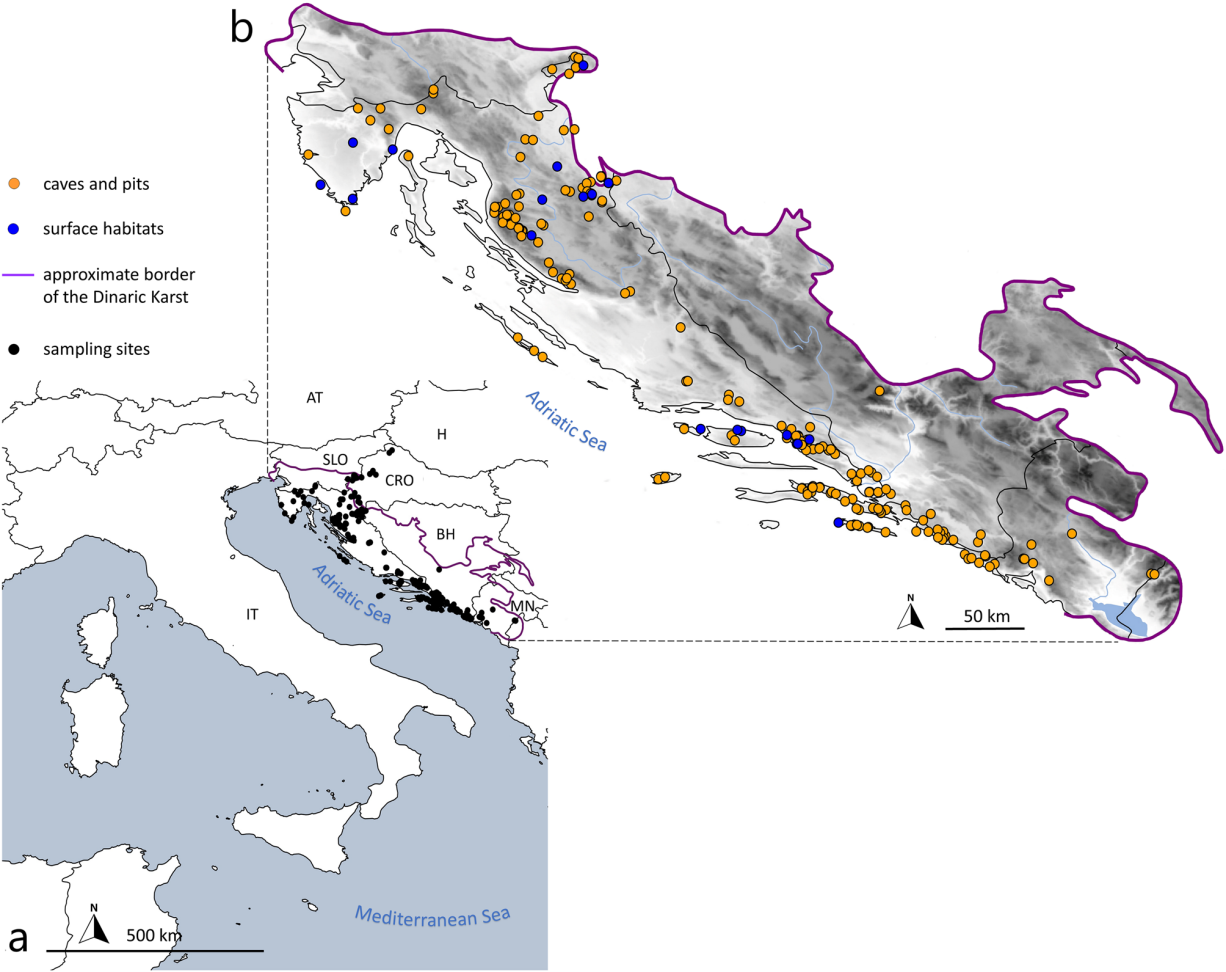
Distribution of georeferenced DNA barcode records is represented with black dots on the map (a). The main map (**b**) is an enlarged Dinaric Karst area marked in the bottom left corner (**a**). For locality references see Supplementary Table 1 online. Approximate border of the Dinaric Karst according to^35^. Map is produced with Cartopy package ver. 0.19 (https://scitools.org.uk/cartopy/docs/v0.18/index.html) in Python ver. 3.8 with use of elevation data from^36^. Abbreviations: IT Italy, AT Austria, SLO Slovenia, CRO Croatia, H Hungary, BH Bosnia and Herzegovina, MN Montenegro.

For DNA extraction, we selected a total of 550 individuals from 219 localities (Fig. 2), including 61 specimens from 20 type localities, together with 23 specimens from Bosnia and Herzegovina, and Montenegro, representing morphological variability between specimens and multiple collection events. Genomic DNA was extracted using the QIAamp DNA Micro Kit (Qiagen, Hilden, Germany) following the standard protocol, except that 50 µl of elution buffer was used to increase DNA yield. DNA was extracted from 1 or 2 legs, and additional legs were used if samples were old or in poor condition. Polymerase chain reactions (PCRs) were performed to amplify the 658 base pairs (bp) barcode region of the mitochondrial gene cytochrome *c* oxidase subunit I (hereafter “*COI*”)^26^. Additionally, ca. 900 bp of the domain I region of the *28S* nuclear ribosomal RNA gene (hereafter “*28S*”) was amplified for closely related *COI* lineages within the most diverse families Chthoniidae (41 individuals) and Neobisiidae (48 individuals) to test the monophyly of recognized morphospecies using a non-mitochondrial marker. For details on PCR protocols see Supplementary Table 2 online. Bi-directional sequencing was done by Macrogen Inc. (Amsterdam, The Netherlands).

### Sequence data authentication

All sequence chromatograms were manually edited in Geneious Prime 2022.1 (Biomatters, Auckland, New Zealand). Quality control included checking the sequences for double peaks, stop codons, amino acid translations using Mesquite ver. 3.61^37^, and BLAST searches to check for contamination. Sequences were aligned using MAFFT ver. 7^38^ with the “auto” strategy for *COI* datasets, while the E-INS-I algorithm with a 1PAM/k = 2 scoring matrix and the highest gap penalty was used for *28S* datasets. A dataset consisting of 499 successfully obtained *COI* sequences was compiled and will heretofore be referred to as the “Dinaric dataset” (Alignment 1). The Dinaric dataset was collapsed into haplotypes using the online tool FaBox ver. 1.61^39^.

### Phylogenetic inference

**Figure 3:**
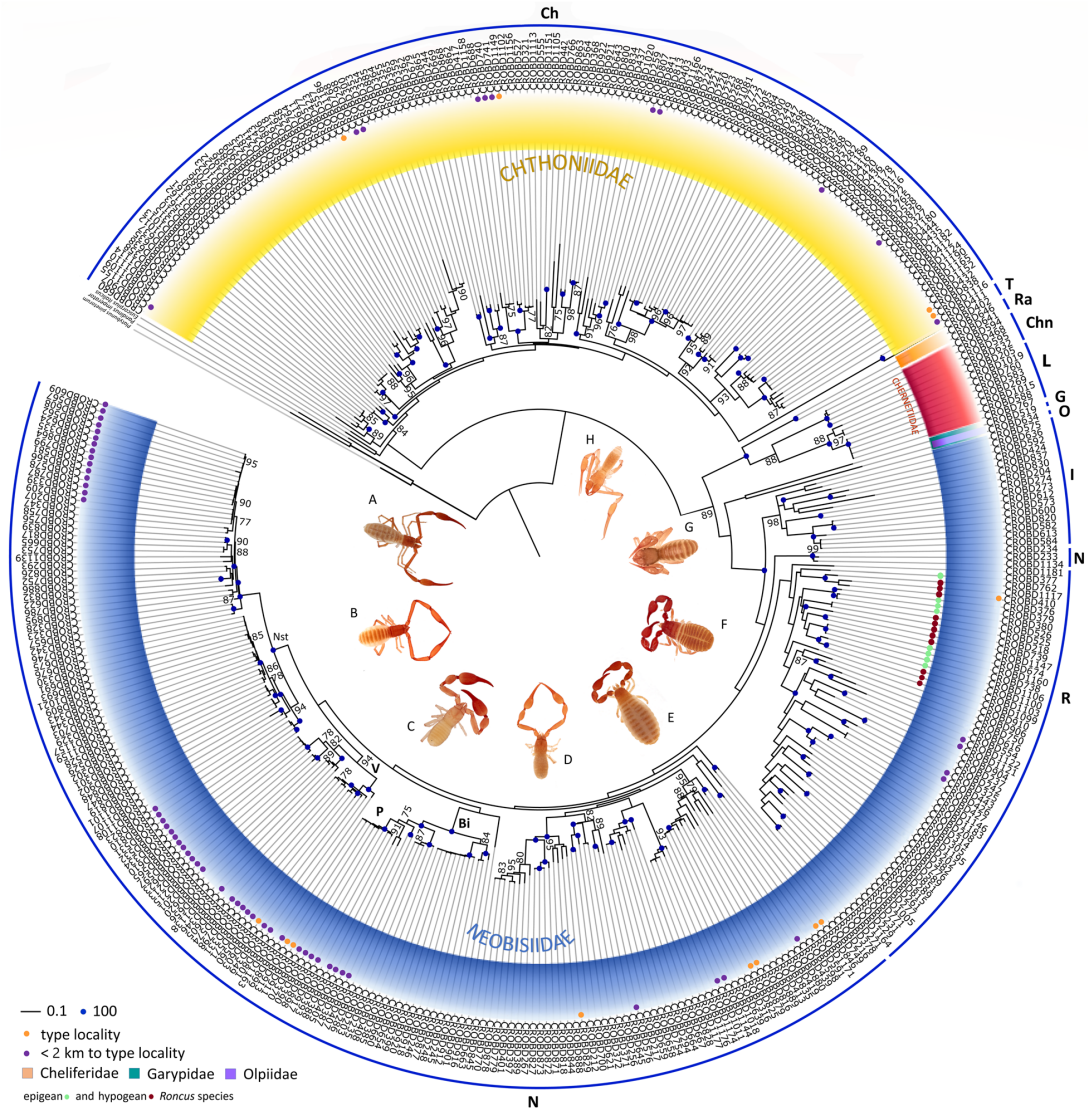
Circular ML tree. Species are colour-coded by family. Numbers on nodes represent ultrafast bootstrap support values (BS). Terminal codes present Sample IDs in BOLD, as in Supplementary Table 1 online. The tree was annotated in FigTree ver. 1.4.3^45^ and for the circular visualization finished in iTOL ver. 5^46^ and Adobe Illustrator. Representative pseudoscorpions of most recognized genera from the Dinaric Karst are shown in the middle: (**a**) *Neobisium vjetrenicae* Hadži (Neobisiidae), (**b**) *Neobisium staudacheri* Hadži (Neobisiidae), (**c**) *Roncus ragusae* Ćurčić (Neobisiidae), (**d**) *Insulocreagris* sp. (Neobisiidae), (**e**) *Pselaphochernes litoralis* Beier (Chernetidae), (**f**) *Lasiochernes siculus* Beier (Chernetidae), (**g**) *Chthonius* sp. (Chthoniidae), (**h**) *Chthonius magnificus* Beier (Chthoniidae). Different genera are represented with blue bars and labelled as: Ch (*Chthonius*), T (*Troglochthonius*), Ra (*Rhacochelifer*), Chn (*Chernes*), L (*Lasiochernes*), G (*Garypus*), O (*Olpium*), I (*Insulocreagris*), R (*Roncus*) and N (*Neobisium*). Abbreviations: P (*Protoneobisium*), Nst (*Neobisium stygium*), V (Velebit Mountain) and Bi (Biokovo Mountain).

Phylogenetic analyses were performed using a maximum likelihood (ML)^40^ approach in IQ-TREE ver. 2.0.3^41^ with 5000 ultrafast bootstraps^42^ for: (i) the Dinaric dataset and additional sequences from central Europe^43^ to check species-level determination (Supplementary File 1 online), and ii) Dinaric *COI* haplotypes only to select taxonomically relevant sequences for detailed analyses (Fig. 3). Ultimate outgroups for both datasets included two scorpions: *Pandinus imperator* (C.L. Koch, 1841) (AY156582) and *Euscorpius italicus* (Herbst, 1800) (AMSCO005-10), considering the sister-group relationship between scorpions and pseudoscorpions^44^. All datasets were rooted against the harvestman *Platybunus pinetorum* (C. L. Koch, 1839) (GBBSP1395-15).

To resolve phylogenetic relationships among closely related *COI* lineages, we further generated two concatenated (*COI-28S*) alignments: one for the family Chthoniidae (“concatenated Chthoniidae subset”: 41 individuals, Alignment 2) and one for the Neobisiidae (“concatenated Neobisiidae subset”: 48 individuals, Alignment 3), including sequences available in GenBank (Supplementary Table 3 online). We removed gap-rich regions from the *28S* sequences in Gblocks ver. 0.91b^47^ using settings for less stringent selection and generated concatenated alignments 2a for Chthoniidae and 3a for Neobisiidae. For these four datasets (Alignments 2, 3, 2a and 3a), we constructed phylogenetic trees with both ML and Bayesian inference (BI) methods. To determine node support for ML trees within IQ-TREE, we used 5000 ultrafast bootstraps and 2000 Shimodaira-Hasegawa-like (SHL) approximate likelihood ratio test replicates^48^. BI was performed using MrBayes ver. 3.2.7^49^ with the optimal substitution models determined using PartitionFinder ver. 2.1.1^50^ (Supplementary Table 2 online). The complete dataset was partitioned by gene and codon positions (for *COI*). Two parallel runs each comprising four Markov chain Monte Carlo (MCMC) were run simultaneously for 30 million generations, with every 1000^th^ tree sampled. Resulting log-files were analyzed in Tracer ver. 1.7.1^51^ to check for stationarity of parameters, and the first 25% of sampled trees were discarded as burn-in. All analyses were executed on the CIPRES Science Gateway^52^.

### Efficiency of DNA barcoding for identification

Two additional *COI* alignments for the most diverse families (Alignment 4 for Dinaric Chthoniidae and Alignment 5 for Dinaric Neobisiidae) were generated to calculate pairwise nucleotide distance matrices using the Kimura’s 2-parameter (K2P)^53^ in the package *ape*^54^ in R ver. 3.5.2^55^. Matrices were further used for the estimation of optimal threshold (OT) using *threshold optimization* analysis in the library *spider* ver. 1.4–2^56^. After preliminary analyses we explored a range of threshold values (3–11%) that minimized cumulative error (false positive + false negative). The estimated OT was then used in the *Best Close Match* (BCM) analysis^57^ and implemented in *spider* library to evaluate identification efficiency of Dinaric Chthoniidae and Neobisiidae at the genetic level. All singletons were removed from these analyses.

### Inter- and intraspecific diversity

**Figure 4:**
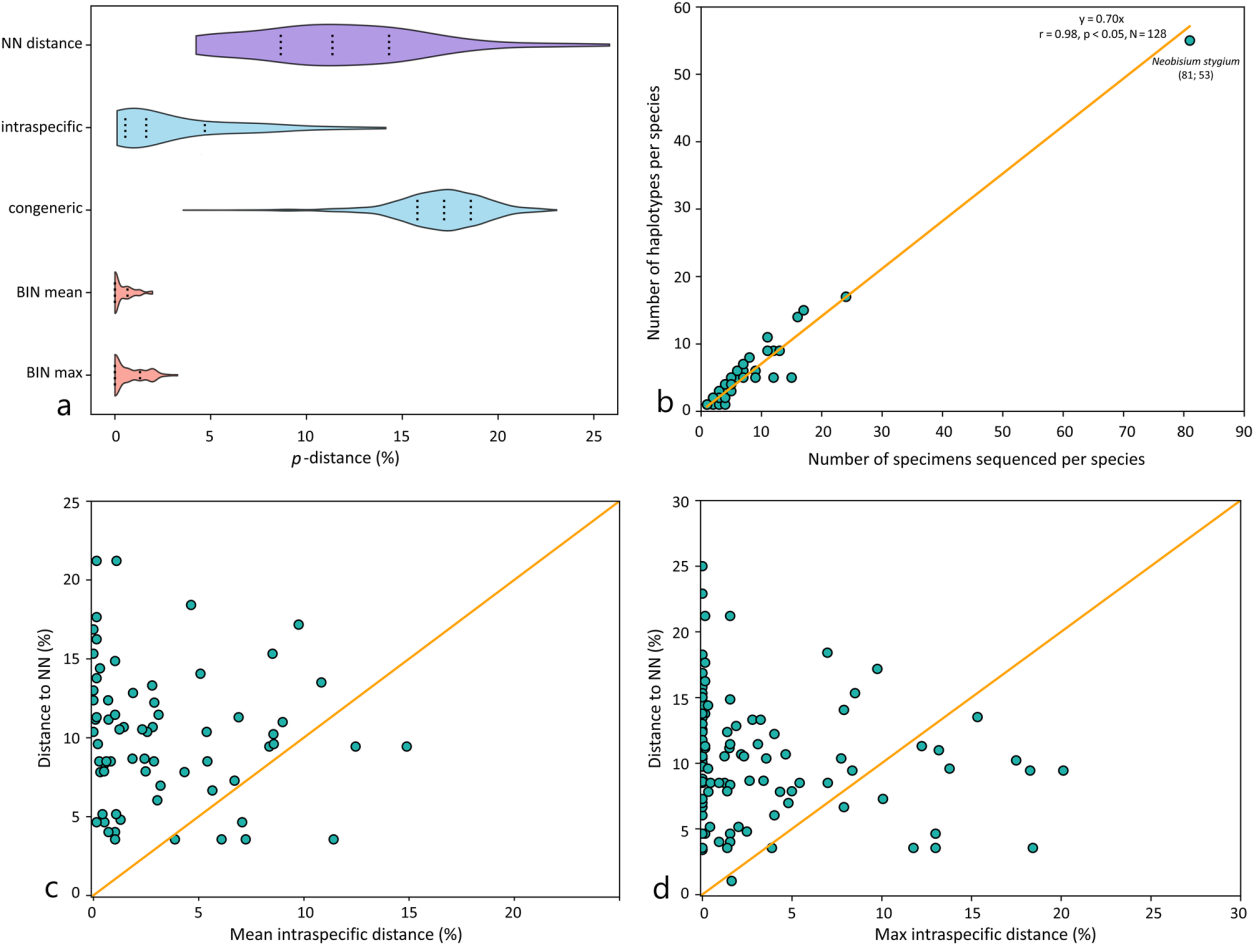
Intra- and interspecific diversity of pseudoscorpion species. Missing data was treated with pairwise deletion option. (**a**) Violin plot representing nearest-neighbour (NN), mean intraspecific and congeneric genetic distances, and BIN mean and maximal distances across all species in the dataset. The dashed lines inside indicate the first, middle, and third quartiles of the data. (**b**) Number of distinct haplotypes per species vs. specimens’ sequences per species. Correlation equation, Pearson correlation coefficient, probability (*p*), and species number are shown above graph. *Neobisium stygium*, most common species is indicated in the graph (number of specimens; number of haplotypes); (**c**) Mean intraspecific distances vs. minimum interspecific distances; (**d**) Maximal intraspecific distances vs. minimum interspecific distances.

BOLD’s *Barcode Gap Analysis* was used to ascertain the existence of a barcoding gap, analyze mean and maximum intraspecific variation, and identify minimum genetic distances to the nearest-neighbour, using the *p*-distance model and pairwise deletion option (Fig. 4a–d, Supplementary Table 4 online) and excluding species represented by singletons. Concordance between *COI* sequence clustering and morphospecies concepts within the Dinaric dataset and the two concatenated datasets (Chthoniidae and Neobisiidae subsets) was explored using three distance-based and one tree-based delimitation approaches: Automatic Barcode Gap Discovery (ABGD)^58^, Assemble Species by Automatic Partitioning (ASAP)^59^, BIN assignments^28^, and the Bayesian implementation of the Poisson tree process (bPTP)^60^. For details, see Supplementary Methods online.

Based on the Chthoniidae (Alignment 4) and Neobisiidae (Alignment 5) *COI* alignments, we analyzed the spatial patterns of mitochondrial genetic diversity (Supplementary Fig. 1a–b online) using uncorrected pairwise distances (*p*-distances) as calculated in MEGA-X ver. 10.2.6^61^ with the pairwise deletion option. Six sequences (shorter than 500 bp) were excluded from the distance calculation. For geodesic distance calculation, we used an algorithm described in^62^, as implemented in GeoPy Python module ver. 2.2.0.

**Figure 5:**
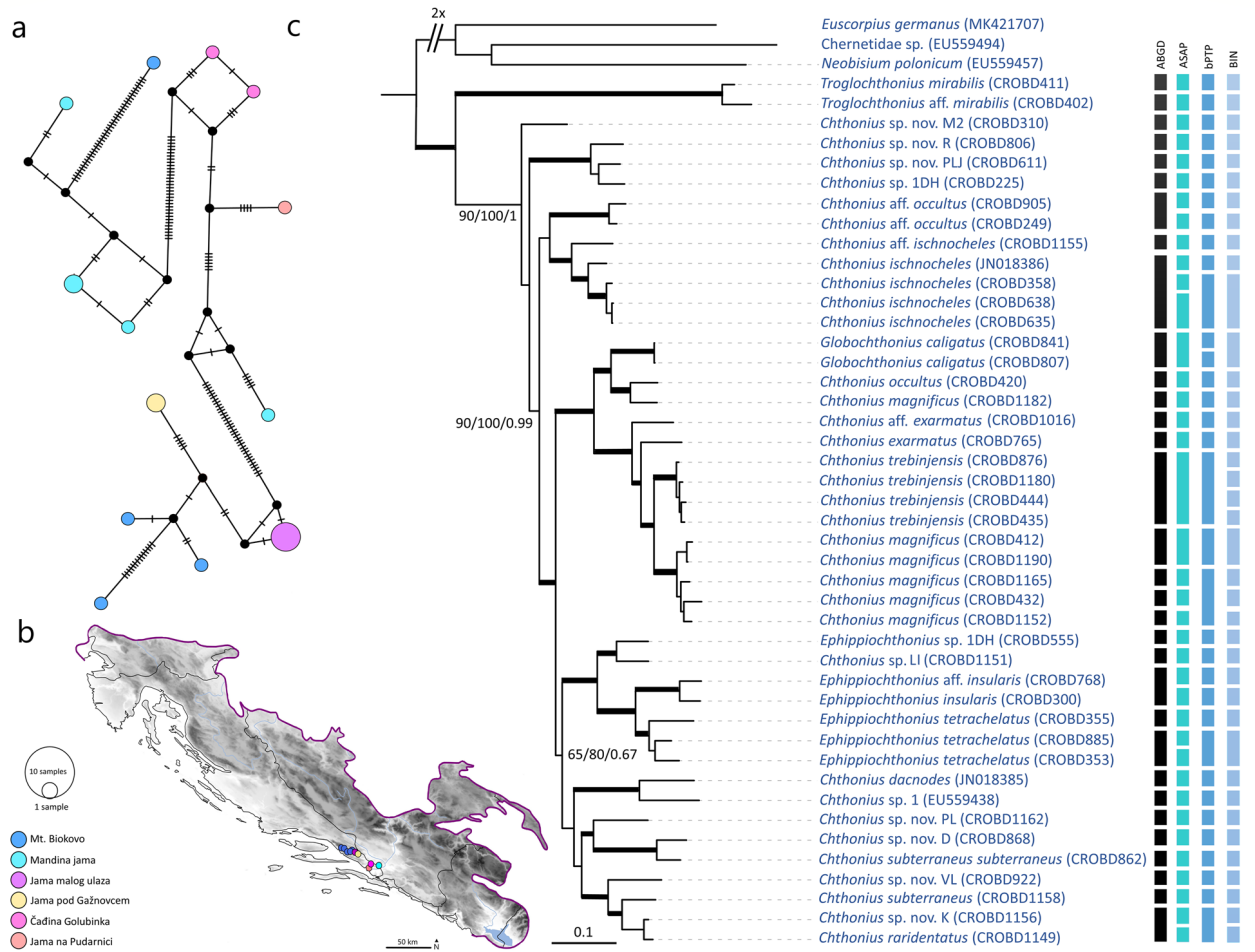
Median-joining haplotype network for *Chthonius* aff. *occultus* and ML phylogram of the concatenated *COI-28S* Chthoniidae subset with species delineation results. (**a**) Median-joining haplotype network for *Chthonius* aff. *occultus* haplotypes based on the *COI* gene. Haplotype circles are colour-coded according to localities and circle size is proportional to overall haplotype frequency. Black dots indicate the extinct ancestral or unsampled haplotypes. Numbers of mutational steps are given as hatch marks. (**b**) Sampling localities (colour coding matches insert in Fig. 5a). Map was made using Cartopy package ver. 0.19 (https://scitools.org.uk/cartopy/docs/v0.18/index.html) in Python ver. 3.8. (**c**) Node support is reported as ultrafast bootstrap resampling frequencies (BS), followed by the Shimodaira-Hasegawa-like (SHL) approximate likelihood ratio and posterior probabilities (PP). Branches with ultrafast bootstrap support value (100), SHL approximate likelihood ratio (100) and posterior probabilities (1) were shown in boldface. Morphospecies were mapped onto the terminals of the ML along with the Sample IDs as in Supplementary Table 1 online. Colour strips refer to species delimitation results (MOTUs) as indicated by ABGD, ASAP, bPTP, and BIN assignments.

**Figure 6:**
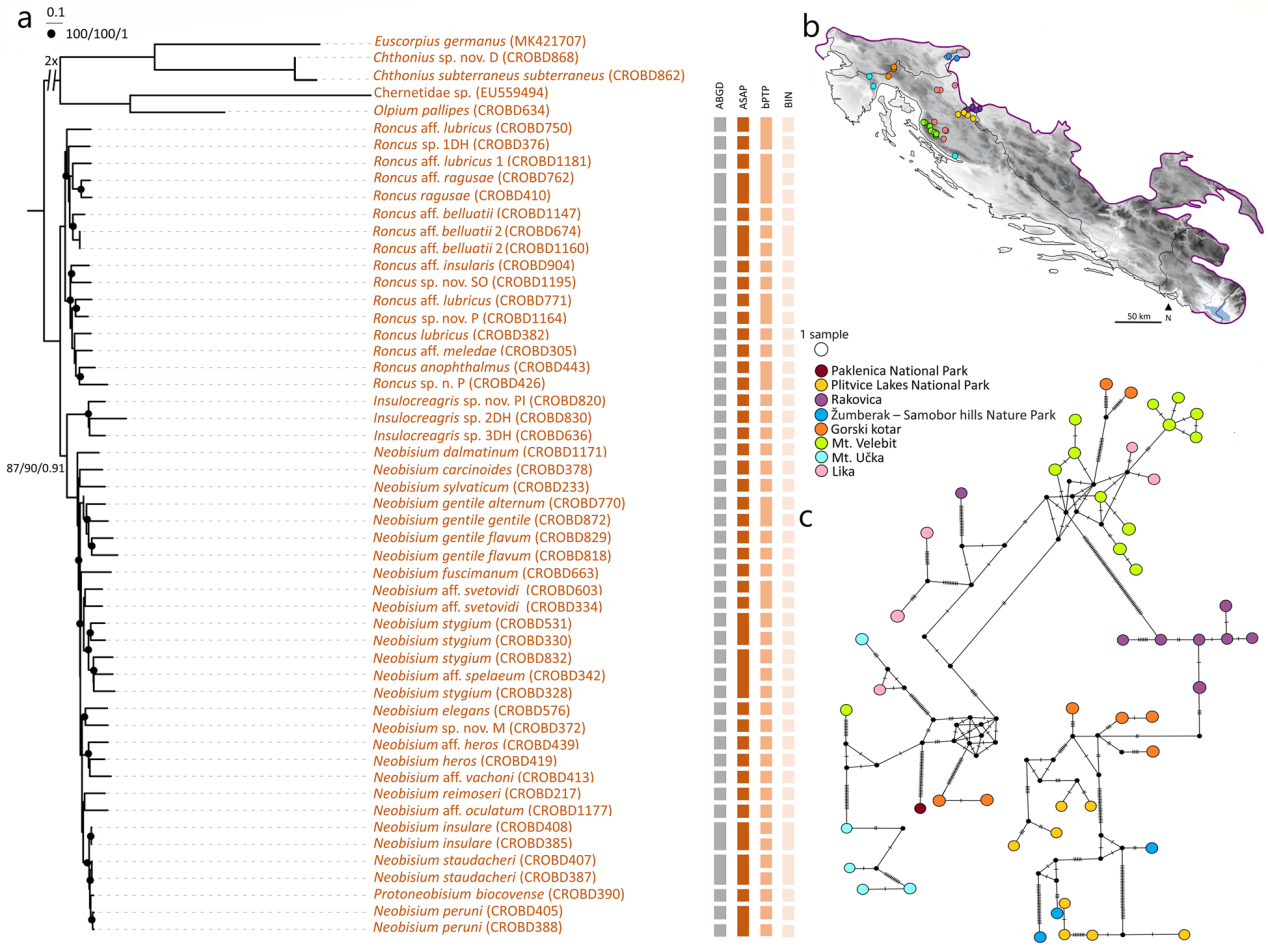
ML phylogram of the concatenated *COI-28S* Neobisiidae subset with species delineation results, and median-joining haplotype network for *Neobisium stygium*. (**a**) Node support is reported as ultrafast bootstrap resampling frequencies (BS), followed by the Shimodaira-Hasegawa-like (SHL) approximate likelihood ratio and posterior probabilities (PP). Branches with ultrafast bootstrap support value (100), SHL approximate likelihood ratio (100) and posterior probabilities (1) were marked with black dots. Morphospecies were mapped onto the terminals of the ML along with the Sample IDs as in Supplementary Table 1 online. Colour strips refer to species delimitation results (MOTUs) as indicated by ABGD, ASAP, bPTP, and BIN assignments. (**b**) Sampling localities (colour coding matches insert in Fig. 6c). Map was made using Cartopy package ver. 0.19 (https://scitools.org.uk/cartopy/docs/v0.18/index.html) in Python ver. 3.8. (**c**) Median-joining haplotype network for *Neobisium stygium* based on the *COI* gene. Haplotype circles are colour-coded according to localities and circle size is proportional to overall haplotype frequency. Black dots indicate the extinct ancestral or unsampled haplotypes. Numbers of mutational steps are given as hatch marks.

To visualize haplotype relatedness for species with genetic structuring across their distributional ranges, median joining networks (MJ) were reconstructed using PopART ver. 1.7^63^. These networks included *Chthonius* aff. *occultus* Beier, 1939 (Fig. 5a,b) and *Neobisium stygium* Beier, 1931 (Fig. 6b,c). Additionally, we examined relationships among populations of *Neobisium sylvaticum* (C.L. Koch, 1835) from Croatia and Germany, using published sequences^43^ (Supplementary Fig. 2 online).

### Quantification of biodiversity patterns

**Figure 7:**
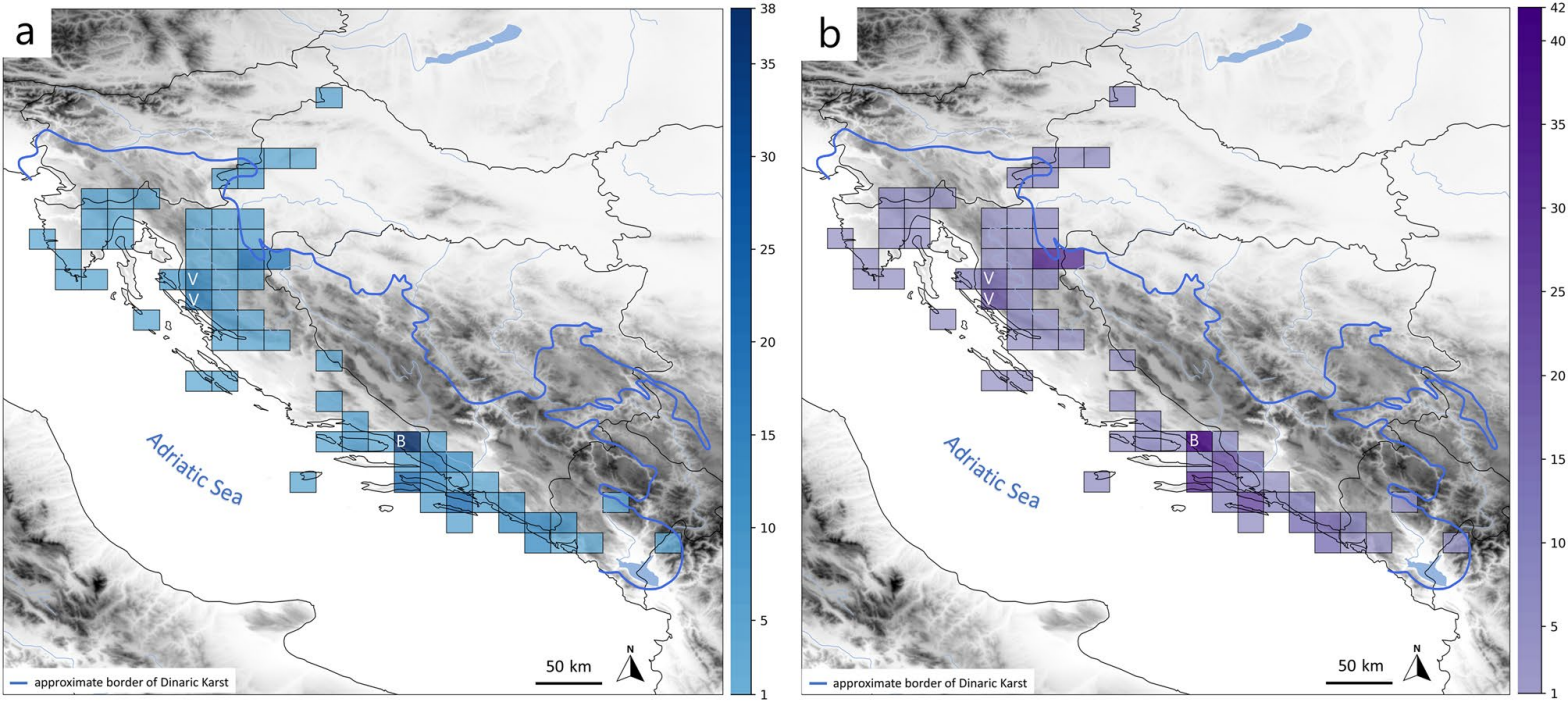
Species richness. (**a**) Species richness as sum of morphospecies. (**b**) Species richness as number of MOTUs in each cell. Areas without cells represent missing data. Abbreviations: V and B (Velebit and Biokovo Mountains). Map was made using Cartopy package ver. 0.19 (https://scitools.org.uk/cartopy/docs/v0.18/index.html) in Python ver. 3.8.

Taxonomic diversity (species richness) was plotted onto a raster of the study area by overlaying the sum of identified morphospecies (Fig. 7a) and the sum of molecular operational taxonomic units (MOTUs) obtained by BOLD (Fig. 7b). We used a grid-based approach and quadratic cells with 20 × 20 km resolution, as in previous studies of the Dinaric subterranean fauna^64^. Graphs and maps were made in Matplotlib ver. 3.5.2.^65^ and the Cartopy package ver. 0.19 in Python ver. 3.8.

## Results

### Conventional taxonomy

Using a combined approach of morphology and genetics, we identified 128 morphospecies in 15 genera and six families, collected from 219 localities (Fig. 2). A total of 282 specimens matched the original descriptions of 50 known species and subspecies, but 111 specimens (31 morphospecies) were assigned with the prefix "aff." since they deviated slightly from the morphological species diagnoses. An additional 106 specimens did not match any morphological description and are treated here as 47 putative new species from 74 sampling localities (32 of them being single-site endemics). For 40 described species and subspecies (251 samples), we provide the first molecular characterization. The most diverse genera were *Chthonius* (37 morphospecies) and *Neobisium* (34 morphospecies). Thirty-three specimens represented five genera endemic to the Dinaric Karst: *Archaeoroncus* Ćurčić and Rađa, 2012, *Insulocreagris* Ćurčić, 1987, *Microchthonius* Hadži, 1933, *Protoneobisium* Ćurčić, 1988, and *Troglochthonius* Beier, 1939. There were two new records for Croatia: species *Chernes hahnii* (C.L. Koch, 1839) and genus *Lasiochernes* Beier, 1932.

### Sequence statistics

The Dinaric dataset was collapsed to 396 haplotypes, contained no ambiguous positions or internal stop codons, and had a median length of 655 bp (range 349–658 bp). Sequences belonging to the families Neobisiidae and Chernetidae possessed a single amino acid deletion, as previously reported^66^. Haplotype diversity was correlated with the number of individuals sequenced per species (Fig. 4b), with an average of 3.2 haplotypes per species.

The *28S* alignment had no base polymorphisms and was 1192 bp (including gaps) and 919 bp (soft trimmed) in length for Chthoniidae, whereas it was 1183 bp and 941 bp in length, respectively for Neobisiidae.

### Phylogenetic analyses

Phylogenetic analyses of the *COI* gene resulted in well-resolved trees, with most clades supported by bootstrap values ≥ 95, and generally supporting family, generic, and species hypotheses (Supplementary File 1 online, Fig. 3). Preliminary analyses revealed distinct mitochondrial lineages that could not be assigned to described morphospecies (Supplementary File 1 online). In the Dinaric *COI* tree, two unresolved phylogenetic relationships were observed: polytomy consisting of the *Troglochthonius* and *Chthonius* species (see clade T, Fig. 3), and unresolved species hypotheses within the genus *Insulocreagris* (see clade I, Fig. 3). Most genera and species were recovered as monophyletic, except for the genus *Globochthonius* Finnegan, 1932 (polyphyletic) and the species *Roncus lubricus* L. Koch, 1873 (polyphyletic).

The use of the concatenated *COI-28S* subsets helped in resolving deeper relationships within the diverse families Chthoniidae and Neobisiidae and resolved polytomies from the Dinaric *COI* tree. Since the tree topologies obtained from the BI and ML analyses of both the Chthoniidae and Neobisiidae concatenated subsets (untrimmed and soft trimmed) were mostly consistent, we present only the ML results of the untrimmed subsets (Figs. 5c, 6a).

### Interspecific genetic variability and *COI* species delineation

Based on the BOLD’s *Barcode Gap Analysis*, a DNA barcoding gap was obtained in 51, but not found in 15 species pairs (Fig. 4d), representing morphologically diagnosed but genetically closely related *Chthonius*, *Neobisium* and *Roncus* species.

An OT of 4.7% K2P nucleotide distance was estimated as an optimal identification threshold for the Dinaric Chthoniidae (based on 106 sequences), and 3.6% for the Dinaric Neobisiidae (285 sequences), respectively. The BCM analysis correctly identified 104 Chthoniidae sequences, yielding an efficiency of 98.1%. For Neobisiidae, the BCM analysis correctly identified 277 sequences and produced an efficiency of 97.2%. For both families, the *COI* gene showed high genetic diversity even at a small spatial scale (Supplementary Fig. 1 online).

None of the molecular species delimitation methods inferred the same number of species as morphospecies assignment. For the Dinaric dataset, ABGD delimited 155 putative species, while ASAP (with the best ASAP-score 11.50) inferred 130 putative species and agreed most with morphological identifications. The bPTP method inferred the highest with 199 putative species. Using the BOLD’s BINs, sequences were allocated to 236 BINs of which 232 were unique, while BIN counts were 1.8 times higher than traditionally recognized morphospecies counts. One case of BIN sharing was observed (*Chthonius* sp. ZB and *Chthonius* sp. LI). In total, 133 BINs represented single individuals (singletons) and there is clearly a need for examining additional specimens to estimate true BIN diversity within the Chthoniidae and Neobisiidae. ABGD, bPTP and BIN assignments altogether split the species *Neobisium stygium*, *Roncus lubricus* and *Chthonius magnificus* Beier, 1938, mostly according to collection sites. For detailed results of applied species delineation methods, see Supplementary Table 1 and Supplementary Table 5 online.

For *COI* sequence clustering within the concatenated Chthoniidae subset (29 morphospecies), the ABGD method delineated 32 putative species that were mostly congruent with morphological identifications (Fig. 5c). ASAP, with the best ASAP-score (1.50), indicated 38 putative species, bPTP recovered 34 putative species, and the BOLD’s assignment resulted in 39 BINs. For the concatenated Neobisiidae subset (39 morphospecies), ABGD delimited 44 putative species, ASAP, with the best ASAP-score (3.50) predicted 40 putative species, bPTP resulted in 41 species, and the BOLD assignment revealed 45 BINs (Fig. 6a). Overall, for all datasets, ABGD and ASAP provided conservative estimates of putative species, while bPTP and BIN assignments indicated potential over-splitting when compared with morphology.

### Intraspecific genetic variability

Within the Dinaric dataset, the mean intraspecific *p*-distance was 3.5% and overlapped slightly with the distance to the nearest-neighbour (NN) (Fig. 4a). The highest values of intraspecific *p*-distances were obtained for seven cave-dwelling species: *Chthonius* aff. *absoloni* Beier, 1938 (11.7%), *Chthonius* sp. ZB (14.4%), *Neobisium* aff. *spelaeum* (6.1%), *Neobisium* aff. *svetovidi* Ćurčić, 1988 (7.0%), *Neobisium stygium* (7.2%), *Roncus* aff. *lubricus* (14.9%), and *Roncus lubricus* (12.6%). Large intraspecific distances generally corresponded with high haplotype diversity within these species, indicating geographical isolation of localized populations. The most common species, *Neobisium stygium*, was represented by 81 specimens and 53 haplotypes (Fig. 6b,c).

The two Dinaric species with broader distributions, *Chthonius* aff. *occultus* and *Neobisium stygium*, both show deep intraspecific splits and complex phylogeographic structuring across their range, without haplotype sharing between sampling localities (Figs. 5a,b, 6b,c). Deep intraspecific splits were also observed in eight cave-dwellers (*Chthonius* aff. *alpicola* Beier, 1951, *Chthonius* aff. *occultus*, *Chthonius magnificus*, *Ephippiochthonius insularis* (Beier, 1938), *Globochthonius caligatus* (Beier, 1938), *Neobisium gentile alternum* Beier, 1939, *Neobisium gentile gentile* Beier, 1939, *Neobisium reimoseri* (Beier, 1929)) and 5 surface-dwellers (*Roncus italicus* (Simon, 1896), *Ephippiochthonius tetrachelatus* (Preyssler, 1790), *Rhacochelifer maculatus* (L. Koch, 1873), *Chthonius raridentatus* Hadži, 1930 and *Chernes hahnii*). Phylogeographic structuring was also observed in the epigean species, *N. sylvaticum* (Supplementary Fig. 2 online), which is widely distributed in Europe^21^.

## Discussion

This study represents the first comprehensive DNA barcode reference library of pseudoscorpions from the Dinarides, including 499 *COI* barcodes from 128 morphospecies and a high proportion of endemic, rare and elusive taxa, thereby filling an important gap in biodiversity assessment across this region. The very high identification efficiencies (98.1 and 97.2% for Dinaric Chthoniidae and Neobisiidae, respectively), as estimated from BCM analyses, suggest that DNA barcoding is an effective approach to identify many morphospecies based on *COI* divergences. The high success rate of this technique us comparable to other studies involving arachnid groups globally, including eriophyoid mites (99%;^67^), feather mites (100%;^68^) and spiders (98%;^69^).

Our analyses resulted in low optimal identification thresholds for Dinaric pseudoscorpions (4.7 and 3.6% for Chthoniidae and Neobisiidae, respectively), suggesting that the Dinaric lineages represent evolutionarily young and morphologically distinguishable sister-taxa^70^. DNA barcoding, however, is known to fail in species delineation when a high proportion of species are either closely related allopatric or parapatric taxa, or are known to hybridize^70,71^. This might also have affected the Dinaric dataset to some degree since the DNA barcoding gap was not obtained for 15 pairs of morphospecies (Fig. 4d). On the contrary, a significant DNA barcoding gap was obtained for 51 species-pairs that can be the result of insufficient sampling, at both the interspecific and intraspecific level. Sampling the subterranean matrix and cryptic taxa such as pseudoscorpions is difficult and collecting biases are almost inevitable because the entire distribution of most taxa is either unknown or cannot be covered by field collections.

This study further shows that the high levels of taxonomic diversity and endemism in the Dinarides, both at the generic and species level, are also recovered by genetic data and not the result of taxonomic over-splitting. The ASAP method, which is not based on *a priori* defined maximal genetic intraspecific divergences, was in general agreement with established morphospecies concept and revealed 130 MOTUs compared with 128 identified morphospecies. Conversely, the ABGD, bPTP and BIN assignments showed over-splitting in species such as *Neobisium stygium*, *Chthonius* aff. *absoloni*, *Chthonius magnificus* and *Roncus lubricus.* It splitted morphospecies according to their sampling localities. The ABGD method, which assumes that interspecific genetic distances are higher than intraspecific divergences, also encountered difficulties in differentiating among species. It recovered *Neobisium insulare* Beier, 1938, *Neobisium staudacheri* Hadži, 1933, *Protoneobisium biocovense* (Müller, 1931) and *Neobisium maderi* Beier, 1938 in one group, and *Neobisium* aff. *vachoni* Heurtault, 1968, *Neobisium dinaricum* Hadži, 1933, *Neobisium vjetrenicae* Hadži, 1932 in a second group, while all other delineation methods were able to delineate them as distinct species. Lumping these species indicates a major limitation of this method, which is known to perform poorly on datasets comprising large numbers of evolutionarily young species and taxa with high diversification rates^72^, both of which are applicable to the Dinaric dataset.

Extensive subterranean radiation and speciation among Dinaric pseudoscorpions at a relatively narrow geographic scale can be at least partly attributed to vicariance in subterranean habitats, i.e., the presence of caves and pits and their colonization by a soil-dwelling fauna such as chthoniid and neobisiid pseudoscorpions. This is evident in the Dinaric dataset in which epigean *Roncus* species were sister to *Roncus* cave-dwellers (Fig. 3). Further, haplotype networks indicated population isolation for species with broad distributions in the Dinarides such as for *Chthonius* aff. *occultus* (Fig. 5b) and *Neobisium stygium* (Fig. 6b), which both have high numbers of mutational steps between lineages and lack shared haplotypes among localities. The presence of an extraordinarily rich karstic landscape resulted in high *COI* divergences in 15 cave-dwellers and 5 surface-dwellers, and karstification is obviously a major trigger for diversification in the Dinaric Karst. This agrees with previous studies of many other invertebrate taxa that show the Dinarides as a center of remarkable allopatric diversification^6,73,74^. Additionally, various zoogeographical divisions along the Adriatic coast, including coastal islands, and mountain ranges, can also drive speciation and act as potential microrefugia during periods of environmental change^15^. In our study, species richness was highest in the caves of the Velebit and Biokovo Mountains, and at the margin of karstic areas and alluvial plains in southern Croatia (Fig. 7). The highest number of morphospecies and MOTUs were 38 and 42 per cell, and were found in Biokovo Mountain, which is well-known for high endemism and the presence of a specialized subterranean fauna^75,76^. Both the Velebit and Biokovo Mountains are protruding ridges of limestone on subordinate dolomites near the coastline, and harbor two genetically well-supported clades of the genus *Neobisium* (see clades V and Bi, Fig. 3). Both areas are highly karstified with erosional processes and glacial activity during the Late Pleistocene^77^ and potentially earlier, providing new microhabitats for colonization and diversification during and since the European ice ages. Furthermore, these two massifs support neobisiid species (*Neobisium peruni* Ćurčić, 1988, *Neobisium staudacheri*, *Neobisium stribogi* Ćurčić, 1988 and *Protoneobisium biocovense*), which are amongst the largest pseudoscorpions in the world with a body length above 10 mm. The high intraspecific *COI* divergences obtained for 15 subterranean and 5 surface lineages found in isolated karstic microhabitats indicate incipient speciation and overlooked cryptic diversity^78^, driven at least in parts by natural selection in subterranean habitats^79^ or by genetic drift^78^.

Two “widespread” *Neobisium* species have complex phylogeographic signatures. *Neobisium stygium* (Fig. 6b) has a broader, north Dinaric distribution^80^, but falls into two geographically isolated clades (see Nst in Fig. 3), 53 haplotypes and 14 BINs. In this region, karstic substrates are porous and therefore not a limiting factor for species distribution within MSS (*Milieu Souterrain Superficiel*) habitat. Similar patterns in this area have been observed for other taxa such as the millipede, *Haasia stenopodium* (Strasser, 1966)^81^ and the beetle *Leptodirus hochenwartii* (Schmidt, 1832)^82^. The diversity of *Neobisium sylvaticum* at the genetic level is also remarkable and this species presently has a broad distribution in Europe but is almost certainly a cryptic-species complex. Although our geographic sampling did not cover the complete range of this species, we identified at least three genetically diverse phylogroups that are geographically isolated across Central Germany, Southern Germany, and Croatia (Supplementary Fig. 2 online). However, it can be predicted that more comprehensive sampling would produce an even more complex phylogeographic structure.

None of the Dinaric types were available for sequencing for the present study and most of them are lost in private collections that cannot be located or accessed. Fortunately, re-collections from several type localities were possible as part of this study, and established species hypotheses could be re-tested, thereby allowing us to comment on the present taxonomy. In Neobisiidae, the monophyly of the troglobiotic genus *Insulocreagris*, which exhibits a disjunct distribution on the island of Vis and the coastal area of southern Croatia, is highly supported in both the *COI* and concatenated datasets (see clade I, Fig. 3). Conversely, the genus *Protoneobisium*, known from two species on Biokovo Mountain, was neither supported genetically (6% interspecific *p*-distance to the closely related *Neobisium* species, see clade P, Fig. 3) or morphologically. The type species *Protoneobisium biocovense* nests within *Neobisium* and we transfer this species to the latter genus, resulting in the new combination *Neobisium biocovense* (Müller, 1931) comb. nov., and with *Protoneobisium* becoming a junior synonym of *Neobisium* (*Protoneobisium* = *Neobisium*, syn. nov.). Further, *Protoneobisium basilice* Ćurčić, Dimitrijević, Rađa and Rađa, 2008 becomes *Neobisium basilice* (Ćurčić, Dimitrijević, Rađa and Rađa, 2008) comb. nov. The type species of *Archaeoroncus*, *A. dalmatinus* (Hadži, 1933), nests within *Roncus* and we reclassify this species as *Roncus dalmatinus* (Hadži, 1933) comb. nov., thereby establishing the junior synonymy of *Archaeoroncus* under *Roncus* (*Archaeoroncus* = *Roncus*, syn. nov.). Further, *Archaeoroncus tenuis* (Hadži, 1933) becomes *Roncus tenuis* (Hadži, 1933) comb. nov., *Archaeoroncus salix* Ćurčić and Rađa, 2012 becomes *Roncus salix* (Ćurčić and Rađa, 2012) comb. nov., and *Archaeoroncus aspalathos* Ćurčić and Rađa, 2012 becomes *Roncus aspalathos* (Ćurčić and Rađa, 2012) comb. nov.

In the *COI* dataset of the family Chthoniidae, the troglobiotic genus *Troglochthonius* nested within a polytomy of other *Chthonius* species (see clade T in Fig. 3), while in the concatenated dataset it was placed as the most basal branch of Chthoniidae, and thus should be re-tested when additional data become available. The genera *Ephippiochthonius* Beier, 1930 and *Globochthonius*, that were elevated from subgeneric to generic rank by previous authors^34^, nested within *Chthonius* in the *COI* and concatenated datasets. The type species have type localities in Czech Republic and France and were not examined in this study. These genera are maintained for now but require further investigation.

In Chernetidae, both morphological examination and genetic analyses revealed three putative new species of the genus *Lasiochernes* that will be described elsewhere (see clade L in Fig. 3).

Although 17 *Roncus* species from Croatia are included in published identification keys^24,25^, many of these remain difficult to identify to the species level based on the available genetic data and morphology. Generally, the genus is characterized by several species-complexes and the diagnostic boundaries between species are often unclear^83^.

The Dinaric Karst of the Western Balkans is the global subterranean biodiversity hotspot^84^, nested within a key area for long-term conservation^85^ that support extraordinary radiations in many taxa^86^, relict species^9^, and short-range endemics. The importance of the area is often missed in biodiversity protection^87^. Results of the present study highlight the significant diversity of pseudoscorpions in the subterranean realm at both the generic and species level thereby warranting conservation management.

Populations of many morphospecies defined in this study represent evolutionarily significant units (ESUs)^88^ and were supported with different species delineation approaches, so we recognize the Dinaric Karst as a global hotspot for this ancient arachnid order. Of the 50 identified valid species and subspecies, 32 Dinaric Karst endemics (65%) merit priority conservation measures, both at the regional and national level. Subterranean habitats at Velebit and Biokovo Mountains, islands and areas around the alluvial plain in southern Croatia were identified as centers of taxonomic diversity and endemism and can be considered as “hotspots within hotspot”, hosting at least 18 Dinaric endemics of which all occur at small spatial scales (single-site endemics or species with ranges less than 10 km from the type locality). *COI* divergences in 15 cave-dwellers and 5 surface-dwellers indicated isolation of karstic populations and ongoing speciation that should be given the highest conservation priority.

Future species descriptions and assignment of species boundaries should provide rigorous and unbiased analysis of morphological, molecular, and ecological data, thereby accelerating conservation efforts in Croatia, which hosts 87 endemics^21^, including 61 (70%) single site-endemics and 20 (23%) with a linear distribution of less than 20 km^2^. Using both molecular and morphological data to document pseudoscorpion diversity in Croatia will also prevent an increase in taxonomic anarchy.

## Supplementary Information

Supplementary Information 1.

Supplementary Information 2.

Supplementary Information 3.

Supplementary Information 4.

Supplementary Information 5.

Supplementary Information 6.

## Data Availability

Specimens are deposited in the following collections: Croatian Biospeleological Society Collection (CBSS), Collection of Scorpiones and Pseudoscorpiones at the Croatian Natural History Museum (CNHM), and Collection at the Museum of Nature Hamburg - Zoology (formerly Zoological Museum Hamburg (ZMH)). Museum voucher numbers are listed in Supplementary Table 1 online, while sequences and metadata for specimens are publicly accessible in BOLD database under 10.5883/DS-CROPS and GenBank under the accession numbers ON841793–ON842291 for *COI* and ON950244–ON950333 for *28S*.
