# Supplementary figures and images for: Molecular analyses of pseudoscorpions in a subterranean biodiversity hotspot reveal cryptic diversity and microendemism
Source: Sci Rep. 2023 Jan 9;13:430. doi: 10.1038/s41598-022-26298-5 (PMC9829860; doi:10.1038/s41598-022-26298-5)

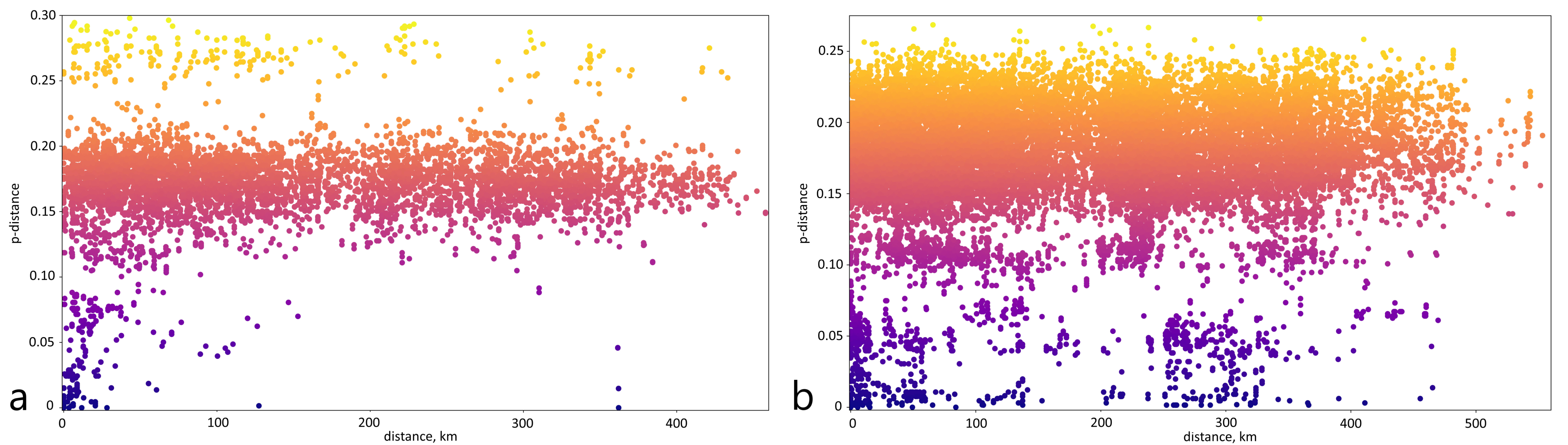

Supplement: Supplementary file 4 — Supplementary Information 4. [file 41598_2022_26298_MOESM4_ESM.tiff]

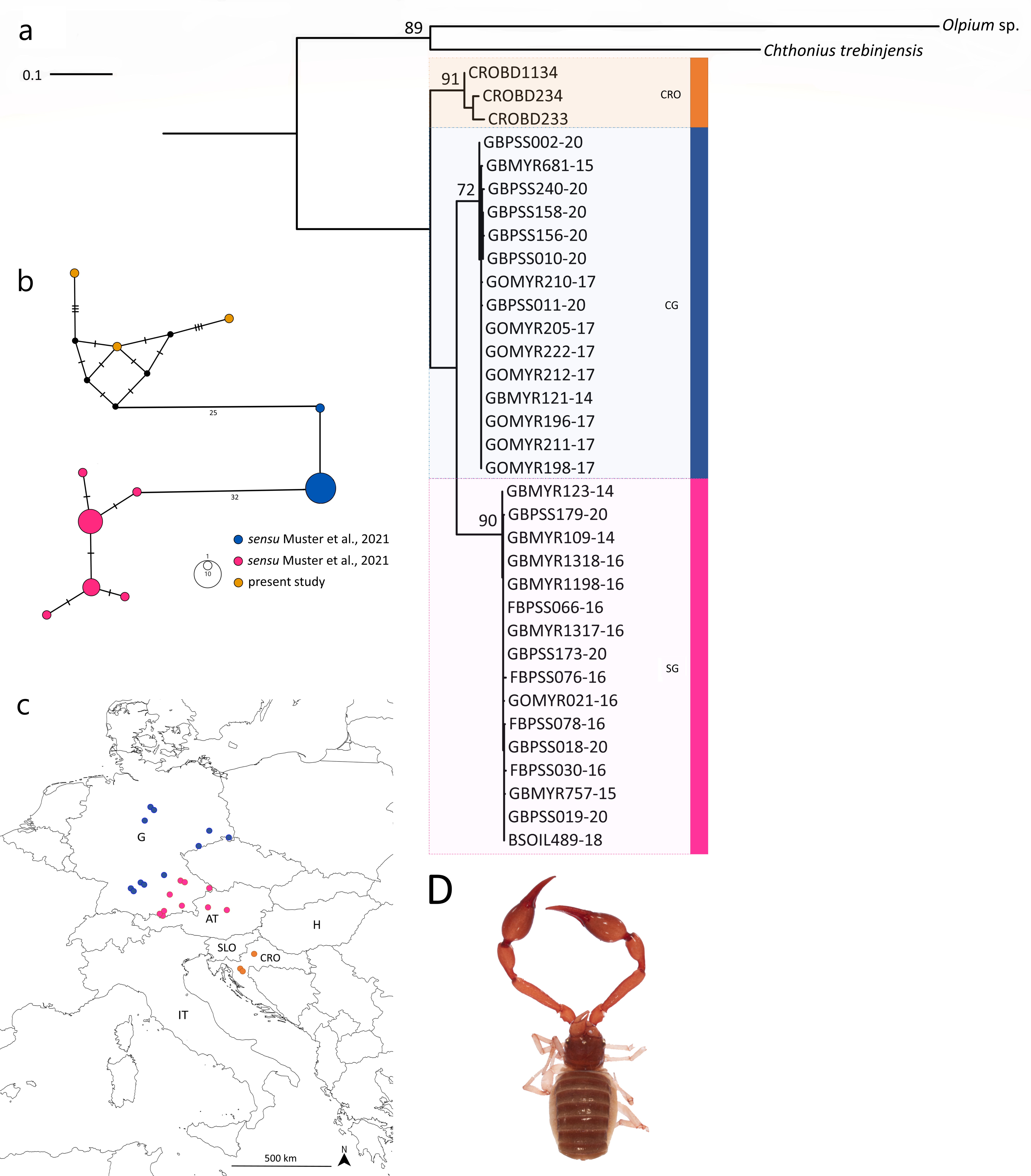

Supplement: Supplementary file 5 — Supplementary Information 5. [file 41598_2022_26298_MOESM5_ESM.tif]
