# Supplementary material for: Molecular analyses of pseudoscorpions in a subterranean biodiversity hotspot reveal cryptic diversity and microendemism
Source: Sci Rep. 2023 Jan 9;13:430. doi: 10.1038/s41598-022-26298-5 (PMC9829860; doi:10.1038/s41598-022-26298-5)
Supplement: Supplementary file 6 — Supplementary Information 6. [file 41598_2022_26298_MOESM6_ESM.pdf]

Supplementary Information for:

Molecular analyses of pseudoscorpions in a subterranean biodiversity hotspot reveal cryptic diversity and microendemism

Dora Hlebec, Martina Podnar, Mladen Kučinić, Danilo Harms

LIST OF SUPPLEMENTARY INFORMATION:

Supplementary Table 1: List of specimens sequenced in the present study

Supplementary Table 2: PCR protocols, thermocycling conditions, substitution models and partitions

Supplementary Table 3: Downloaded sequences from online repositories used in phylogenetic analyses

Supplementary Table 4: Results of BOLD's "Barcode Gap Analysis"

Supplementary Methods: Procedures of species delineation methods

Supplementary Table 5: Detailed results of applied species delineation methods for *COI* Dinaric dataset

Supplementary File 1: Preliminary phylogenetic analyses

Supplementary Figure 1: Representation of *COI* diversity at the spatial scale

Supplementary Figure 2: Phylogeographic structure of *Neobisium sylvaticum*

Alignment 1: *COI* sequences

Alignment 2: Concatenated *COI-28S* untrimmed alignment of Chthoniidae subset

Alignment 3: Concatenated *COI-28S* untrimmed alignment of Neobisiidae subset

Supplementary Table 1. List of DNA barcoded specimens. Species assignment after morphological examination, localities, sample IDs, BOLD IDs (*COI*)/collection inventory numbers (CINs), BINs, coordinates, and GenBank (*COI* and *28S*) accession numbers. Abbreviations: TL, type locality; CNHM, Croatian Natural History Museum; CBSS, Croatian Biospeleological Society; CRO = Croatia, MN = Montenegro, BH = Bosnia and Herzegovina.

Family, species, country, locality	Sample ID	BOLD ID/CIN	BIN	Longitude dec.	Latitude dec.	GenBank Accession Nos	
						COI	*28S*
Cheliferidae Risso, 1827							
1. *Rhacochelifer maculatus* (L. Koch, 1873)							
CRO: Jakišnica, Pag (under stones)	CROBD641	CROPS328-21/CNHM403	BOLD:AEO2699	44.3844	14.4637	ON841923	
CRO: Saline, Veliki Brijun (under stones)	CROBD646	CROPS331-21/CNHM189	BOLD:AEO2699	44.9081	13.7574	ON841947	
Chernetidae (Fabricius, 1793)							
2. *Chernes hahnii* (C.L. Koch, 1839)							
CRO: Nedelišće (tree bark)	CROBD468	CROPS300-21/CNHM576	BOLD:ADD3668	43.3773	16.3862	ON841883	
CRO: Gornji Kuršanec (tree bark)	CROBD594	CROPS316-21/CNHM594	BOLD:ADD3668	46.3299	16.3639	ON842289	
CRO: Gornji Kuršanec (tree bark)	CROBD595	CROPS317-21/CNHM595	BOLD:ADD3668	46.3299	16.3639	ON841848	
CRO: Čakovec, perivoj Zrinskih (tree bark)	CROBD632	CROPS421-22/CNHM601	BOLD:ADD3668	46.3884	16.4311	ON842179	
3. *Lasiochernes* sp. 1DH							
CRO: Čoćina jama, Nova sela, Metković	CROBD205	CROPS187-21/CNHM408	BOLD:AEI8484	43.1112	17.5668	ON841932	
CRO: Čoćina jama, Nova sela, Metković	CROBD290	CROPS248-21/CNHM491	BOLD:AEI8484	43.1112	17.5668	ON842134	
4. *Lasiochernes* sp. 2DH							
CRO: Vilina špilja, Gruda, Konavle	CROBD1019	CROPS390-22/CNHM778	BOLD:AEO5074	42.5066	18.3913	ON842122	
CRO: Vilina špilja, Gruda, Konavle	CROBD760	CROPS365-21/CNHM650	BOLD:AEO5074	42.5066	18.3913	ON841846	
5. *Lasiochernes* sp. 3DH							
CRO: Špilja kod Nerezinog dola, Goveđari, Mljet	CROBD1194	CROPS506-22/CBSS436I	BOLD:AEI8482	42.7584	17.4361	ON841949	
CRO: Špilja Bijelo jezero, Vukasi, Ponikve, Pelješac	CROBD269	CROPS236-21/CNHM470	BOLD:AEI8482	42.8469	17.6367	ON842238	
CRO: Čagjina jama, Orebić, Pelješac	CROBD583	CROPS067-21/CBSS32	BOLD:AEI8482	42.9771	17.1552	ON841917	
CRO: Špilja iznad Zamošća, Pelješac	CROBD586	CROPS070-21/CBSS35	BOLD:AEI8482	42.9786	17.1291	ON841987	
CRO: Jama više vodospreme, Draginac, Pelješac	CROBD588	CROPS072-21/CBSS37	BOLD:AEI8482	42.9794	17.296	ON842232	
CRO: Jama više vodospreme, Draginac, Pelješac	CROBD599	CROPS080-21/CBSS46	BOLD:AEI8482	42.9794	17.296	ON842212	
CRO: Jama više vodospreme, Draginac, Pelješac	CROBD607	CROPS086-21/CBSS54	BOLD:AEI8482	42.9794	17.296	ON842045	
CRO: Vranja jama, Vukova glavica, Ponikve, Ston, Pelješac	CROBD617	CROPS095-21/CBSS64	BOLD:AEI8482	42.8747	17.6165	ON842194	
CRO: Vilina špilja, izvor Omble, Dubrovnik	CROBD670	CROPS348-21/CNHM627	BOLD:AEI8482	42.6772	18.1362	ON842271	
CRO: Vilina špilja, izvor Omble, Dubrovnik	CROBD671	CROPS349-21/CNHM628	BOLD:AEI8482	42.6772	18.1362	ON842142	
CRO: Prnčeva špilja, Čućin vrh, Trstenik, Pelješac	CROBD767	CROPS371-21/CNHM657	BOLD:AEI8482	42.919	17.3869	ON842072	
CRO: Dočina špilja, Velika glavica, Blato, Mljet	CROBD1015	CROPS386-22/CNHM779	BOLD:AEI8482	42.7581	17.488	ON842083	

Chthoniidae Daday, 1889							
6. *Chthonius absoloni* Beier, 1938							
CRO: Gornja Baraćeva špilja, Nova Kršlja, Rakovica	CROBD1022	CROPS393-22/CBSS55A	BOLD:AEI9711	44.9839	15.7217	ON842139	
CRO: Gornja Baraćeva špilja, Nova Kršlja, Rakovica	CROBD1023	CROPS394-22/CBSS55B	BOLD:AEI9711	44.9839	15.7217	ON842200	
CRO: Gornja Baraćeva špilja, Nova Kršlja, Rakovica	CROBD682	CROPS104-21/CBSS11B	BOLD:AEI9711	44.9839	15.7217	ON842040	
CRO: Gornja Baraćeva špilja, Nova Kršlja, Rakovica	CROBD683	CROPS105-21/CBSS12B	BOLD:AEI9711	44.9839	15.7217	ON842147	
CRO: Gornja Baraćeva špilja, Nova Kršlja, Rakovica	CROBD684	CROPS106-21/CBSS13B	BOLD:AEI9711	44.9839	15.7217	ON842079	
CRO: Gornja Baraćeva špilja, Nova Kršlja, Rakovica	CROBD685	CROPS107-21/CBSS14B	BOLD:AEI9711	44.9839	15.7217	ON841926	
CRO: Gornja Baraćeva špilja, Nova Kršlja, Rakovica	CROBD686	CROPS422-22/CBSS15B	BOLD:AEI9711	44.9839	15.7217	ON842184	
CRO: Donja Baraćeva špilja, Nova Kršlja, Rakovica	CROBD690	CROPS111-21/CBSS20B	BOLD:AEI9710	44.984	15.7227	ON842220	
CRO: Gornja Baraćeva špilja, Nova Kršlja, Rakovica	CROBD694	CROPS423-22/CBSS24B	BOLD:AEI9711	44.9839	15.7217	ON841936	
CRO: Donja Baraćeva špilja, Nova Kršlja, Rakovica	CROBD697	CROPS116-21/CBSS29	BOLD:AEI9711	44.984	15.7227	ON842178	
CRO: Gornja Baraćeva špilja, Nova Kršlja, Rakovica	CROBD698	CROPS117-21/CBSS30	BOLD:AEI9711	44.9839	15.7217	ON842138	
CRO: Gornja Baraćeva špilja, Nova Kršlja, Rakovica	CROBD699	CROPS118-21/CBSS31	BOLD:AEI9710	44.9839	15.7217	ON841800	
MN: Pećina Golodražnica, Risan	CROBD705	CROPS424-22/CNHM637	BOLD:AEI9711	42.503	18.699	ON842097	
MN: Pećina Golodražnica, Risan	CROBD706	CROPS425-22/CNHM638	BOLD:AEI9711	42.503	18.699	ON842222	
CRO: Donja Baraćeva špilja, Nova Kršlja, Rakovica	CROBD742	CROPS123-21/CBSS40B	BOLD:AEI9710	44.984	15.7227	ON842196	
7. *Chthonius* aff. *absoloni* Beier, 1938							
CRO: Špilja u Privoru, Podkalače, Donja Vrućica, Pelješac	CROBD258	CROPS228-21/CNHM459	BOLD:AEO5570	43.0092	17.2133	ON841894	
CRO: Jama Golubinica, Donja Banda, Pelješac	CROBD262	CROPS232-21/CNHM463	BOLD:AEI7334	42.971	17.2872	ON842190	
CRO: Jama Golubinica, Donja Banda, Pelješac	CROBD263	CROPS233-21/CNHM464	BOLD:AEI7334	42.971	17.2872	ON842050	
CRO: Špilja Crno jezero, Vukasi, Ponikve, Ston, Pelješac	CROBD568	CROPS054-21/CBSS17	BOLD:AEI2657	42.8473	17.6364	ON842280	
CRO: Špilja Crno jezero, Vukasi, Ponikve, Ston, Pelješac	CROBD570	CROPS056-21/CBSS19	BOLD:AEI2657	42.8473	17.6364	ON841864	
CRO: Špilja na Grivinom brijegu, Dančanje, Ponikve	CROBD587	CROPS071-21/CBSS36	BOLD:AEI9067	42.8794	17.5804	ON842290	
CRO: Špilja kod Zagruda u vinogradu	CROBD618	CROPS096-21/CBSS65	BOLD:AEI7334	42.9666	17.3227	ON842230	
8. *Chthonius* aff. *alpicola* Beier, 1951							
CRO: Golubinka u gaju, Baletići, Zavojane, Vrgorac	CROBD241	CROPS212-21/CNHM442	BOLD:AEO0176	43.2552	17.2659	ON841934	
CRO: Golubinka u gaju, Baletići, Zavojane, Vrgorac	CROBD243	CROPS214-21/CNHM444	BOLD:AEO0176	43.2552	17.2659	ON842034	
CRO: Jujnovića špilja (Špilja Gradina)	CROBD283	CROPS245-21/CNHM484	BOLD:AEO3625	43.2648	17.1993	ON842073	
9. *Chthonius* aff. *exarmatus* Beier, 1939							
CRO: Dočina špilja, Velika glavica, Blato, Mljet	CROBD1016	CROPS387-22/CNHM780	BOLD:AEO3626	42.7581	17.488	ON841943	ON950244
CRO: Dočina špilja, Velika glavica, Blato, Mljet	CROBD1017	CROPS388-22/CNHM781	BOLD:AEO3626	42.7581	17.488	ON842071	
10. *Chthonius* aff. *heterodactylus* (Tömösváry, 1883)							
CRO: Rudnik sv. Barbare, Rude (under stones)	CROBD643	CROPS329-21/CNHM361	BOLD:AEO4839	45.7631	15.6715	ON842108	

11. *Chthonius* aff. *ischnocheles* (Hermann, 1804)							
CRO: Špilja na gradini kod Premanture, Medulin, Istra	CROBD1154	CROPS472-22/CBSS418I	BOLD:AEO3709	44.7681	13.9164	ON841955	
CRO: Špilja na gradini kod Premanture, Medulin, Istra	CROBD1155	CROPS473-22/CBSS421I	BOLD:AEO3709	44.7681	13.9164	ON841999	ON950249
12. *Chthonius* aff. *occultus* Beier, 1939							
CRO: Jama na Pudarnici, Čeveljuša, Ploče	CROBD1081	CROPS395-22/CNHM782	BOLD:AEO5573	43.0542	17.467	ON842104	
CRO: Mandina jama, Stanine, Vid, Metković	CROBD220	CROPS197-21/CNHM421	BOLD:AEI7848	43.0831	17.6063	ON842043	
CRO: Mandina jama, Stanine, Vid, Metković	CROBD221	CROPS198-21/CNHM422	BOLD:AEI7848	43.0831	17.6063	ON842064	
CRO: Mandina jama, Stanine, Vid, Metković	CROBD222	CROPS199-21/CNHM423	BOLD:AEI7848	43.0831	17.6063	ON841837	
CRO: Mandina jama, Stanine, Vid, Metković	CROBD223	CROPS200-21/CNHM424	BOLD:AEI7848	43.0831	17.6063	ON841901	
CRO: Mandina jama, Stanine, Vid, Metković	CROBD224	CROPS201-21/CNHM425	BOLD:AEI7848	43.0831	17.6063	ON842183	
CRO: Jama malog ulaza, Zamagorje, Zavojane, Vrgorac	CROBD247	CROPS218-21/CNHM448	BOLD:AEI2140	43.2515	17.2709	ON841833	
CRO: Jama malog ulaza, Zamagorje, Zavojane, Vrgorac	CROBD249	CROPS220-21/CNHM450	BOLD:AEI2140	43.2515	17.2709	ON841914	ON950266
CRO: Jama malog ulaza, Zamagorje, Zavojane, Vrgorac	CROBD250	CROPS221-21/CNHM451	BOLD:AEI2140	43.2515	17.2709	ON842218	
CRO: Jama malog ulaza, Zamagorje, Zavojane, Vrgorac	CROBD252	CROPS223-21/CNHM453	BOLD:AEI2140	43.2515	17.2709	ON842186	
CRO: Jama malog ulaza, Zamagorje, Zavojane, Vrgorac	CROBD253	CROPS224-21/CNHM454	BOLD:AEI2140	43.2515	17.2709	ON842048	
CRO: Jama malog ulaza, Zamagorje, Zavojane, Vrgorac	CROBD254	CROPS225-21/CNHM455	BOLD:AEI2140	43.2515	17.2709	ON842070	
CRO: Mandina jama, Stanine, Vid, Metković	CROBD268	CROPS235-21/CNHM469	BOLD:AEI7848	43.083	17.606	ON842067	
CRO: Jamica u docima, Sv. Roko, Desne, Metković	CROBD284	CROPS246-21/CNHM485	BOLD:AEO1301	43.067	15.533	ON842074	
CRO: Jama pod Kamenitim vratima, Biokovo	CROBD384	CROPS002-21/CNHM783	BOLD:AEI2135	43.3008	17.1193	ON842103	
CRO: Baba špilja, Biokovo	CROBD398	CROPS012-21/CNHM784	BOLD:AEI2660	43.2565	17.1623	ON842233	
CRO: Velika špilja kod Antunovića, Biokovo	CROBD400	CROPS014-21/CNHM785	BOLD:AEI2140	43.2768	17.2271	ON841997	
CRO: Spila 2, Jujnovići Kozice, Biokovo	CROBD655	CROPS334-21/CNHM612	BOLD:AEI2140	43.2624	17.204	ON841989	
CRO: Jama pod Gažnovcem, Stilija, Vrgorac	CROBD661	CROPS340-21/CNHM618	BOLD:AEI2140	43.2297	17.3142	ON842086	
CRO: Jama pod Gažnovcem, Stilija, Vrgorac	CROBD662	CROPS341-21/CNHM618	BOLD:AEI2140	43.2297	17.3142	ON842035	
CRO: Čađina golubinka, Eraci, Ploče	CROBD772	CROPS375-21/CNHM662	BOLD:AEO5573	43.1042	17.4931	ON842253	
CRO: Čađina golubinka, Eraci, Ploče	CROBD773	CROPS376-21/CNHM663	BOLD:AEO5573	43.1042	17.4931	ON842000	
CRO: Njemica, Bukov vrh, Biokovo	CROBD900	CROPS447-22/CNHM676	BOLD:AEI2135	43.3083	17.0778	ON842106	
CRO: Njemica, Bukov vrh, Biokovo	CROBD905	CROPS449-22/CBSS184I	BOLD:AEI2135	43.3083	17.0778	ON842054	ON950332
13. *Chthonius* aff. *radjai* Ćurčić, 1988							
CRO: Špilja na Budimu, NP Sjeverni Velebit (<2 km from TL)	CROBD564	CROPS050-21/CBSS13	BOLD:AEI7341	44.7974	14.9417	ON842167	
CRO: Kalvarija, Bužim, Gospić (<2 km from TL)	CROBD863	CROPS162-21/CBSS150	BOLD:AEI4085	44.5856	15.2448	ON841938	
14. *Chthonius* aff. *subterraneus* Beier, 1931							
CRO: Kozjakuša jama, Vršeljci, Velebit (<2 km from TL)	CROBD800	CROPS428-22/CBSS87I	BOLD:AEO4469	44.6753	15.109	ON841911	
15. *Chthonius exarmatus* Beier, 1939							
CRO: Tomasova spilja, Duba pelješka, Pelješac	CROBD272	CROPS238-21/CNHM473	BOLD:AEI7340	43.0142	17.1655	ON841959	
CRO: Poskokova jama, Sv. Ilija, Pelješac	CROBD558	CROPS044-21/CBSS7	BOLD:AEI7340	43.0021	17.0912	ON841803	
CRO: Poskokova jama, Sv. Ilija, Pelješac	CROBD559	CROPS045-21/CBSS8	BOLD:AEI7340	43.0021	17.0912	ON842241	

CRO: Poskokova jama, Sv. Ilija, Orebić, Pelješac	CROBD765	CROPS426-22/CNHM655	BOLD:AEI7340	43.0021	17.0912	ON842016	ON950314
16. *Chthonius ischnocheles* (Hermann, 1804)							
CRO: Biokovo, Grebišće (under stones)	CROBD358	CROPS285-21/CNHM559	BOLD:AEO1297	43.3474	16.9797	ON842133	ON950276
CRO: Postira, Brač (under stones)	CROBD635	CROPS323-21/CNHM604	BOLD:AEO5572	43.3763	16.638	ON842049	ON950307
CRO: Postira, Brač (under stones)	CROBD637	CROPS325-21/CNHM606	BOLD:AEO5572	43.3763	16.638	ON842216	
CRO: Postira, Brač (under stones)	CROBD638	CROPS326-21/CNHM607	BOLD:AEO5572	43.3763	16.638	ON841852	ON950309
17. *Chthonius ischnocheles ischnocheles* (Hermann, 1804)							
CRO: Zelena jama, Kornat, NP Kornati	CROBD1110	CROPS407-22/CBSS13I	BOLD:AEO2197	43.8326	15.2909	ON841928	
CRO: Jama iznad Špraljin stana, Kornat, NP Kornati	CROBD1114	CROPS409-22/CBSS29I	BOLD:AEO5571	43.8726	15.23	ON842171	
18. *Chthonius ischnocheles reductus* Beier, 1939							
CRO: Činjadra, Nerežišća, Brač	CROBD1109	CROPS406-22/CBSS8I	BOLD:AEO3284	43.3398	16.5995	ON842235	
19. *Chthonius magnificus* Beier, 1938							
CRO: Šipun, Cavtat (TL)	CROBD1116	CROPS410-22/CBSS37I	BOLD:AEI7337	42.5836	18.218	ON842033	
CRO: Šipun, Cavtat (TL)	CROBD1120	CROPS412-22/CBSS114I	BOLD:AEI7337	42.5836	18.218	ON841958	
CRO: Bezdan, Vignje, Čilipi	CROBD1124	CROPS416-22/CBSS138I	BOLD:AEI2138	42.5304	18.3163	ON842111	
CRO: Vilina špilja, Gruda, Konavle	CROBD1152	CROPS471-22/CBSS416I	BOLD:AEO0175	42.6772	18.1362	ON842041	ON950248
CRO: Đurovića špilja, Cavtat	CROBD1165	CROPS483-22/CBSS166I	BOLD:AEO5530	42.56	18.2636	ON842187	ON950255
BH: Baba, Čvaljina, Popovo polje	CROBD1182	CROPS496-22/CBSS149I	BOLD:AEI2138	42.87	17.97	ON842173	ON950260
CRO: Bezdan, Vignje, Čilipi	CROBD1190	CROPS503-22/CBSS152I	BOLD:AEI2138	42.5304	18.3163	ON842029	ON950261
CRO: Bezdan, Vignje, Čilipi	CROBD412	CROPS023-21/CNHM786	BOLD:AEI2138	42.5304	18.3163	ON841957	ON950291
CRO: Špilja od Punta	CROBD432	CROPS033-21/CNHM787	BOLD:AEI2137	42.5561	18.2433	ON842144	ON950296
CRO: Vilina špilja, Gruda, Konavle	CROBD761	CROPS366-21/CNHM651	BOLD:AEO0175	42.5067	18.3913	ON842291	
CRO: Vilina špilja, Gruda, Konavle	CROBD763	CROPS368-21/CNHM653	BOLD:AEO0175	42.5067	18.3913	ON841884	
CRO: Šipun, Cavtat (TL)	CROBD838	CROPS154-21/CBSS125	BOLD:AEI7337	42.5836	18.218	ON842282	
20. *Chthonius occultus* Beier, 1939							
CRO: Špilja za Gromačkom vlakom, Gromača, Dubrovnik	CROBD1178	CROPS493-22/CBSS181I	BOLD:AEI7852	42.7435	18.0274	ON842026	
CRO: Bezdan, Vignje, Čilipi	CROBD1189	CROPS502-22/CBSS190I	BOLD:AEO1300	42.5304	18.3163	ON842195	
BH: Žira, Popovo polje, Dobri Do, Hutovo (<2 km from TL)	CROBD282	CROPS244-21/CNHM483	BOLD:AEO1300	42.91	17.84	ON842153	
CRO: Jama za Rasohama	CROBD416	CROPS024-21/CNHM788	BOLD:AEI2659	42.8024	17.9282	ON842159	
CRO: Krivača špilja, Osojnik, Dubrovnik	CROBD420	CROPS028-21/CNHM789	BOLD:AEI7852	42.7302	18.0676	ON841994	ON950294
CRO: Špilja za Gromačkom vlakom, Gromača, Dubrovnik	CROBD436	CROPS035-21/CNHM790	BOLD:AEI7852	42.7435	18.0274	ON841815	
CRO: Špilja za Gromačkom vlakom, Gromača, Dubrovnik	CROBD869	CROPS165-21/CBSS156	BOLD:AEI7852	42.7435	18.0274	ON841859	

21. *Chthonius raridentatus* Hadži, 1930							
CRO: Židovske kuće, Cerovica	CROBD1102	CROPS399-22/CBSS4BI	BOLD:AEO4467	45.8095	15.4764	ON842125	
CRO: Dolača, Drašči vrh, Žumberak	CROBD1104	CROPS401-22/CBSS6BI	BOLD:AEO4467	45.7396	15.4828	ON841820	
CRO: Veternica, Zagreb (TL of *C. jalzici*)	CROBD1149	CROPS469-22/CBSS486I	BOLD:AEO4467	45.8411	15.8736	ON842202	ON950246
22. *Chthonius* sp. 1DH							
CRO: Jama u Šipku, Šipar, Baćina, Ploče	CROBD225	CROPS202-21/CNHM426	BOLD:AEO1683	43.084	17.387	ON842263	ON950264
23. *Chthonius* sp. 2DH							
CRO: Golubinka u gaju, Baletići, Zavojane, Vrgorac	CROBD244	CROPS215-21/CNHM445	BOLD:AEO0564	43.2552	17.2659	ON842199	
24. *Chthonius* sp. LI							
CRO: Kalinovača, Gospić (under stones)	CROBD1151	CROPS470-22/CNHM791	BOLD:AEO4468	44.6308	15.1994	ON842129	ON950246
25. *Chthonius* sp. LK							
CRO: Donja Baraćeva špilja, Nova Kršlja, Rakovica	CROBD680	CROPS102-21/CBSS9B	BOLD:AEI7850	44.984	15.7227	ON841972	
26. *Chthonius* sp. nov. D							
CRO: Špilja za Gromačkom vlakom, Gromača, Dubrovnik	CROBD868	CROPS164-21/CBSS155	BOLD:AEI3631	42.7435	18.0274	ON842063	ON950327
27. *Chthonius* sp. nov. K							
CRO: Velika pećina, Korana canyon	CROBD1156	CROPS474-22/CBSS471I	BOLD:AEN9341	44.9112	15.6069	ON842278	ON950250
CRO. Ralićeva špilja, Lipovac, Rakovica	CROBD321	CROPS262-21/CNHM522	BOLD:AEN9341	45.0191	15.6712	ON841990	
CRO: Dujićeva pećina, Jezerce, Plitvička jezera	CROBD527	CROPS307-21/CNHM585	BOLD:AEN9341	44.8567	15.6787	ON842038	
28. *Chthonius* sp. nov. KO							
CRO: Vilina špilja, izvor Omble, Dubrovnik	CROBD669	CROPS347-21/CNHM626	BOLD:AEO5528	42.6772	18.1362	ON842201	
29. *Chthonius* sp. nov. L							
CRO: Dumenčića špilja, Keseri, Stara Kršlja, Rakovica	CROBD679	CROPS101-21/CBSS8B	BOLD:AEI7335	45.0247	15.6692	ON842148	
CRO: Đutno, Liplje, Zdihovo	CROBD865	CROPS435-22/CBSS152	BOLD:AEI7335	45.4156	15.2331	ON842019	
30. *Chthonius* sp. nov. M1							
CRO: Galičnjak, Okuklje, Mljet	CROBD1013	CROPS385-22/CNHM792	BOLD:AEO3283	42.7202	17.6707	ON842285	
CRO: Galičnjak, Okuklje, Mljet	CROBD1012	CROPS384-22/CNHM793	BOLD:AEO3283	42.7202	17.6707	ON842197	
31. *Chthonius* sp. nov. M2							
CRO: Mala špilja, Blato, Mljet	CROBD310	CROPS258-21/CNHM511	BOLD:AEO0566	42.7645	17.4711	ON841893	ON950269
CRO: Male ponte, Goveđari, Mljet	CROBD315	CROPS260-21/CNHM516	BOLD:AEO5527	42.7614	17.4308	ON842096	
32. *Chthonius* sp. nov. P							
CRO: Kameni kolačić	CROBD604	CROPS084-21/CBSS51	BOLD:AEI9068	42.8731	17.4664	ON842061	
33. *Chthonius* sp. nov. PL							
CRO: Kaluđerova pećina, Veliki slap, Plitvice selo	CROBD1161	CROPS479-22/CBSS476I	BOLD:AEN9657	44.9018	15.6093	ON842056	
CRO: Kaluđerova pećina, Veliki slap, Plitvice selo	CROBD1162	CROPS480-22/CBSS477I	BOLD:AEN9657	44.9018	15.6093	ON841855	ON950253
34. *Chthonius* sp. nov. PLJ							
CRO: Na-Kovana jama, Orebić, Pelješac	CROBD577	CROPS062-21/CBSS26	BOLD:AEI2136	42.9985	17.0877	ON842055	
CRO: Na-Kovana jama, Orebić, Pelješac	CROBD611	CROPS090-21/CBSS58	BOLD:AEI2136	42.9985	17.0877	ON842255	ON950305

CRO: Poskokova jama, Sv. Ilija, Orebić, Pelješac	CROBD764	CROPS369-21/CNHM654	BOLD:AEO5526	43.0021	17.0912	ON842284	
CRO: Na-Kovana jama, Nakovane, Orebić	CROBD778	CROPS379-21/CNHM668	BOLD:AEI2136	42.9985	17.0877	ON842149	
35. *Chthonius* sp. nov. R							
CRO: Jama iznad Šnjatog dola, Šnjati dol, Raba	CROBD806	CROPS429-22/CBSS93	BOLD:AEO0177	42.9829	17.5528	ON842053	ON950318
36. *Chthonius* sp. nov. V							
CRO: Terca, Golubić, NP Sjeverni Velebit	CROBD563	CROPS049-21/CBSS12	BOLD:AEI2134	44.7617	15.0433	ON842242	
37. *Chthonius* sp. nov. VL							
CRO: Manita peć, Starigrad Paklenica, NP Paklenica	CROBD368	CROPS290-21/CBSS1N	BOLD:AEI7343	44.3158	15.4763	ON841831	
CRO: Špiljica u šumi Badnja, Struge, NP Paklenica	CROBD921	CROPS180-21/CBSS200	BOLD:AEI7343	44.3798	15.4656	ON842281	
CRO: Vaganka, Veliki Vaganac, Starigrad Paklenica	CROBD922	CROPS181-21/CBSS201	BOLD:AEI7343	44.3384	15.4387	ON841862	ON950333
38. *Chthonius* sp. ZB							
CRO: Bedara, Tihočaj, Žumberak (under stones)	CROBD1105	CROPS402-22/CBSS7BI	BOLD:AEO4468	45.7519	15.5367	ON842192	
CRO: Bedara, Tihočaj, Žumberak (under stones)	CROBD1113	CROPS408-22/CBSS24I	BOLD:AEO1682	45.7519	15.5367	ON841856	
39. *Chthonius subterraneus* Beier, 1931							
CRO: Jama Magazin, Čorkova uvala, NP Plitvička jezera	CROBD1158	CROPS476-22/CBSS473I	BOLD:AEO2148	44.8906	15.5511	ON842012	ON950251
40. *Chthonius subterraneus meuseli* Beier, 1939							
CRO: Dumenčića špilja, Keseri, Stara Kršlja, Rakovica (<2 km from TL)	CROBD688	CROPS109-21/CBSS18B	BOLD:AEI2663	45.0247	15.6692	ON842175	
CRO: Dumenčića špilja, Keseri, Stara Kršlja, Rakovica (<2 km from TL)	CROBD696	CROPS115-21/CBSS28B	BOLD:AEI2663	45.0247	15.6692	ON841798	
CRO: Baraćeve špilje, Nova Kršlja, Rakovica (under stones) (<2 km from TL)	CROBD740	CROPS121-21/CBSS36B	BOLD:AEI2663	44.984	15.7227	ON841937	
CRO: Baraćeve špilje, Nova Kršlja, Rakovica (under stones) (<2 km from TL)	CROBD741	CROPS122-21/CBSS37B	BOLD:AEI2663	44.984	15.7227	ON841869	
CRO: Dumenčića špilja, Keseri, Stara Kršlja, Rakovica (<2 km from TL)	CROBD743	CROPS124-21/CBSS41B	BOLD:AEI2663	45.0247	15.6692	ON841811	
41. *Chthonius subterraneus subterraneus* Beier, 1931							
CRO: Jezero, Snježnica, Konavle	CROBD417	CROPS025-21/CNHM794	BOLD:AEI2662	42.5804	18.3561	ON842052	
CRO: Jezero, Snježnica, Konavle	CROBD418	CROPS026-21/CNHM795	BOLD:AEI2662	42.5804	18.3561	ON841847	
MN: Vilina pećina, Podi, Crkvice	CROBD862	CROPS161-21/CBSS149	BOLD:AEI7851	42.5546	18.6624	ON842145	ON950326
42. *Chthonius trebinjensis* Beier, 1938							
CRO: Močiljska špilja, Osojnik, Dubrovnik	CROBD1180	CROPS494-22/CNHM796	BOLD:AEO4975	42.6899	18.071	ON842172	ON950258
CRO: Pećina u gaju, Osojnik, Dubrovnik	CROBD327	CROPS265-21/CNHM528	BOLD:AEO1296	42.7045	18.0828	ON842229	
CRO: Špilja pod Krstom	CROBD435	CROPS034-21/CNHM797	BOLD:AEO4976	42.6777	18.0619	ON841802	ON950297
CRO: Vrbočulje, Zaton, Dubrovnik	CROBD444	CROPS040-21/CNHM798	BOLD:AEI2142	42.6931	18.0316	ON842113	ON950300
CRO: Močiljska špilja, Osojnik, Dubrovnik	CROBD653	CROPS332-21/CNHM610	BOLD:AEO4975	42.6899	18.071	ON842264	
BH: Vučja pećina, Popovo polje, Trebinje (<2 km from TL)	CROBD876	CROPS439-22/CBSS163	BOLD:AEO1681	42.716	18.326	ON842101	ON950329
43. *Ephippiochthonius* aff. *insularis* (Beier, 1938)							
CRO: Prnčeva špilja, Čućin vrh, Trstenik, Pelješac	CROBD768	CROPS372-21/CNHM658	BOLD:AEO5574	42.919	17.3869	ON841988	ON950315

44. *Ephippiochthonius tetrachelatus* (Preyssler, 1790)								
CRO: Biokovo, Kotišina (under stones)	CROBD353	CROPS282-21/CNHM554	BOLD:AEO0173	43.291	17.048	ON842009	ON950274
CRO: Biokovo, Župa (under stones)	CROBD354	CROPS283-21/CNHM555	BOLD:AEO0174	43.322	17.1351	ON842240	
CRO: Biokovo (under stones)	CROBD355	CROPS284-21/CNHM556	BOLD:AEO1299	43.3474	16.9797	ON841963	ON950275
CRO: Biokovo, Grebišće (under stones)	CROBD359	CROPS286-21/CNHM560	BOLD:AEO1302	43.3474	16.9797	ON842228	
CRO: Biokovo, Grebišće (under stones)	CROBD362	CROPS287-21/CNHM563	BOLD:AEO1302	43.3474	16.9797	ON842259	
CRO: Biokovo, northern slope (under stones)	CROBD364	CROPS288-21/CNHM565	BOLD:AEO1302	43.2917	17.0559	ON842226	
CRO: Biokovo, northern slope (under stones)	CROBD365	CROPS289-21/CNHM566	BOLD:AEO1302	43.2917	17.0559	ON841978	
CRO: Ostojića golubinka, Gradci, Peračko blato, Ploče	CROBD885	CROPS442-22/CBSS172	BOLD:AEO0565	43.0995	17.4297	ON841807	ON950330
45. *Ephippiochthonius insularis* (Beier, 1938)							
CRO: Movrica, Babino polje, Mljet (TL)	CROBD1186	CROPS499-22/CBSS449I	BOLD:AEO5529	42.7525	17.5512	ON842262	
CRO: Jama na Strmici, Kozarica, Mljet (<2 km from TL)	CROBD298	CROPS251-21/CNHM499	BOLD:AEO1298	42.7671	17.4535	ON841858	
CRO: Jama na Strmici, Kozarica, Mljet (<2 km from TL)	CROBD300	CROPS252-21/CNHM501	BOLD:AEO1298	42.7671	17.4535	ON842287	ON950267
CRO: Močiljska špilja, Osojnik, Dubrovnik	CROBD423	CROPS029-21/CNHM799	BOLD:AEI2661	42.6899	18.071	ON842123	
CRO: Špilja u Žarmi, Zamošće, Pelješac	CROBD557	CROPS043-21/CBSS6	BOLD:AEI7339	42.9354	17.4382	ON842154	
46. *Ephippiochthonius* sp. 1DH							
CRO: Velika pećina (Velika špilja), Plančica, NP S. Velebit	CROBD555	CROPS042-21/CBSS4	BOLD:AEI7338	44.7758	14.9397	ON842107	ON950302
47. *Globochthonius* aff. *caligatus* (Beier, 1938)							
CRO: Ivanova jama, Crni kuk, Mala žaba, Dobranje	CROBD834	CROPS151-21/CBSS121	BOLD:AEI7854	42.9734	17.7008	ON842105	
48. *Globochthonius caligatus* (Beier, 1938)							
CRO: Vilina špilja, Gruda, Konavle	CROBD1020	CROPS391-22/CNHM800	BOLD:AEO3814	42.6772	18.1362	ON841966	
CRO: Golubljenica, Duba Konavoska, Snježnica	CROBD259	CROPS229-21/CNHM460	BOLD:AEI7853	42.6076	18.3269	ON841813	
CRO: Miljkovića staje, Špilja kod Miljkovića staja, Slano	CROBD437	CROPS036-21/CNHM801	BOLD:AEI2658	42.8992	17.8039	ON842198	
CRO: Vilina špilja, Gruda, Konavle	CROBD759	CROPS364-21/CNHM649	BOLD:AEO3814	42.5067	18.3913	ON841979	
BH: Pavlova pećina, Bihovo, Trebinje (<2 km from TL)	CROBD807	CROPS135-21/CBSS94	BOLD:AEI7853	42.6664	18.3077	ON841912	ON950319
BH: Pavlova pećina, Bihovo, Trebinje (<2 km from TL)	CROBD841	CROPS433-22/CBSS128	BOLD:AEI7853	42.6664	18.3077	ON842084	ON950325
BH: Pavlova pećina, Bihovo, Trebinje (<2 km from TL)	CROBD866	CROPS436-22/CBSS153	BOLD:AEI7853	42.6664	18.3077	ON841851	
49. *Globochthonius* sp. n. B							
CRO: Led 1 jama, Biokovo	CROBD386	CROPS004-21/CNHM802	BOLD:AEI2133	43.344	17.0576	ON841956	
CRO: Kukor (Špilja u Bastu)	CROBD396	CROPS010-21/CNHM803	BOLD:AEI2664	43.3554	16.9913	ON842208	
CRO: Kukor (Špilja u Bastu)	CROBD909	CROPS450-22/CBSS188	BOLD:AEO1684	43.3554	16.9913	ON841805	
50. *Globochthonius* sp. n. D							
CRO: Drinova 2	CROBD403	CROPS017-21/CNHM804	BOLD:AEI2139	43.4077	16.9429	ON841892	
CRO: Drinova 2	CROBD672	CROPS350-21/CNHM629	BOLD:AEI2139	43.4077	16.9429	ON842221	
CRO: Drinova 2	CROBD673	CROPS351-21/CNHM630	BOLD:AEI2139	43.4077	16.9429	ON841918	
CRO: Vranjača, Kotlenice, Dugopolje, Mosor	CROBD1166	CROPS484-22/CBSS106I	BOLD:AEO5531	43.5622	16.6482	ON841871	

51. *Globochthonius* sp. n. D							
CRO: Podslotinjak, Žman, Dugi	CROBD813	CROPS137-21/CBSS100	BOLD:AEI7336	43.9577	15.1175	ON842015	
52. *Globochthonius* sp. n. S							
CRO: Peć u Čulinovim raljevinama, Kolić, Dugopolje, Split	CROBD875	CROPS438-22/CBSS162	BOLD:AEO5568	43.6059	16.5751	ON841888	
53. *Microchthonius elegantissimus* Ćurčić, Rađa, S.B. Ćurčić, Ilić, Tomić and Makarov, 2013							
CRO: Jama 7, ŽC GO, Lećevica, Kladnjice, Split (<2 km from TL)	CROBD907	CROPS176-21/CBSS186I	BOLD:AEI7849	43.6918	16.2915	ON842121	
54. *Occidenchthonius* sp. PEL							
CRO: Prnčeva špilja, Čućin vrh, Trstenik, Pelješac	CROBD766	CROPS370-21/CNHM656	BOLD:AEO5569	42.919	17.3869	ON842206	
55. *Troglochthonius* aff. *mirabilis* Beier, 1939							
CRO: Jama za Supinom, Biokovo	CROBD402	CROPS016-21/CNHM805	BOLD:AEI4294	43.2505	17.1133	ON842098	ON950285
56. *Troglochthonius mirabilis* Beier, 1939							
CRO: Bezdan, Vignje, Čilipi	CROBD411	CROPS022-21/CNHM806	BOLD:AEI4295	42.5304	18.3163	ON841984	ON950290
Garypidae Simon, 1879							
57. *Garypus* sp. nov. M							
CRO: Malo jezero, Mljet (under stones)	CROBD519	CROPS301-21/CNHM577	BOLD:AEO5084	42.7792	17.3456	ON842135	
Neobisiidae Chamberlin, 1930							
58. *Insulocreagris* sp. 1DH							
CRO: Rudnik Kmegla, Bardorovica, Vis	CROBD204	CROPS186-21/CNHM407	BOLD:AEI0671	43.0579	16.1466	ON841897	
CRO: Rudnik Kmegla, Bardorovica, Vis	CROBD521	CROPS302-21/CNHM579	BOLD:AEI0671	43.0579	16.1466	ON841844	
59. *Insulocreagris* sp. 2DH							
CRO: Vilinska jama, Podsutulija, Šipanska jama, Šipan	CROBD830	CROPS432-22/CBSS117	BOLD:AEN8669	42.7286	17.8811	ON841941	ON950323
60. *Insulocreagris* sp. 3DH							
CRO: Špilja u Bilopolju 2	CROBD575	CROPS060-21/CBSS24	BOLD:AEI8412	42.9779	17.1416	ON842219	
CRO: Postira, Brač (under stones)	CROBD636	CROPS324-21/CNHM605	BOLD:AEO5791	43.3763	16.638	ON842089	ON950308
61. *Insulocreagris* sp. n. M							
CRO: Močiljska špilja, Osojnik, Dubrovnik	CROBD424	CROPS030-21/CNHM807	BOLD:AEH9824	42.6899	18.071	ON842146	
CRO: Tarina špilja, Šnjati dol, Raba, Opuzen	CROBD837	CROPS153-21/CBSS124	BOLD:AEI4526	42.9814	17.5528	ON841898	
62. *Insulocreagris* sp. n. P1							
CRO: Jama u Potkalaču, Donja Vrućica, Pelješac	CROBD573	CROPS058-21/CBSS22	BOLD:AEI7741	43.0062	17.2109	ON842189	
CRO: Jama u Zajamju, Donja Vrućica, Pelješac	CROBD600	CROPS081-21/CBSS47	BOLD:AEI7741	43.0062	17.2109	ON841827	
CRO: Špilja u Žarmi, Janjina, Pelješac	CROBD612	CROPS091-21/CBSS59	BOLD:AEI7741	42.9354	17.4382	ON842078	

63. *Insulocreagris* sp. n. P2							
CRO: Deveterica, Zagorje, Vukotići, Pelješac	CROBD592	CROPS076-21/CBSS41	BOLD:AEI1168	42.865	17.4847	ON841832	
64. *Insulocreagris* sp. nov. PI							
CRO: Šimunkovića špilja, Orebić, Pelješac	CROBD582	CROPS066-21/CBSS31	BOLD:AEI7742	42.9786	17.1291	ON842224	
CRO: Šimunkovića špilja, Orebić, Pelješac	CROBD584	CROPS068-21/CBSS33	BOLD:AEI7742	42.9786	17.1291	ON841933	
CRO: Šimunkovića špilja, Orebić, Pelješac	CROBD613	CROPS092-21/CBSS60	BOLD:AEI7742	42.9786	17.1291	ON842062	
CRO: Šimunkovića špilja, Orebić, Pelješac	CROBD614	CROPS093-21/CBSS61	BOLD:AEI7742	42.9786	17.1291	ON842258	
CRO: Pišurka, Korčula, Korčula	CROBD820	CROPS431-22/CBSS107	BOLD:AEN8668	42.9594	17.1292	ON842109	ON950321
65. *Insulocreagris* sp. TS							
CRO: Tomasova spilja, Duba pelješka, Pelješac	CROBD273	CROPS239-21/CNHM474	BOLD:AEO4921	43.0142	17.1655	ON841843	
CRO: Tomasova spilja, Duba pelješka, Pelješac	CROBD274	CROPS240-21/CNHM475	BOLD:AEO4921	43.0142	17.1655	ON841931	
CRO: Tomasova spilja, Duba pelješka, Pelješac	CROBD275	CROPS241-21/CNHM476	BOLD:AEO4921	43.0142	17.1655	ON842286	
66. *Neobisium* aff. *dalmatinum* Beier, 1938							
CRO: Spilja Rupine, Točilo od Bute, Duba Pelješka, Pelješac	CROBD591	CROPS075-21/CBSS40	BOLD:AEI4525	43.0052	17.158	ON842225	
CRO: Špilja Lysvulpen, Točilo od Bute, Pelješac	CROBD598	CROPS079-21/CBSS45	BOLD:AEI4525	43.0062	17.1577	ON841806	
CRO: Spilja Rupine, Točilo od Bute, Duba Pelješka, Pelješac	CROBD601	CROPS082-21/CBSS48	BOLD:AEI4525	43.0052	17.158	ON842143	
67. *Neobisium* aff. *elegans* Beier, 1939							
CRO: Romualdova špilja, Limski kanal	CROBD256	CROPS226-21/CNHM457	BOLD:AEO1458	45.1355	13.6548	ON842276	
CRO: Mali Brijun, tvrđava (under stones)	CROBD645	CROPS330-21/CNHM381	BOLD:AEN8734	44.9385	13.7416	ON842066	
68. *Neobisium* aff. *gentile gentile* Beier, 1939							
MN: Vilina pećina, Podi, Crkvice	CROBD1167	CROPS485-22/CBSS540I	BOLD:AEI6261	42.5546	18.6624	ON841919	
CRO: Vučja jama	CROBD1168	CROPS486-22/CBSS291I	BOLD:AEI6261			ON841835	
69. *Neobisium* aff. *heros* Beier, 1938							
MN: Jama kod Blagojevića, Blagojevići, Crkvice	CROBD439	CROPS037-21/CNHM808	BOLD:AEI1653	42.573	18.62	ON841880	ON950298
70. *Neobisium* aff. *oculatum* Ćurčić and Rađa, 2012							
CRO: Ostaševica, Babino polje, Dračevac, Mljet	CROBD1174	CROPS490-22/CBSS444BI	BOLD:AEO6149	42.7489	17.5444	ON842151	
CRO: Ostaševica, Babino polje, Dračevac, Mljet	CROBD1176	CROPS491-22/CBSS435I	BOLD:AEO6149	42.7489	17.5444	ON842160	
CRO: Ostaševica, Babino polje, Dračevac, Mljet	CROBD1177	CROPS492-22/CBSS454I	BOLD:AEO6149	42.7489	17.5444	ON842006	ON950257
71. *Neobisium* aff. *reimoseri* (Beier, 1929)							
CRO: Jamina Vazmineš, Vazmineš, Tramuntana, Cres	CROBD828	CROPS147-21/CBSS115	BOLD:AEI1655	45.1373	14.3422	ON842204	
72. *Neobisium* aff. *stygium* Beier, 1931							
CRO: Natina jama, NP Paklenica	CROBD1138	CROPS462-22/CBSS411I	BOLD:AEO1895	44.3526	15.446	ON842022	
CRO: Gornja Cerovačka špilja, Gračac, Velebit (<2 km from TL)	CROBD346	CROPS278-21/CNHM547	BOLD:AEO1512	44.2734	15.8845	ON842164	
CRO: Jama Vrtlina, Ljubotić, Starigrad Paklenica, Velebit	CROBD620	CROPS318-21/CNHM596	BOLD:AEO1460	44.391	15.3508	ON841886	
CRO: Vodeni tobogan, Zobinovac, Visočica, Velebit	CROBD656	CROPS335-21/CNHM613	BOLD:AEO1460	44.4543	15.3219	ON842058	

73. *Neobisium* aff. *spelaeum* (Schiödte, 1847)								
CRO: Ponor Sušik, Drežnica, Lika	CROBD301	CROPS253-21/CNHM502	BOLD:AEO5736	45.8	15.5	ON842091	
CRO: Ponor Sušik, Drežnica, Lika	CROBD302	CROPS254-21/CNHM503	BOLD:AEO5736	45.8	15.5	ON841985	
CRO: Špilja pod Kovačevcom, Bandino selo, Hrvatski Blagaj	CROBD323	CROPS263-21/CNHM524	BOLD:AEO5739	45.2133	15.5804	ON842158	
CRO: Jopićeva špilja, Brebornica, Krnjak	CROBD342	CROPS275-21/CNHM543	BOLD:AEO5739	45.3	15.591	ON841969	ON950273
CRO: Bandin ponor, Cvijanović brdo, Slunj	CROBD657	CROPS336-21/CNHM614	BOLD:AEO5739	45.1795	15.6239	ON841998	
CRO: Špilja na Savića paljevini, Otočac, Mala Kapela	CROBD819	CROPS142-21/CBSS106	BOLD:AEI3139			ON842266	
CRO: Jama autobus, Mala Kapela, Vrhovine	CROBD831	CROPS149-21/CBSS118	BOLD:AEI3139	44.9307	15.4277	ON842185	
CRO: Jama autobus, Mala Kapela, Vrhovine	CROBD847	CROPS158-21/CBSS134	BOLD:AEI3139	44.9307	15.4277	ON841993	
CRO: Jama rudopoljskog zvončara, Padež, Mala Kapela	CROBD848	CROPS159-21/CBSS135	BOLD:AEI3139	44.9307	15.4278	ON841853	
CRO: Jama rudopoljskog zvončara, Padež, Mala Kapela	CROBD887	CROPS171-21/CBSS174	BOLD:AEI3139	44.9307	15.4278	ON841940	
CRO: Špilja iznad Zečevog ponora, Drežničko polje, Ogulin	CROBD902	CROPS448-22/CBSS181I	BOLD:AEO5736	45.1439	15.1131	ON842257	
74. *Neobisium* aff. *svetovidi* Ćurčić, 1988								
CRO: Ivina jama, Stipančeva Krčevina, Krasno, S. Velebit (TL)	CROBD1172	CROPS489-22/CBSS378I	BOLD:AEI1167	44.8369	15.0137	ON842127	
CRO: Špilja u Škorićima, Škorići, Hrvatsko polje (<2 km from TL)	CROBD331	CROPS269-21/CNHM532	BOLD:AEI3703	44.9027	15.1113	ON842248	
CRO: Karabitkin let, Begovača, Sjeverni Velebit (<2 km from TL)	CROBD334	CROPS271-21/CNHM535	BOLD:AEI8483	44.7428	15.0609	ON841968	ON950272
CRO: Skusača jama, Velika Plana, Srednji Velebit, Gospić (<2 km from TL)	CROBD349	CROPS280-21/CNHM550	BOLD:AEO2880	44.6557	15.1349	ON841900	
CRO: Jama pod Budinom kosicom, NP Sjeverni Velebit (<2 km from TL)	CROBD560	CROPS046-21/CBSS9	BOLD:AEI3705	44.8121	14.9667	ON842166	
CRO: Slovačka jama, Hajdučki kukovi, NP Sjeverni Velebit	CROBD565	CROPS051-21/CBSS14	BOLD:AEI8483	44.749	15.0032	ON842174	
CRO: Jama pod Budinom kosicom, NP Sjeverni Velebit (<2 km from TL)	CROBD571	CROPS057-21/CBSS20	BOLD:AEI3705	44.8121	14.9667	ON842256	
CRO: Ciganuša iznad Tudereva, V. Alan, NP S. Velebit (<2 km from TL)	CROBD574	CROPS059-21/CBSS23	BOLD:AEI3702	44.7111	15	ON841836	
CRO: Lubuška jama, Hajdučki kukovi, S. Velebit (<2 km from TL)	CROBD597	CROPS078-21/CBSS44	BOLD:AEI8483	44.763	15.0283	ON842027	
CRO: Ivina jama, Stipančeva Krčevina, Krasno, S. Velebit (TL)	CROBD603	CROPS083-21/CBSS50	BOLD:AEI1167	44.8369	15.0137	ON841890	ON950304
CRO: Ciganuša iznad Tudereva, V. Alan, NP S. Velebit	CROBD605	CROPS085-21/CBSS52	BOLD:AEI3702	44.7111	15	ON842203	
CRO: Lubuška jama, Hajdučki kukovi, S. Velebit (<2 km from TL)	CROBD610	CROPS089-21/CBSS57	BOLD:AEI8483	44.763	15.0283	ON842020	
CRO: Prva poštena, Jama u Rastovcu, Srednji Velebit (<2 km from TL)	CROBD623	CROPS321-21/CNHM599	BOLD:AEI8483	44.6583	15.1335	ON842082	
CRO: Gavranova jama, Bakovac kosinjski, S. Velebit (<2 km from TL)	CROBD798	CROPS132-21/CBSS85I	BOLD:AEI3703	44.7424	15.0863	ON842163	
75. *Neobisium* aff. *vachoni* Heurtault, 1968								
CRO: Glogova jama	CROBD413	CROPS419-22/CNHM809		42.5732	18.3617	ON842117	ON950292
76. *Neobisium carcinoides* (Hermann, 1804)								
CRO: Špilja Pliskovica, Gologorica, Cerovlje, Istra	CROBD216	CROPS194-21/CNHM417	BOLD:AEO6148	45.2530	14.0347	ON841920	
CRO: Lindarski križ, Istra (soil)	CROBD378	CROPS296-21/CNHM572	BOLD:AEO2613	45.2191	13.96	ON842141	ON950279
77. *Neobisium dalmatinum* Beier, 1938								
CRO: Balićeva špilja, Balići, Dugopolje (TL)	CROBD1170	CROPS487-22/CBSS103I	BOLD:AEI6413	43.5728	16.57	ON841907	
CRO: Balićeva špilja, Balići, Dugopolje (TL)	CROBD1171	CROPS488-22/CBSS534I	BOLD:AEI6413	43.5728	16.57	ON842051	ON950256
CRO: Balićeva špilja, Balići, Dugopolje (TL)	CROBD846	CROPS157-21/CBSS133	BOLD:AEI6413	43.5728	16.57	ON842087	

78. Neobisium dinaricum Hadži, 1933							
CRO: Gusarska špilja, Dubravka, Konavle	CROBD1018	CROPS389-22/CNHM810	BOLD:AEN9054	42.5244	18.4235	ON841838	
CRO: Glogova jama, Sniježnica, Kuna Konavoska, Cavtat	CROBD316	CROPS261-21/CNHM517	BOLD:AEN9054	42.5732	18.3617	ON842112	
CRO: Glogova jama, Sniježnica, Kuna Konavoska, Cavtat	CROBD639	CROPS327-21/CNHM608	BOLD:AEN9054	42.5732	18.3617	ON842254	
MN: Velja peć, Budoš, Nikšić	CROBD816	CROPS139-21/CBSS103	BOLD:AEI7739	42.7317	18.952	ON842131	
MN: Jama kod Blagojevića, Blagojevići, Crkvice (TL)	CROBD825	CROPS144-21/CBSS112	BOLD:AEI4523	42.573	18.62	ON842243	
MN: Jama kod Blagojevića, Blagojevići, Crkvice (TL)	CROBD879	CROPS169-21/CBSS166	BOLD:AEI4527	42.573	18.62	ON842004	
79. Neobisium elegans Beier, 1939							
CRO: Mala pećina, Pećine, Plančica, NP S. Velebit (<2 km from TL)	CROBD576	CROPS061-21/CBSS25	BOLD:AEI1169	44.7755	14.9402	ON842014	ON950303
80. Neobisium erythrodactylum (L. Koch, 1873)							
CRO: Maksimir (soil)	CROBD1135	CROPS459-22/CNHM811	BOLD:AEN8735	45.8285	16.024	ON842209	
CRO: Maksimir (soil)	CROBD1136	CROPS460-22/CNHM812	BOLD:AEN8735	45.8285	16.024	ON842124	
CRO: Maksimir (soil)	CROBD1137	CROPS461-22/CNHM813	BOLD:AEN8735	45.8285	16.024	ON841860	
CRO: Sljeme (soil)	CROBD749	CROPS356-21/CNHM640	BOLD:AEN8735	45.9162	15.9541	ON841823	
81. Neobisium fuscimanum (C.L. Koch, 1843)							
CRO: Jama Staje 9, Male Mune, Ćićarija	CROBD663	CROPS342-21/CNHM620	BOLD:ACR8063	45.4478	14.1448	ON842252	ON950310
82. Neobisium gentile alternum Beier, 1939							
CRO: Špilja Bijelo jezero, Vukasi, Ponikve, Pelješac	CROBD267	CROPS234-21/CNHM468	BOLD:AEO1462	42.8469	17.6367	ON842152	
CRO: Matijaševa peć, Biokovo	CROBD389	CROPS007-21/CNHM814	BOLD:AEI1610	43.3206	17.1223	ON842021	
CRO: Baba špilja, Biokovo	CROBD397	CROPS011-21/CNHM815	BOLD:AEI1659	43.2565	17.1623	ON842095	
CRO: Jujnovića špilja (Špilja Gradina)	CROBD401	CROPS015-21/CNHM816	BOLD:AEI1654	43.2648	17.1993	ON841891	
CRO: Vilina špilja, Gruda, Konavle	CROBD770	CROPS373-21/CNHM660	BOLD:AEO6150	42.5067	18.3913	ON842093	ON950316
CRO: Jujnovića špilja, Jujnovići, Kozice, Biokovo	CROBD779	CROPS427-22/CNHM669	BOLD:AEI1654	43.2648	17.1993	ON841909	
CRO: Drž gaće I, Ančića staje, Baletići, Zavojane	CROBD845	CROPS434-22/CBSS132	BOLD:AEO6150	43.2583	17.283	ON841961	
CRO: Jama malog ulaza, Zamagorje, Zavojane, Vrgorac	CROBD878	CROPS440-22/CBSS165	BOLD:AEO6150	43.2515	17.2709	ON842246	
83. Neobisium gentile flavum Beier, 1939							
MN: Pećina vojvode Dakovića, Grahovo, Dragaljsko polje	CROBD818	CROPS141-21/CBSS105	BOLD:AEI6410	42.6558	18.6758	ON841977	ON950320
CRO: Vilinska jama, Podsutulija, Šipanska jama, Šipan (TL)	CROBD829	CROPS148-21/CBSS116	BOLD:AEI6903	42.7286	17.8811	ON842037	ON950322
CRO: Japaga iznad Kopren dola, Kopren dol, Mlinište	CROBD844	CROPS156-21/CBSS131	BOLD:AEI6964	42.9839	17.6142	ON841830	
BH: Bjelušica, Zavala, Popovo polje	CROBD888	CROPS172-21/CBSS175	BOLD:AEI0670	42.8454	17.9778	ON842002	
CRO: Vilinska jama, Podsutulija, Šipanska jama, Šipan (TL)	CROBD891	CROPS443-22/CBSS178	BOLD:AEI6903	42.7286	17.8811	ON842005	
CRO: Vilinska jama, Podsutulija, Šipanska jama, Šipan (TL)	CROBD893	CROPS444-22/CBSS180	BOLD:AEI6903	42.7286	17.8811	ON841793	
84. Neobisium gentile gentile Beier, 1939							
MN: Vilina pećina, Podi, Crkvice	CROBD827	CROPS146-21/CBSS114	BOLD:AEI6261	42.56	18.629	ON842114	
MN: Pećina u vododerini pokraj Golubinje pećine, Njeguši	CROBD871	CROPS166-21/CBSS158	BOLD:AEI0079	42.4221	18.8001	ON841875	
MN: Vilina pećina, Podi, Crkvice	CROBD872	CROPS437-22/CBSS159	BOLD:AEI6261	42.56	18.629	ON842169	ON950328
MN: Vilina pećina, Podi, Crkvice	CROBD873	CROPS167-21/CBSS160	BOLD:AEI6261	42.56	18.629	ON842231	

85. *Neobisium heros* Beier, 1938							
CRO: Jezero, Snježnica, Konavle	CROBD419	CROPS027-21/CNHM817	BOLD:AEI4524	42.5804	18.3561	ON842182	ON950293
MN: Jama kod Blagojevića, Blagojevići, Crkvice	CROBD880	CROPS441-22/CBSS167	BOLD:AEN9055	42.573	18.62	ON841808	
86. *Neobisium insulare* Beier, 1938							
CRO: Zovnjača, Biloši, Podbablje, Imotski	CROBD203	CROPS185-21/CNHM406	BOLD:AEI6904	43.403	17.1385	ON841841	
CRO: Zovnjača, Biloši, Podbablje, Imotski	CROBD235	CROPS209-21/CNHM436	BOLD:AEI6904	43.403	17.1385	ON841935	
CRO: Zovnjača, Biloši, Podbablje, Imotski	CROBD236	CROPS210-21/CNHM437	BOLD:AEI6904	43.403	17.1385	ON841982	
CRO: Zovnjača, Biloši, Podbablje, Imotski	CROBD257	CROPS227-21/CNHM458	BOLD:AEI6904	43.403	17.1385	ON841960	
CRO: Zovnjača, Biloši, Podbablje, Imotski	CROBD260	CROPS230-21/CNHM461	BOLD:AEI6904	43.403	17.1385	ON841795	
CRO: Zovnjača, Biloši, Podbablje, Imotski	CROBD286	CROPS417-22/CNHM487	BOLD:AEI6904	43.403	17.1385	ON841809	
CRO: Zovnjača, Biloši, Podbablje, Imotski	CROBD303	CROPS255-21/CNHM504	BOLD:AEI6904	43.403	17.1385	ON841839	
CRO: Led 1 jama, Biokovo	CROBD385	CROPS003-21/CNHM818	BOLD:AEI6904	43.344	17.0576	ON842211	ON950281
CRO: Benova (Malena) jama, Biokovo	CROBD408	CROPS020-21/CNHM819	BOLD:AEI6904	43.3025	17.0593	ON842126	ON950288
87. *Neobisium maderi* Beier, 1938							
CRO: Njemica, Bukov vrh, Biokovo	CROBD901	CROPS381-21/CNHM677	BOLD:AEI3142	43.3083	17.0778	ON841818	
CRO: Jama za Supinom, Supin, Podgora, Biokovo	CROBD912	CROPS453-22/CBSS191	BOLD:AEI3142	43.2505	17.1133	ON841804	
CRO: Jama za Supinom, Supin, Podgora, Biokovo	CROBD916	CROPS455-22/CBSS198	BOLD:AEO2612	43.2505	17.1133	ON842011	
CRO: Tučepska vilenjača, Ravna Vlaška, Makarska, Biokovo	CROBD919	CROPS178-21/CBSS198	BOLD:AEI3142	43.2848	17.0837	ON841801	
88. *Neobisium oculatum* Ćurčić and Rađa, 2012							
CRO: Galičnjak, Okuklje, Mljet (<2 km from TL)	CROBD1011	CROPS383-22/CNHM820	BOLD:AEO1463	42.7202	17.6707	ON841814	
CRO: Jama na Strmici, Kozarica, Mljet (<2 km from TL)	CROBD1144	CROPS466-22/CNHM437I	BOLD:AEO2615	42.7671	17.4535	ON842156	
89. *Neobisium peruni* Ćurčić, 1988							
CRO: Lovrićija 2 jama, Biokovo (<2 km from TL)	CROBD388	CROPS006-21/CNHM821	BOLD:AEI1657	43.3369	17.0608	ON842010	ON950283
CRO: Kuna špilja, Biokovo (<2 km from TL)	CROBD405	CROPS019-21/CNHM822	BOLD:AEI1608	43.3224	17.0547	ON841967	ON950286
90. *Neobisium reimoseri* (Beier, 1929)							
CRO: Rabakova špilja, Rabaki, Ročko polje, Istra	CROBD217	CROPS195-21/CNHM418	BOLD:AEI6902	45.3688	14.0768	ON842132	ON950263
CRO: Jama Žbevnica, Slum, Ćićarija	CROBD294	CROPS250-21/CNHM495	BOLD:AEO1459	45.445	13.9896	ON842039	
CRO: Rabakova špilja, Rabaki, Ročko polje, Istra	CROBD658	CROPS337-21/CNHM615	BOLD:AEI6902	45.3688	14.0768	ON842251	
CRO: Rabakova špilja, Rabaki, Ročko polje, Istra	CROBD659	CROPS338-21/CNHM616	BOLD:AEI6902	45.3688	14.0768	ON841854	
CRO: Jama Staje 9, Male Mune, Ćićarija	CROBD664	CROPS343-21/CNHM621	BOLD:AEO6145	45.4478	14.1448	ON842023	
CRO: Jama Staje 9, Male Mune, Ćićarija	CROBD667	CROPS346-21/CNHM624	BOLD:AEO6145	45.4478	14.1448	ON842099	
CRO: Jama nad Zasten, Mune, Ćićarija	CROBD754	CROPS360-21/CNHM645	BOLD:AEN8736	44.3496	15.413	ON842249	
91. *Neobisium* sp. 1DH							
CRO: Golubnjača, Jasenova korita, NP Plitvička jezera	CROBD1163	CROPS481-22/CBSS478I	BOLD:AEO3978	44.7613	15.5906	ON841916	
92. *Neobisium* sp. nov. KP							
MN: Snježnica kod Milića, Kučka korita, Kučke planine	CROBD835	CROPS152-21/CBSS122	BOLD:AEI2043	42.4782	19.5021	ON842207	

93. *Neobisium* sp. nov. M							
CRO: Jama Oaza, Crnopac, Velebit	CROBD212	CROPS191-21/CNHM413	BOLD:AEO5737	44.253	15.850	ON841944	
CRO: Manita peć, Starigrad Paklenica, NP Paklenica	CROBD371	CROPS292-21/CBSS5N	BOLD:AEO4436	44.3158	15.4763	ON841796	
CRO: Manita peć, Starigrad Paklenica, NP Paklenica	CROBD372	CROPS293-21/CBSS6N	BOLD:AEO4436	44.3158	15.4763	ON842077	ON950277
CRO: Jama Vrtlina, Ljubotić, Starigrad Paklenica, Velebit	CROBD621	CROPS319-21/CNHM597	BOLD:AEO1464	44.391	15.3508	ON842269	
CRO: Jama Muda Labudova, Crnopac	CROBD700	CROPS353-21/CNHM632	BOLD:AEO5737	44.2606	15.8503	ON842013	
94. *Neobisium staudacheri* Hadži, 1933							
CRO: Lovrićija 2 jama, Biokovo (<2 km from TL)	CROBD387	CROPS005-21/CNHM823	BOLD:AEI1611	43.3369	17.0608	ON842090	ON950282
CRO: Jama na rasjedu, Biokovo (<2 km from TL)	CROBD407	CROPS418-22/CNHM824	BOLD:AEI1611			ON841822	ON950287
95. *Neobisium stribogi* Ćurčić, 1988							
CRO: Jama Oaza, Crnopac, Velebit (<2 km from TL)	CROBD201	CROPS183-21/CNHM404	BOLD:AEI1612	44.253	15.850	ON841874	
CRO: Jama Oaza, Crnopac, Velebit (<2 km from TL)	CROBD213	CROPS192-21/CNHM414	BOLD:AEI1612	44.253	15.850	ON841863	
CRO: Jama Oaza, Crnopac, Velebit (<2 km from TL)	CROBD215	CROPS193-21/CNHM416	BOLD:AEI1612	44.253	15.850	ON842140	
96. *Neobisium stygium* Beier, 1931							
CRO: Jama pod Guvnom, Jelov Klanac, Rakovica	CROBD1021	CROPS392-22/CBSS36B	BOLD:AEI1658	44.9876	15.5997	ON841816	
CRO: Drobovnik, Žumberak, Kunčani, Radatovići	CROBD1101	CROPS398-22/CBSS3BI	BOLD:AEO2616	45.7251	15.321	ON841881	
CRO: Provala, Donji Oštrc, Bučari, Žumberak	CROBD1107	CROPS404-22/CBSS9BI	BOLD:AEO1896	45.6962	15.4381	ON841817	
CRO: Provala, Donji Oštrc, Bučari, Žumberak	CROBD1108	CROPS405-22/CBSS10BI	BOLD:AEO1896	45.6962	15.4381	ON842162	
CRO: Treća sreća, Južni Velebit, NP Paklenica	CROBD1139	CROPS463-22/CBSS412I	BOLD:AEO2614			ON842059	
CRO: Jama među cestama, NP Plitvička jezera	CROBD1157	CROPS475-22/CBSS472I	BOLD:AEO0581	44.9257	15.5765	ON842214	
CRO: Jama kraj ceste u Plitvičkom klancu, NP Plitvička jezera	CROBD1159	CROPS477-22/CBSS474I	BOLD:AEO0581	44.9131	15.6004	ON841964	
CRO: Fosilka, Japage, S. Velebit (<2 km from TL)	CROBD207	CROPS188-21/CNHM410	BOLD:AEI1656	44.6232	15.1306	ON841885	
CRO: Fosilka, Japage, S. Velebit (<2 km from TL)	CROBD209	CROPS190-21/CNHM412	BOLD:AEI1656	44.623	15.131	ON842085	
CRO: Treća sreća, Južni Velebit, NP Paklenica	CROBD293	CROPS249-21/CNHM494	BOLD:AEN8737			ON842080	
CRO: Donja Baraćeva špilja, Nova Kršlja, Rakovica (<2 km from TL)	CROBD328	CROPS266-21/CNHM529	BOLD:AEI6260	44.984	15.7227	ON842075	ON950270
CRO: Josipova jama u Boštu, Duga Resa	CROBD329	CROPS267-21/CNHM530	BOLD:AEO5279	45.4065	15.4945	ON841896	
CRO: Ralićeva špilja, Lipovac, Rakovica	CROBD330	CROPS268-21/CNHM531	BOLD:AEI1658	45.0282	15.6715	ON841794	ON950271
CRO: Bandiera rossa, Japage, S. Velebit	CROBD332	CROPS270-21/CNHM533	BOLD:AEI1656	44.6437	15.1297	ON842003	
CRO: Kalvarija, Bužim, Gospić (<2 km from TL)	CROBD335	CROPS272-21/CNHM536	BOLD:AEI1656	44.5898	15.224	ON841887	
CRO: Prva poštena, Jama u Rastovcu, Srednji Velebit (<2 km from TL)	CROBD339	CROPS274-21/CNHM540	BOLD:AEI1656	44.6596	15.1383	ON841908	
CRO: Tejina jama, Kestenak, Sića	CROBD343	CROPS276-21/CNHM544	BOLD:AEO5280	45.3321	15.4817	ON841828	
CRO: Tejina jama, Kestenak, Sića	CROBD344	CROPS277-21/CNHM545	BOLD:AEO5280	45.3321	15.4817	ON842136	
CRO: Skusača jama, Velika Plana, Srednji Velebit, Gospić	CROBD347	CROPS279-21/CNHM548	BOLD:AEI1656	44.6557	15.1349	ON841970	
CRO: Gornja Baraćeva špilja, Nova Kršlja, Rakovica (<2 km from TL)	CROBD351	CROPS281-21/CNHM552	BOLD:AEI6260	44.9839	15.7217	ON841948	
CRO: Dumenčića špilja, Keseri, Stara Kršlja, Rakovica	CROBD522	CROPS303-21/CNHM580	BOLD:AEI1658	45.0247	15.6692	ON841821	
CRO: Dumenčića špilja, Keseri, Stara Kršlja, Rakovica	CROBD524	CROPS304-21/CNHM582	BOLD:AEI1658	45.0247	15.6692	ON841876	

CRO: Jama Panj, Jezerce, Plitvička jezera	CROBD528	CROPS308-21/CNHM586	BOLD:AEO0582	44.8657	15.6815	ON841945	
CRO: Jama Panj, Jezerce, Plitvička jezera	CROBD529	CROPS309-21/CNHM587	BOLD:AEO0582	44.8657	15.6815	ON842088	
CRO: Jama Panj, Jezerce, Plitvička jezera	CROBD530	CROPS310-21/CNHM588	BOLD:AEO0582	44.8657	15.6815	ON842115	
CRO: Jama na Vrščiću, Kuselj, Plitvička jezera (<2 km from TL)	CROBD531	CROPS311-21/CNHM589	BOLD:AEN9301	44.9496	15.5444	ON841995	ON950301
CRO: Jama na Vrščiću, Kuselj, Plitvička jezera (<2 km from TL)	CROBD532	CROPS312-21/CNHM590	BOLD:AEN9301	44.9496	15.5444	ON841824	
CRO: Jama na Vrščiću, Kuselj, Plitvička jezera (<2 km from TL)	CROBD533	CROPS313-21/CNHM591	BOLD:AEN9301	44.9496	15.5444	ON842150	
CRO: Jama na Vrščiću, Kuselj, Plitvička jezera (<2 km from TL)	CROBD534	CROPS314-21/CNHM592	BOLD:AEN9301	44.9496	15.5444	ON842272	
CRO: Jama na Vrščiću, Kuselj, Plitvička jezera (<2 km from TL)	CROBD535	CROPS315-21/CNHM593	BOLD:AEN9301	44.9496	15.5444	ON842168	
CRO: UPOV, Golubić, NP Sjeverni Velebit (<2 km from TL)	CROBD562	CROPS048-21/CBSS11	BOLD:AEI1656	44.759	15.0426	ON841950	
CRO: Špilja u Štirovači, NP Sjeverni Velebit (<2 km from TL)	CROBD566	CROPS052-21/CBSS15	BOLD:AEI1656	44.6978	15.055	ON842283	
CRO: Jama na Palježu, NP Sjeverni Velebit (<2 km from TL)	CROBD567	CROPS053-21/CBSS16	BOLD:AEI1656	44.8117	14.9466	ON841797	
CRO: Jama pod Budinom kosicom, NP Sjeverni Velebit	CROBD569	CROPS055-21/CBSS18	BOLD:AEI1656	44.8121	14.9667	ON841812	
CRO: Špilja u Štirovači, NP Sjeverni Velebit (<2 km from TL)	CROBD578	CROPS063-21/CBSS27	BOLD:AEI1656	44.6978	15.055	ON841825	
CRO: Špilja u Štirovači, NP Sjeverni Velebit (<2 km from TL)	CROBD580	CROPS064-21/CBSS29	BOLD:AEI1656	44.6978	15.055	ON841849	
CRO: Špilja u Štirovači, NP Sjeverni Velebit (<2 km from TL)	CROBD581	CROPS065-21/CBSS30	BOLD:AEI1656	44.6978	15.055	ON842191	
CRO: Špilja u Štirovači, NP Sjeverni Velebit (<2 km from TL)	CROBD585	CROPS069-21/CBSS34	BOLD:AEI1656	44.6978	15.055	ON842288	
CRO: Špilja u Štirovači, NP Sjeverni Velebit (<2 km from TL)	CROBD593	CROPS077-21/CBSS42	BOLD:AEI1656	44.6978	15.055	ON842068	
CRO: Jama na Paklinama, S. Velebit (<2 km from TL)	CROBD608	CROPS087-21/CBSS55	BOLD:AEI1656	44.8139	14.944	ON842031	
CRO: Jama na Paklinama, S. Velebit (<2 km from TL)	CROBD609	CROPS088-21/CBSS56	BOLD:AEI1656	44.8139	14.944	ON841895	
CRO: Dumenčića špilja, Keseri, Stara Kršlja, Rakovica	CROBD622	CROPS320-21/CNHM598	BOLD:AEO1465	45.025	15.669	ON842057	
CRO: Jama kod Sekićeve Krčevine, Dučići, Krasno (<2 km from TL)	CROBD624	CROPS420-22/CNHM600	BOLD:AEI1656	44.8188	15.1091	ON842137	
CRO: Jama Staje 9, Male Mune, Ćićarija	CROBD665	CROPS344-21/CNHM622	BOLD:AEO0562	45.4478	14.1448	ON841930	
CRO: Jama Staje 9, Male Mune, Ćićarija	CROBD666	CROPS345-21/CNHM623	BOLD:AEO0562	45.4478	14.1448	ON841913	
CRO: Božićeva špilja, Božić, Kordunski ljeskovac, Rakovica	CROBD675	CROPS097-21/CBSS1B	BOLD:AEI1658	45	15.7765	ON841924	
CRO: Božićeva špilja, Božić, Kordunski ljeskovac, Rakovica	CROBD676	CROPS098-21/CBSS2B	BOLD:AEI1658	45	15.7765	ON842118	
CRO: Dumenčića špilja, Keseri, Stara Kršlja, Rakovica	CROBD677	CROPS099-21/CBSS6B	BOLD:AEI1658	45.0247	15.6692	ON841983	
CRO: Dumenčića špilja, Keseri, Stara Kršlja, Rakovica	CROBD678	CROPS100-21/CBSS7B	BOLD:AEI1658	45.0247	15.6692	ON841921	
CRO: Gornja Baraćeva špilja, Nova Kršlja, Rakovica (<2 km from TL)	CROBD681	CROPS103-21/CBSS10B	BOLD:AEI6260	44.9839	15.7217	ON841962	
CRO: Gornja Baraćeva špilja, Nova Kršlja, Rakovica (<2 km from TL)	CROBD687	CROPS108-21/CBSS16B	BOLD:AEI6260	44.9839	15.7217	ON842268	
CRO: Dumenčića špilja, Keseri, Stara Kršlja, Rakovica	CROBD689	CROPS110-21/CBSS19B	BOLD:AEI1658	45.0247	15.6692	ON841840	
CRO: Donja Baraćeva špilja, Nova Kršlja, Rakovica (<2 km from TL)	CROBD691	CROPS112-21/CBSS21B	BOLD:AEI1658	44.984	15.7227	ON842094	
CRO: Donja Baraćeva špilja, Nova Kršlja, Rakovica (<2 km from TL)	CROBD693	CROPS113-21/CBSS23B	BOLD:AEI1658	44.984	15.7227	ON842180	
CRO: Dumenčića špilja, Keseri, Stara Kršlja, Rakovica	CROBD695	CROPS114-21/CBSS27B	BOLD:AEI1658	45.0247	15.6692	ON841946	
CRO: Mijatova jama, Mateško selo, Generalski Stol	CROBD703	CROPS354-21/CNHM635	BOLD:AEO1038	45.3251	15.4095	ON842245	
CRO: Donja Baraćeva špilja, Nova Kršlja, Rakovica (<2 km from TL)	CROBD744	CROPS125-21/CBSS42B	BOLD:AEI6260	44.984	15.7227	ON842234	

CRO: Božićeva špilja, Božić, Kordunski ljeskovac, Rakovica	CROBD746	CROPS126-21/CBSS46B	BOLD:AEI1658	45	15.7765	ON842213	
CRO: Dumenčića špilja, Keseri, Stara Kršlja, Rakovica	CROBD747	CROPS127-21/CBSS49B	BOLD:AEI1658	45.0247	15.6692	ON841873	
CRO: Dumenčića špilja, Keseri, Stara Kršlja, Rakovica	CROBD748	CROPS355-21/CNHM639	BOLD:AEI1658	45.025	15.669	ON841905	
CRO: Vela špilja u krugu, Studena, Klana	CROBD752	CROPS358-21/CNHM643	BOLD:AEI6411	45.4466	14.4245	ON841829	
CRO: Jama nad Zasten, Mune, Ćićarija	CROBD753	CROPS359-21/CNHM644	BOLD:AEO0562	44.3496	15.413	ON842161	
CRO: Pećina na Čakovcu, Bobići, Mlakva, Lika	CROBD756	CROPS361-21/CNHM646	BOLD:AEO3949	44.6976	15.2804	ON841867	
CRO: Pećina na Čakovcu, Bobići, Mlakva, Lika	CROBD757	CROPS362-21/CNHM647	BOLD:AEO3949	44.6976	15.2804	ON842193	
CRO: Šoića pećina, Podjelar, Gornji Kosinj, Lika	CROBD758	CROPS363-21/CNHM648	BOLD:AEO3948	44.7079	15.2648	ON841996	
CRO: Jama u Bukvama, Vršeljci, Velebit	CROBD783	CROPS128-21/CBSS70I	BOLD:AEI1656	44.6726	15.1153	ON842250	
CRO: Jama ispod heliodroma, Klek, Ogulin, Gorski kotar	CROBD786	CROPS129-21/CBSS73I	BOLD:AEI6407	45.2602	15.1443	ON842177	
CRO: Špilja Atila, Vršeljci, Velebit (<2 km from TL)	CROBD787	CROPS130-21/CBSS74I	BOLD:AEI1656	44.6778	15.1107	ON841992	
CRO: Ponor Štirovača, Velebit	CROBD797	CROPS131-21/CBSS84I	BOLD:AEI1656			ON841872	
CRO: Kozjakuša jama, Vršeljci, Velebit (<2 km from TL)	CROBD799	CROPS133-21/CBSS86I	BOLD:AEI1656	44.6753	15.109	ON842001	
CRO: Brajdićeva jama, Brajdić vrščić, Jelov klanac, Rakovica	CROBD809	CROPS430-22/CBSS96		44.9787	15.5717	ON841925	
CRO: Jama nesretnog migranta, Vršak, NP Plitvička jezera (<2 km from TL)	CROBD810	CROPS136-21/CBSS97	BOLD:AEI6408	44.9231	15.4586	ON841976	
CRO: Sustav Zračak nade 2, Kaverna u tunelu Učka	CROBD817	CROPS140-21/CBSS104	BOLD:AEI7342	45.312	14.2039	ON841877	
CRO: Jama nesretnog migranta, Vršak, NP Plitvička jezera (<2 km from TL)	CROBD823	CROPS143-21/CBSS110	BOLD:AEI6408	44.9231	15.4586	ON842274	
CRO: Kosturnica na Pobojištu, zaleđe izvora Rječine	CROBD826	CROPS145-21/CBSS113	BOLD:AEI6411			ON841974	
CRO: Špilja Lidarka, Kačje, Šverda, Gorski kotar	CROBD832	CROPS150-21/CBSS119	BOLD:AEI6412	45.5522	14.5062	ON841952	ON950324
CRO: Sponzoruša, Učka	CROBD839	CROPS155-21/CBSS126	BOLD:AEI7342			ON842157	
CRO: Kalvarija, Bužim, Gospić (<2 km from TL)	CROBD864	CROPS163-21/CBSS151	BOLD:AEI1656	44.5856	15.2448	ON841954	
CRO: Jama u potrazi I, Praprotna draga, Gorski kotar	CROBD886	CROPS170-21/CBSS173	BOLD:AEI6412	45.5779	14.5082	ON841939	
CRO: Pećinik, Marković selo, Ogulin	CROBD895	CROPS380-21/CNHM671	BOLD:AEN8738	45.2582	15.1984	ON841866	
CRO: Špilja u Štirovači, NP Sjeverni Velebit (<2 km from TL)	CROBD915	CROPS177-21/CBSS194	BOLD:AEI1656	44.6978	15.055	ON841942	
97. *Neobisium sylvaticum* (C.L. Koch, 1835)							
CRO: Near Kraljevec Stream, Medvednica (under stones)	CROBD1134	CROPS458-22/CNHM825	BOLD:AEO6146	45.8836	15.936	ON842265	
CRO: Plaški, Dretulja spring (under stones)	CROBD233	CROPS207-21/CNHM434	BOLD:AEO5740	45.085	15.367	ON842270	ON950265
CRO: Plaški, Dretulja spring (under stones)	CROBD234	CROPS208-21/CNHM435	BOLD:AEO1461	45.085	15.367	ON842205	
98. *Neobisium tantaleum* Beier, 1938							
CRO: Jama malog ulaza, Zamagorje, Zavojane, Vrgorac	CROBD245	CROPS216-21/CNHM446	BOLD:AEI1609	43.2515	17.2709	ON841878	
CRO: Jama malog ulaza, Zamagorje, Zavojane, Vrgorac	CROBD246	CROPS217-21/CNHM447	BOLD:AEI1609	43.2515	17.2709	ON842236	
CRO: Jama malog ulaza, Zamagorje, Zavojane, Vrgorac	CROBD248	CROPS219-21/CNHM449	BOLD:AEI1609	43.2515	17.2709	ON841810	
CRO: Jama malog ulaza, Zamagorje, Zavojane, Vrgorac	CROBD251	CROPS222-21/CNHM452	BOLD:AEI1609	43.2515	17.2709	ON842239	
CRO: Baba špilja, Biokovo	CROBD399	CROPS013-21/CNHM826	BOLD:AEI1609	43.2565	17.1623	ON841981	
CRO: Spila 2, Jujnovići Kozice, Biokovo	CROBD654	CROPS333-21/CNHM611	BOLD:AEI1609	43.2624	17.204	ON841965	

CRO: Jama pod Gažnovcem, Stilija, Vrgorac	CROBD660	CROPS339-21/CNHM617	BOLD:AEI1609	43.2297	17.3142	ON842119	
CRO: Jama malog ulaza, Zamagorje, Zavojane, Vrgorac	CROBD877	CROPS168-21/CBSS164	BOLD:AEI1609	43.2515	17.2709	ON842261	
CRO: Spila 2, Jujnovići, Kozica, Biokovo	CROBD918	CROPS457-22/CBSS197	BOLD:AEI1609	43.2624	17.204	ON841906	
CRO: Tučepska vilenjača, Ravna Vlaška, Makarska, Biokovo	CROBD920	CROPS179-21/CBSS199	BOLD:AEI1609	43.2848	17.0837	ON842188	
CRO: Spila 2, Jujnovići Kozice, Biokovo	CROBD923	CROPS182-21/CBSS202	BOLD:AEI1609	43.2624	17.204	ON841882	
99. *Neobisium vjetrenicae* Hadži, 1932							
MN: Jama J008, Kučka korita, Kučke planine	CROBD889	CROPS173-21/CBSS176	BOLD:AEI7738	42.4747	19.5247	ON841826	
100. *Protoneobisium biocovense* (Müller, 1931)							
CRO: Nova velika, Ćador, Biokovo (<2 km from TL)	CROBD370	CROPS291-21/CBSS4N	BOLD:AEI1660	43.3288	17.0482	ON841980	
CRO: Mokre noge, Biokovo (<2 km from TL)	CROBD390	CROPS008-21/CNHM827	BOLD:AEI1660	43.2967	17.0906	ON842260	ON950283
CRO: Sistem Velika, Velika, Biokovo (<2 km from TL)	CROBD393	CROPS009-21/CNHM828	BOLD:AEI1660	43.3288	17.0482	ON842044	
CRO: Njemica, Bukov vrh, Biokovo (<2 km from TL)	CROBD898	CROPS445-22/CNHM674	BOLD:AEI1660	43.3083	17.0778	ON842128	
CRO: Njemica, Bukov vrh, Biokovo (<2 km from TL)	CROBD899	CROPS446-22/CNHM675	BOLD:AEI1660	43.3083	17.0778	ON842217	
CRO: Stara škola, Sv. Jure, Makarska, Biokovo (TL)	CROBD911	CROPS452-22/CBSS190	BOLD:AEI1660	43.3428	17.0429	ON841842	
CRO: Stara škola, Sv. Jure, Makarska, Biokovo (TL)	CROBD913	CROPS454-22/CBSS192	BOLD:AEI1660	43.3428	17.0429	ON842032	
CRO: Nova velika, Ćador, Biokovo (<2 km from TL)	CROBD917	CROPS456-22/CBSS196	BOLD:AEI1660	43.3288	17.0482	ON842181	
101. *Roncus* aff. *belluatii*							
CRO: Šuma kod okretaljke, Pusto polje, Gračac (soil)	CROBD1147	CROPS468-22/CBSS473I	BOLD:AEI6354			ON842028	ON950245
CRO: Baraćeve špilje, Nova Kršlja, Rakovica (under stones)	CROBD739	CROPS120-21/CBSS35B	BOLD:AEI6354	44.984	15.7227	ON842030	
102. *Roncus* aff. *insularis* Beier, 1938							
CRO: Trasirana jama, Kladnjice, Split	CROBD904	CROPS174-21/CBSS183I	BOLD:AEI3141	43.6913	16.2873	ON841870	ON950331
103. *Roncus* aff. *italicus* (Simon, 1896)							
CRO: Lindarski križ, Istra (soil)	CROBD377	CROPS295-21/CNHM571	BOLD:AEO3442	45.2191	13.96	ON841865	
104. *Roncus* aff. *lubricus* L. Koch, 1873							
CRO: Tihinja špilja, Luka, Ston	CROBD1005	CROPS382-22/CNHM829	BOLD:AEO6241	42.867	17.6695	ON841799	
CRO: Jama Golubinica, Donja Banda, Pelješac	CROBD261	CROPS231-21/CNHM462	BOLD:AEO3441	42.971	17.2872	ON842100	
CRO: Tomasova spilja, Duba pelješka, Pelješac	CROBD277	CROPS243-21/CNHM478	BOLD:AEO6051	43.0142	17.1655	ON842047	
CRO: Vukdelija (under stones)	CROBD750	CROPS357-21/CNHM641	BOLD:AEO6147			ON842025	ON950312
CRO: Vilina špilja, Gruda, Konavle	CROBD771	CROPS374-21/CNHM661	BOLD:AEO3434	42.5067	18.3913	ON842237	ON950317
105. *Roncus* aff. *lubricus* 1 L. Koch, 1873							
CRO: Močiljska špilja, Osojnik, Dubrovnik	CROBD1181	CROPS495-22/CBSS207I	BOLD:AEO6240	42.6899	18.071	ON842069	ON950259
106. *Roncus* aff. *lubricus* 2 L. Koch, 1873							
CRO: Špilja u Radinovcima, Dedići, Rastovac, Biokovo	CROBD910	CROPS451-22/CBSS189	BOLD:AEN8739	43.3873	17.0103	ON841834	
107. *Roncus* aff. *lubricus* 3 L. Koch, 1873							
CRO: Čoćina jama, Nova sela, Metković	CROBD289	CROPS247-21/CNHM490	BOLD:AEI6409	43.1112	17.5668	ON842210	
CRO: Maica Golubinka, Bebići, Nova Sela	CROBD815	CROPS138-21/CBSS102	BOLD:AEI6409	43.1245	17.5458	ON841951	

108. *Roncus* aff. *meledae* Ćurčić and Rađa, 2012							
CRO: Male ponte, Goveđari, Mljet (<2 km from TL)	CROBD1143	CROPS465-22/CBSS431I	BOLD:AEO5738	42.7614	17.4308	ON841861	
CRO: Male ponte, Goveđari, Mljet (<2 km from TL)	CROBD208	CROPS189-21/CNHM411	BOLD:AEO5738	42.7614	17.4308	ON841910	
CRO: Špilja kod Nerezinog dola, Goveđari, Mljet (<2 km from TL)	CROBD305	CROPS256-21/CNHM506	BOLD:AEO5738	42.7584	17.4361	ON842170	ON950268
CRO: Špilja kod Nerezinog dola, Goveđari, Mljet (<2 km from TL)	CROBD307	CROPS257-21/CNHM508	BOLD:AEO5738	42.7584	17.4361	ON842279	
CRO: Male ponte, Goveđari, Mljet (<2 km from TL)	CROBD314	CROPS259-21/CNHM515	BOLD:AEO5738	42.7614	17.4308	ON841879	
109. *Roncus* aff. *ragusae* Ćurčić, 2012							
CRO: Šipun, Cavtat	CROBD1117	CROPS411-22/CBSS41I	BOLD:AEO1897	42.5836	18.218	ON841953	
CRO: Šipun, Cavtat	CROBD1123	CROPS415-22/CBSS132I	BOLD:AEO1897	42.5836	18.218	ON841904	
CRO: Vilina špilja, Gruda, Konavle	CROBD762	CROPS367-21/CNHM652	BOLD:AEO3438	42.5067	18.3913	ON842273	ON950313
110. *Roncus anophthalmus* (Ellingsen, 1910)							
CRO: Kukova peć, Brsečine, Trsteno (close to TL)	CROBD325	CROPS264-21/CNHM526	BOLD:AEO3440	42.7287	17.9649	ON841850	
CRO: Kukova peć, Brsečine, Trsteno (close to TL)	CROBD337	CROPS273-21/CNHM538	BOLD:AEO3440	42.7287	17.9649	ON842065	
CRO: Vranja peć (close to TL)	CROBD443	CROPS039-21/CNHM830	BOLD:AEI9253	42.8681	17.8124	ON842215	ON950299
111. *Roncus* aff. *belluatii* 2							
CRO: Kanjon Korane, NP Plitvička jezera (under stones)	CROBD1160	CROPS478-22/CBSS475I	BOLD:AEI9252	44.908	15.6109	ON842060	ON950252
CRO: Otočac (under stones)	CROBD674	CROPS352-21/CNHM631	BOLD:AEI9252	44.8671	15.2693	ON842007	ON950311
CRO: Baraćeve špilje, Nova Kršlja, Rakovica (under stones)	CROBD738	CROPS119-21/CBSS34B	BOLD:AEI9252	44.984	15.7227	ON841929	
112. *Roncus insularis* Beier, 1938							
CRO: Bazgovača, Podgažul, Brač (<2 km from TL)	CROBD1121	CROPS413-22/CBSS115I	BOLD:AEO6243	43.3065	16.6222	ON842024	
CRO: Bazgovača, Podgažul, Brač (<2 km from TL)	CROBD1122	CROPS414-22/CBSS119I	BOLD:AEO6243	43.3065	16.6222	ON842036	
113. *Roncus italicus* (Simon, 1896)							
CRO: Židovske kuće, Žumberak, Budinjak	CROBD1099	CROPS396-22/CBSS1BI	BOLD:AEO2359	45.7882	15.5007	ON842046	
CRO: Židovske kuće, Cerovica	CROBD1100	CROPS397-22/CBSS2BI	BOLD:AEO3437	45.8095	15.4764	ON842008	
CRO: Dolača, Drašči vrh, Žumberak	CROBD1103	CROPS400-22/CBSS5BI	BOLD:AEO3437	45.7396	15.4828	ON841868	
CRO: Bedara, Tihočaj, Žumberak (under stones)	CROBD1106	CROPS403-22/CBSS8BI	BOLD:AEO6242	45.7519	15.5367	ON842116	
114. *Roncus lubricus* L. Koch, 1873							
CRO: Jama u Bratušu, Biokovo	CROBD382	CROPS001-21/CNHM831	BOLD:AEI9209	43.3284	16.9794	ON841975	ON950280
CRO: Špilja u Bilopolju 2, Orebić, Pelješac	CROBD589	CROPS073-21/CBSS38	BOLD:AEI7740	42.9779	17.1416	ON841973	
CRO: Na-Kovana jama, Nakovane, Orebić	CROBD776	CROPS377-21/CNHM666	BOLD:AEI7740	42.9985	17.0877	ON842155	
CRO: Na-Kovana jama, Nakovane, Orebić	CROBD777	CROPS378-21/CNHM667	BOLD:AEI7740	42.9985	17.0877	ON842130	
CRO: Jama iznad Bobovišta, Bobovište, Bijeli Vir	CROBD851	CROPS160-21/CBSS138	BOLD:AEI9254	43.0022	17.6687	ON841819	
115. *Archaeoroncus dalmatinus* (Hadži, 1933)							
CRO: Biokovo	CROBD202	CROPS184-21/CNHM405	BOLD:AEI8346	43.3216	17.0274	ON842017	
CRO: Biokovo	CROBD232	CROPS206-21/CNHM433	BOLD:AEI8346	43.3216	17.0274	ON842042	
CRO: Jama 4, ŽC GO Lećevica, Kladnjice, Split (<2 km from TL)	CROBD906	CROPS175-21/CBSS185I	BOLD:AEI9251	43.6903	16.2805	ON841889	

116. Roncus narentae Dimitrijević and Rađa, 2009							
CRO: Jama u Kučinama, Vukasi, Ston, Pelješac	CROBD616	CROPS094-21/CBSS63	BOLD:AEI3140	42.8621	17.6274	ON842092	
117. Roncus podaga Ćurčić, 1988							
CRO: Golubinka u gaju, Baletići, Zavojane, Vrgorac	CROBD237	CROPS211-21/CNHM438	BOLD:AEO3435	43.2552	17.2659	ON842018	
CRO: Golubinka u gaju, Baletići, Zavojane, Vrgorac	CROBD242	CROPS213-21/CNHM443	BOLD:AEO3435	43.2552	17.2659	ON841903	
118. Roncus ragusae Ćurčić, 2012							
CRO: Bezdan, Vignje, Čilipi (TL)	CROBD410	CROPS021-21/CNHM832	BOLD:AEI0610	42.5304	18.3163	ON842223	ON950289
119. Roncus sp. 1DH							
CRO: Brseč, Istra (soil)	CROBD376	CROPS294-21/CNHM570	BOLD:AEO3439	45.1783	14.2337	ON842102	ON950278
CRO: Šišan, Istra (soil)	CROBD379	CROPS297-21/CNHM573	BOLD:AEN9204	44.8495	13.9649	ON841915	
CRO: Šišan, Istra (soil)	CROBD380	CROPS298-21/CNHM574	BOLD:AEN9204	44.8495	13.9649	ON841899	
120. Roncus sp. 2DH							
CRO: Špilja pod velikim brijestom, Račja Vas, Ćićarija	CROBD218	CROPS196-21/CNHM419	BOLD:AEO3433			ON842081	
121. Roncus sp. 3DH							
CRO: Špilja Tihinja, vrh Kulina, Mali Ston, Pelješac	CROBD554	CROPS041-21/CBSS3	BOLD:AEI8411	42.867	17.6695	ON842110	
122. Roncus sp. 4DH							
CRO: Movrica, Babino polje, Mljet	CROBD1142	CROPS464-22/CBSS427I	BOLD:AEN9202	42.7525	17.5512	ON842120	
CRO: Velika špilja, Blato, Mljet	CROBD1146	CROPS467-22/CBSS430I	BOLD:AEN9202	42.7647	17.472	ON842165	
CRO: Vilinska jama, Podsutulija, Šipanska jama, Šipan	CROBD1183	CROPS497-22/CBSS516I	BOLD:AEN9202	42.7286	17.8811	ON841971	
123. Roncus sp. n. P							
CRO: Predpeć	CROBD426	CROPS031-21/CNHM833	BOLD:AEI7958	42.7226	18.0458	ON842267	ON950295
CRO: Predpeć	CROBD427	CROPS032-21/CNHM834	BOLD:AEI7958	42.7226	18.0458	ON841857	
124. Roncus sp. n. G							
CRO: Golubinka, Čukovica, Goračić, Vidonje, Metković	CROBD229	CROPS203-21/CNHM430	BOLD:AEN9203	42.9922	17.6452	ON842247	
CRO: Golubinka, Čukovica, Goračić, Vidonje, Metković	CROBD230	CROPS204-21/CNHM431	BOLD:AEN9203	42.9922	17.6452	ON842277	
CRO: Golubinka, Čukovica, Goračić, Vidonje, Metković	CROBD231	CROPS205-21/CNHM432	BOLD:AEN9203	42.9922	17.6452	ON841845	
125. Roncus sp. nov. P							
CRO: Spilja od Lastve	CROBD1164	CROPS482-22/CBSS353I	BOLD:AEI3117	43.0069	17.1625	ON841902	ON950254
CRO: Tomasova spilja, Duba pelješka, Pelješac	CROBD271	CROPS237-21/CNHM472	BOLD:AEI3117	43.0142	17.1655	ON842244	
CRO: Tomasova spilja, Duba pelješka, Pelješac	CROBD561	CROPS047-21/CBSS10	BOLD:AEI3117	43.0142	17.1655	ON841922	
CRO: Tomasova spilja, Duba pelješka, Pelješac	CROBD590	CROPS074-21/CBSS39	BOLD:AEI3117	43.0142	17.1655	ON842176	
126. Roncus sp. nov. S							
CRO: Ponor Sušik, Drežnica	CROBD525	CROPS305-21/CNHM583	BOLD:AEO3436	45.8	15.5	ON841986	
CRO: Ponor Sušik, Drežnica	CROBD526	CROPS306-21/CNHM584	BOLD:AEO3436	45.8	15.5	ON842227	
127. Roncus sp. nov. SO							
CRO: Jama na Benkotovu, Vela Straža, Grohote, Šolta	CROBD1195	CROPS507-22/CBSS47I	BOLD:AEO2358	43.3756	16.272	ON841927	ON950262

Olpiidae Banks, 1895							
128. Olpium pallipes (Lucas, 1849)							
CRO: Lovrečina, Brač (under stones)	CROBD634	CROPS508-22/CNHM603	BOLD:AEO5775	43.37	16.66	ON842076	ON950306

Supplementary Table 2

PCR protocols, thermocycling conditions and substitution models used in MrBayes.

PCR protocols

658-bp-long fragment of the mitochondrial cytochrome *c* oxidase subunit I gene (*COI*, the DNA barcode region, Hebert et al., 2003) was amplified for all individuals, using the primer pairs: LCO1490 and HCO2198 (a) (Folmer et al., 1994) or the cocktail primers C_LepFolF and C_LepFolR (b) (Hernández-Triana et al., 2014). Additional ca. 900-bp-long domain I region of *28S* nuclear ribosomal RNA gene (*28S* rRNA) for 41 taxa within family Chthoniidae and 48 taxa within family Neobisiidae was amplified using the primer set 28SpsF1 (5′-ATTACCCGCCGAATTTAAGC-3′) and 28SpsR1 (5′-TCGGAGGGAACCAGCTAC-3′) (c) (Murienne et al., 2008). For *COI*, PCR amplifications was performed in 20 µl reaction mixtures containing 1 x DreamTaq Green Buffer (Thermo Fisher Scientific Inc., US), 0.2 mM dNTPs, 0.4 µM of each primer, 0.625 U of DreamTaq DNA Polymerase (Thermo Fisher Scientific Inc., US), and 1 µl of eluted DNA. For *28S* amplification, the same sample reaction concentrations were used, but with the relevant primer pair. PCR products were purified by adding Exonuclease I (0.05 U/µl), FastAP Thermosensitive Alkaline Phosphatase (0.025 U/µl) enzymatic system (Thermo Fisher Scientific Inc., US). The reaction was carried using the protocol: 1 h at 37°C followed by 20 min at 80°C.

Thermocycling conditions used for target gene amplifications:

COI	LCO1490/HCO2198	a) initial denaturation at 95°C for 2 min, followed by 35 cycles of denaturation at 95°C for 30 s, annealing at 50°C for 30 s, extension at 72°C for 1 min, followed by a final extension step at 72°C for 10 min
	C_LepFolF/ C_LepFolR	b) initial denaturation step of 95°C for 10 min followed first by 5 cycles of 30 s at 95 °C, 30 s at 45°C and 60 s at 72 °C, then by 35 cycles of 30 s at 95 °C, 30 s at 51 °C and 60 s at 72 °C; ended with the final extension step at 72 °C for 7 min
28S	28SpsF1/28SpsR1	c) initial denaturation at 95°C for 2 min, followed by 35 cycles of denaturation at 95°C for 30 s, annealing at 52°C for 30 s, extension at 72°C for 1 min, followed by a final extension step at 72°C for 10 min

Partitions and Bayesian substitution model parameters applied to the used concatenated dataset

Alignment	Gene	Data Subset	Substitution model (BIC)	Parameter used in MrBayes
S2 (untrimmed)	*COI*	Position 1	HKY+I+G	Nst = 2 rates = invgamma
		Position 2	GTR+I+G	Nst = 6 rates = invgamma
		Position 3	TRN+I+G	Nst = 6 rates = invgamma
	28S		GTR+I+G	Nst = 6 rates = invgamma
S2a (trimmed)	*COI*	Position 1	TRN+G	Nst = 6 rates = gamma
		Position 2	GTR+I+G	Nst = 6 rates = invgamma
		Position 3	TRN+I+G	Nst = 6 rates = invgamma
	28S		GTR+G	Nst = 6 rates = gamma
S3 (untrimmed)	*COI*	Position 1	TIM+I+G	Nst = 6 rates = invgamma
		Position 2	GTR+I+G	Nst = 6 rates = invgamma
		Position 3	TIM+I+G	Nst = 6 rates = invgamma
	28S		TRN+G	Nst = 6 rates = gamma
S3a (trimmed)	*COI*	Position 1	TIM+I+G	Nst = 6 rates = invgamma
		Position 2	GTR+I+G	Nst = 6 rates = invgamma
		Position 3	TIM+I+G	Nst = 6 rates = invgamma
	28S		TRN+G	Nst = 6 rates = gamma

Abbreviations: BIC = Bayesian Information Criterion; HKY = Hasegawa-Kishino-Yano model (Hasegawa, Kishino & Yano 1985); GTR = General Time Reversible model (Tavaré, 1986); TRN = Tamura-Nei model (Tamura & Nei 1993); TIM = Transition model; G = Gamma distributed rate variation; I = proportion of invariable sites.

Supplementary Table 3

Downloaded sequences from online repositories used in phylogenetic analyses.

Dataset	Species	BOLD IDs/ GenBank Accession Nos (*COI*)	GenBank Accession Nos (*28S*)
concatenated Chthoniidae subset	*Euscorpius germanus* (C. L. Koch, 1837)	MK421801	MK421707
	Chernetidae sp.	EU559573	EU559494
	Neobisium polonicum Rafalski, 1936	EU559556	EU559457
	Chthonius ischnocheles (Hermann, 1804)	JN018172	JN018386
	Chthonius dacnodes Navás, 1918	JN018171	JN018385
	Chthonius sp. 1	EU559511	EU559438
concatenated Neobisiidae subset	*Euscorpius germanus* (C. L. Koch, 1837)	MK421801	MK421707
	Chernetidae sp.	EU559573	EU559494
haplotype network	*Neobisium sylvaticum* (C.L. Koch, 1835)	GBPSS002-20	
		GBMYR681-15	
		GBPSS240-20	
		GBPSS158-20	
		GBPSS156-20	
		GBPSS010-20	
		GOMYR210-17	
		GBPSS011-20	
		GOMYR205-17	
		GOMYR222-17	
		GOMYR212-17	
		GBMYR121-14	
		GOMYR196-17	
		GOMYR211-17	
		GOMYR198-17	
		GBMYR123-14	
		GBPSS179-20	
		GBMYR109-14	
		GBMYR1318-16	
		GBMYR1198-16	
		FBPSS066-16	
		GBMYR1317-16	
		GBPSS173-20	
		FBPSS078-16	
		GBPSS018-16	
		FBPSS030-16	
		GBMYR757-15	
		GBPSS019-20	
		BSOIL489-18	

Supplementary Table 4

Results of BOLD's "Barcode Gap Analysis". Abbreviation: NN = Nearest Neighbour.

Species	Mean Intra-Sp	Max Intra-Sp	Nearest species	Distance to NN
Rhacochelifer maculatus	0.15	0.15	*Chernes hahnii*	21.67
Chernes hahnii	1.08	1.53	*Rhacochelifer maculatus*	21.67
Chthonius absoloni	0.51	1.52	*Chthonius* sp. nov. PL	4.56
Chthonius aff. *absoloni*	11.73	19	*Chthonius* sp. nov. P	3.65
Chthonius aff. *alpicola*	3.19	4.26	*Chthonius* sp. 1DH	6.23
Chthonius aff. *exarmatus*	0.15	0.15	*Chthonius trebinjensis*	13.98
Chthonius aff. *heterodactylus*	N/A	0	*Chthonius* aff. *subterraneus*	13.54
Chthonius aff. *ischnocheles*	0	0	*Chthonius* sp. nov. V	10.18
Chthonius aff. *occultus*	5.12	8.05	*Chthonius* sp. nov. P	14.13
Chthonius aff. *radjai*	3.19	3.19	*Chthonius* sp. nov. VL	11.4
Chthonius aff. *subterraneus*	N/A	0	*Chthonius* aff**.** *heterodactylus*	13.54
Chthonius exarmatus	0.78	1.65	*Chthonius trebinjensis*	11.7
Chthonius ischnocheles	6.82	12.16	*Chthonius* aff. *ischnocheles*	11.55
Chthonius magnificus	5.28	7.6	*Chthonius trebinjensis*	10.49
Chthonius occultus	2.86	4.71	*Chthonius* sp. nov. P	10.94
Chthonius raridentatus	1.01	1.52	*Chthonius* sp. nov. K	3.95
Chthonius sp. 1DH	N/A	0	*Chthonius* aff. *alpicola*	6.23
Chthonius sp. 2DH	N/A	0	*Chthonius subterraneus*	14.29
Chthonius sp. LI	N/A	0	*Chthonius* sp. ZB	0.15
Chthonius sp. LK	N/A	0	*Chthonius* sp. nov. M2	12.77
Chthonius sp. ZB	14.44	14.44	*Chthonius* sp. LI	0.15
Chthonius sp. nov. D	N/A	0	*Chthonius* sp. nov. KO	3.5
Chthonius sp. nov. K	0.71	0.91	*Chthonius raridentatus*	3.95
Chthonius sp. nov. KO	N/A	0	*Chthonius* sp. nov. D	3.5
Chthonius sp. nov. L	1.85	1.85	*Chthonius* sp. nov. V	12.77
Chthonius sp. nov. M1	0.33	0.33	*Chthonius* sp. nov. M2	8.01
Chthonius sp. nov. M2	4.41	4.41	*Chthonius* sp. nov. M1	8.01
Chthonius sp. nov. P	N/A	0	*Chthonius* aff. *absoloni*	3.65
Chthonius sp. nov. PL	0.15	0.15	*Chthonius absoloni*	4.56
Chthonius sp. nov. PLJ	1.34	2.58	*Chthonius* aff. *absoloni*	4.86
Chthonius sp. nov. R	N/A	0	*Chthonius* aff. *absoloni*	10.07
Chthonius sp. nov. V	N/A	0	*Chthonius* sp. nov. M1	8.81
Chthonius sp. nov. VL	1.01	1.52	*Chthonius* aff. *radjai*	11.4
Chthonius subterraneus	8.58	17.63	*Chthonius* sp. nov. KO	10.49
Chthonius trebinjensis	2.51	3.5	*Chthonius magnificus*	10.49
Occidenchthonius sp. PEL	N/A	0	*Chthonius* sp. nov. V	15.96
Troglochthonius aff. *mirabilis*	N/A	0	*Troglochthonius mirabilis*	14.59
Troglochthonius mirabilis	N/A	0	*Troglochthonius* aff. *mirabilis*	14.59
Garypus sp. nov. M	N/A	0	*Roncus* sp. nov. S	23.21
Insulocreagris sp. 1DH	0	0	*Insulocreagris* sp. nov. PI	17.1
Insulocreagris sp. 2DH	N/A	0	*Insulocreagris* sp. 1DH	19.24
Insulocreagris sp. 3DH	8.4	8.4	*Insulocreagris* sp. TS	15.11
Insulocreagris sp. TS	0	0	*Insulocreagris* sp. 3DH	15.11
Insulocreagris sp. n. M	9.62	9.62	*Insulocreagris* sp. 1DH	17.25
Insulocreagris sp. nov. P1	0.81	1.22	*Insulocreagris* sp. nov. PI	8.4
Insulocreagris sp. nov. P2	N/A	0	*Insulocreagris* sp. 3DH	16.95
Insulocreagris sp. nov. PI	2.86	6.95	*Insulocreagris* sp. nov. P1	8.4
Neobisium aff. *dalmatinum*	1.42	2.14	*Neobisium dinaricum*	10.53
Neobisium aff. *elegans*	2.29	2.29	*Neobisium elegans*	10.69
Neobisium aff. *heros*	N/A	0	*Neobisium dinaricum*	9.62
Neobisium aff. *oculatum*	1.02	1.37	*Neobisium oculatum*	3.51
Neobisium aff. *reimoseri*	N/A	0	*Neobisium reimoseri*	6.56

Neobisium aff. *spelaeum*	6.07	11.76	*Neobisium stygium*	3.51
Neobisium aff. *stygium*	2.39	3.36	*Neobisium stribogi*	8.7
Neobisium aff. *svetovidi*	7.01	12.98	*Neobisium* sp. 1DH	4.58
Neobisium aff. *vachoni*	N/A	0	*Neobisium dinaricum*	8.54
Neobisium carcinoides	3.05	3.05	*Neobisium* sp. nov. KP	13.59
Neobisium dalmatinum	1.12	1.68	*Neobisium erythrodactylum*	14.96
Neobisium dinaricum	3.14	4.73	*Neobisium vjetrenicae*	6.87
Neobisium elegans	N/A	0	*Neobisium* aff. *elegans*	10.69
Neobisium erythrodactylum	0	0	*Neobisium* aff. *dalmatinum*	13.28
Neobisium fuscimanum	N/A	0	*Neobisium reimoseri*	13.59
Neobisium gentile	10.69	15.11	*Neobisium* sp. nov. M	13.54
Neobisium heros	8.55	8.55	*Neobisium dinaricum*	9.31
Neobisium insulare	0.54	1.53	*Neobisium staudacheri*	8.4
Neobisium maderi	2.46	4.94	*Neobisium tantaleum*	7.96
Neobisium oculatum	3.82	3.82	*Neobisium* aff. *oculatum*	3.51
Neobisium peruni	5.34	5.34	*Protoneobisium biocovense*	8.55
Neobisium reimoseri	5.57	7.79	*Neobisium* aff. *reimoseri*	6.56
Neobisium sp. 1DH	N/A	0	*Neobisium* aff. *svetovidi*	4.58
Neobisium sp. nov. KP	N/A	0	*Neobisium dinaricum*	8.4
Neobisium sp. nov. M	8.98	13.21	*Neobisium* aff. *elegans*	10.99
Neobisium staudacheri	0.64	0.64	*Protoneobisium biocovense*	5.45
Neobisium stribogi	1.83	2.6	*Neobisium* aff. *stygium*	8.7
Neobisium stygium	7.21	12.82	*Neobisium* aff. *spelaeum*	3.51
Neobisium sylvaticum	2.85	3.36	*Roncus* sp. nov. S	13.59
Neobisium tantaleum	0.5	1.37	*Neobisium maderi*	7.96
Neobisium vjetrenicae	N/A	0	*Neobisium dinaricum*	6.87
Protoneobisium biocovense	1.12	1.98	*Neobisium staudacheri*	5.45
Roncus aff. *belluatii*	1.22	1.22	*Roncus aff. belluatii 2*	10.38
Roncus aff. *insularis*	N/A	0	*Roncus insularis*	14.5
Roncus aff. *italicus*	N/A	0	*Roncus* aff. *belluatii*	12.37
Roncus aff. *lubricus*	14.93	20.31	*Roncus lubricus*	9.62
Roncus aff. *lubricus* 1	N/A	0	*Roncus* sp. nov. S	13.44
Roncus aff. *lubricus* 2	N/A	0	*Roncus lubricus*	12.98
Roncus aff. *lubricus* 3	0	0	*Roncus* aff. *lubricus*	12.37
Roncus aff. *meledae*	0.27	0.46	*Roncus* sp. 4DH	8.55
Roncus aff. *ragusae*	6.62	9.92	*Roncus ragusae*	7.18
Roncus anophthalmus	4.68	7.02	*Roncus lubricus*	18.32
Roncus aff. *belluatii 2*	0.2	0.31	*Roncus italicus*	9.62
Roncus insularis	0.31	0.31	*Roncus* aff. *insularis*	14.5
Roncus italicus	8.45	13.59	*Roncus* aff. *belluatii 2*	9.62
Roncus lubricus	12.61	18.63	*Roncus* aff. *lubricus*	9.62
Roncus narentae	N/A	0	*Roncus* sp. 3DH	14.96
Roncus podaga	0.15	0.15	*Roncus* aff. *insularis*	16.34
Roncus ragusae	N/A	0	*Roncus* aff. *ragusae*	7.18
Roncus sp. 1DH	3.05	4.27	*Roncus italicus*	12.67
Roncus sp. 2DH	N/A	0	*Roncus* aff. *belluatii 2*	11.76
Roncus sp. 3DH	N/A	0	*Roncus narentae*	14.96
Roncus sp. 4DH	0.61	0.92	*Roncus* aff. *meledae*	8.55
Roncus sp. n. P	0.15	0.15	*Roncus* aff. *meledae*	17.71
Roncus sp. nov. G	0.1	0.15	*Roncus* aff. *lubricus*	10.99
Roncus sp. nov. P	0.69	1.37	*Roncus* aff. *lubricus*	12.37
Roncus sp. nov. S	0.15	0.15	*Roncus* aff. *belluatii 2*	11.15
Roncus sp. nov. SO	N/A	0	*Roncus lubricus*	13.89
Olpium pallipes	N/A	0	*Roncus* aff. *lubricus* 3	24.93

Supplementary Methods

Procedures of species delineation methods

Four species delineation methods were used to examine concordance between *COI* sequence clustering and morphological species entities for Dinaric dataset (Alignment S1) and Chthoniidae (Alignment S2) and Neobisiidae (Alignment S3) subsets. ABGD was carried out using ABGD server (https://bioinfo.mnhn.fr/abi/public/abgd) applying Kimura two-parameter substitution model and prior for maximum value of intraspecific divergence between 0.001 and 0.1, with 20 recursive steps. Gap width was set on X = 1.0 for Dinaric dataset and on X = 0.7 for Chthoniidae and Neobisiidae subsets. ASAP was run on the web server (https://bioinfo.mnhn.fr/abi/public/asap/asapweb.html) using p-distances with default settings. bPTP was run on the server (https://species.h-its.org/) using the ML implementation from IQ-TREE within the Bayesian and maximum likelihood framework. Bayesian posterior probabilities for putative species were acquired after running 500,000 generations, sampling every 100 generations, while first 20% of the samples were discarded as burn-in. Globally unique identifier (BIN) was assigned from BOLD database using Refined Single Linkage (RESL) algorithm.

Supplementary Table 5

Detailed results of species delineation methods for Dinaric dataset

ABGD

The initial (prior maximal distance *p* = 3.59e-02), and three subsequent recursive partitions (*p* = 3.59e-03, 2.15e-03 and 1.29-03) revealed 155, 159, 170, and 178 groups, respectively.

Initial partition with prior maximal distance *p*=3.59e-02; Barcode gap distance = 0.058

Group 1 n: 23; id: CROPS417-22|Neobisium insulare|CROBD286 CROPS418-22|Neobisium staudacheri|CROBD407 CROPS445-22|Protoneobisium biocovense|CROBD898 CROPS446-22|Protoneobisium biocovense|CROBD899 CROPS452-22|Protoneobisium biocovense|CROBD911 CROPS453-22|Neobisium maderi|CROBD912 CROPS454-22|Protoneobisium biocovense|CROBD913 CROPS455-22|Neobisium maderi|CROBD916 CROPS456-22|Protoneobisium biocovense|CROBD917 CROPS003-21|Neobisium insulare|CROBD385 CROPS005-21|Neobisium staudacheri|CROBD387 CROPS008-21|Protoneobisium biocovense|CROBD390 CROPS009-21|Protoneobisium biocovense|CROBD393 CROPS020-21|Neobisium insulare|CROBD408 CROPS178-21|Neobisium maderi|CROBD919 CROPS185-21|Neobisium insulare|CROBD203 CROPS209-21|Neobisium insulare|CROBD235 CROPS210-21|Neobisium insulare|CROBD236 CROPS227-21|Neobisium insulare|CROBD257 CROPS230-21|Neobisium insulare|CROBD260 CROPS255-21|Neobisium insulare|CROBD303 CROPS291-21|Protoneobisium biocovense|CROBD370 CROPS381-21|Neobisium maderi|CROBD901

Group 2 n: 7; id: CROPS419-22|Neobisium aff. vachoni|CROBD413 CROPS389-22|Neobisium dinaricum|CROBD1018 CROPS139-21|Neobisium dinaricum|CROBD816 CROPS144-21|Neobisium dinaricum|CROBD825 CROPS169-21|Neobisium dinaricum|CROBD879 CROPS261-21|Neobisium dinaricum|CROBD316 CROPS327-21|Neobisium dinaricum|CROBD639

Group 3 n: 48; id: CROPS420-22|Neobisium stygium|CROBD624 CROPS463-22|Neobisium stygium|CROBD1139 CROPS048-21|Neobisium stygium|CROBD562 CROPS052-21|Neobisium stygium|CROBD566 CROPS053-21|Neobisium stygium|CROBD567 CROPS055-21|Neobisium stygium|CROBD569 CROPS063-21|Neobisium stygium|CROBD578 CROPS064-21|Neobisium stygium|CROBD580 CROPS065-21|Neobisium stygium|CROBD581 CROPS069-21|Neobisium stygium|CROBD585 CROPS077-21|Neobisium stygium|CROBD593 CROPS087-21|Neobisium stygium|CROBD608 CROPS088-21|Neobisium stygium|CROBD609 CROPS103-21|Neobisium stygium|CROBD681 CROPS108-21|Neobisium stygium|CROBD687 CROPS125-21|Neobisium stygium|CROBD744 CROPS128-21|Neobisium stygium|CROBD783 CROPS129-21|Neobisium stygium|CROBD786 CROPS130-21|Neobisium stygium|CROBD787 CROPS131-21|Neobisium stygium|CROBD797 CROPS133-21|Neobisium stygium|CROBD799 CROPS140-21|Neobisium stygium|CROBD817 CROPS145-21|Neobisium stygium|CROBD826 CROPS150-21|Neobisium stygium|CROBD832 CROPS155-21|Neobisium stygium|CROBD839 CROPS163-21|Neobisium stygium|CROBD864 CROPS170-21|Neobisium stygium|CROBD886 CROPS177-21|Neobisium stygium|CROBD915 CROPS188-21|Neobisium stygium|CROBD207 CROPS190-21|Neobisium stygium|CROBD209 CROPS249-21|Neobisium stygium|CROBD293 CROPS263-21|Neobisium aff. spelaeum|CROBD323 CROPS266-21|Neobisium stygium|CROBD328 CROPS270-21|Neobisium stygium|CROBD332 CROPS272-21|Neobisium stygium|CROBD335 CROPS274-21|Neobisium stygium|CROBD339 CROPS275-21|Neobisium aff. spelaeum|CROBD342 CROPS279-21|Neobisium stygium|CROBD347 CROPS281-21|Neobisium stygium|CROBD351 CROPS336-21|Neobisium aff. spelaeum|CROBD657 CROPS344-21|Neobisium stygium|CROBD665 CROPS345-21|Neobisium stygium|CROBD666 CROPS358-21|Neobisium stygium|CROBD752 CROPS359-21|Neobisium stygium|CROBD753 CROPS361-21|Neobisium stygium|CROBD756 CROPS362-21|Neobisium stygium|CROBD757 CROPS363-21|Neobisium stygium|CROBD758 CROPS380-21|Neobisium stygium|CROBD895

Group 4 n: 4; id: CROPS421-22|Chernes hahnii|CROBD632 CROPS300-21|Chernes hahnii|CROBD468 CROPS316-21|Chernes hahnii|CROBD594 CROPS317-21|Chernes hahnii|CROBD595

Group 5 n: 17; id: CROPS422-22|Chthonius absoloni|CROBD686 CROPS423-22|Chthonius absoloni|CROBD694 CROPS424-22|Chthonius absoloni|CROBD705 CROPS425-22|Chthonius absoloni|CROBD706 CROPS479-22|Chthonius sp. nov. PL|CROBD1161 CROPS480-22|Chthonius sp. nov. PL|CROBD1162 CROPS393-22|Chthonius absoloni|CROBD1022 CROPS394-22|Chthonius absoloni|CROBD1023 CROPS104-21|Chthonius absoloni|CROBD682 CROPS105-21|Chthonius absoloni|CROBD683 CROPS106-21|Chthonius absoloni|CROBD684 CROPS107-21|Chthonius absoloni|CROBD685 CROPS111-21|Chthonius absoloni|CROBD690 CROPS116-21|Chthonius absoloni|CROBD697 CROPS117-21|Chthonius absoloni|CROBD698 CROPS118-21|Chthonius absoloni|CROBD699 CROPS123-21|Chthonius absoloni|CROBD742

Group 6 n: 4; id: CROPS426-22|Chthonius exarmatus|CROBD765 CROPS044-21|Chthonius exarmatus|CROBD558 CROPS045-21|Chthonius exarmatus|CROBD559 CROPS238-21|Chthonius exarmatus|CROBD272

Group 7 n: 7; id: CROPS427-22|Neobisium gentile alternum|CROBD779 CROPS434-22|Neobisium gentile alternum|CROBD845 CROPS440-22|Neobisium gentile alternum|CROBD878 CROPS007-21|Neobisium gentile alternum|CROBD389 CROPS011-21|Neobisium gentile alternum|CROBD397 CROPS015-21|Neobisium gentile alternum|CROBD401 CROPS373-21|Neobisium gentile alternum|CROBD770
Group 8 n: 1; id: CROPS428-22|Chthonius aff. subterraneus|CROBD800
Group 9 n: 1; id: CROPS429-22|Chthonius sp. nov. R|CROBD806
Group 10 n: 44; id: CROPS430-22|Neobisium stygium|CROBD809 CROPS448-22|Neobisium aff. spelaeum|CROBD902 CROPS475-22|Neobisium stygium|CROBD1157 CROPS477-22|Neobisium stygium|CROBD1159 CROPS392-22|Neobisium stygium|CROBD1021 CROPS398-22|Neobisium stygium|CROBD1101 CROPS404-22|Neobisium stygium|CROBD1107 CROPS405-22|Neobisium stygium|CROBD1108 CROPS097-21|Neobisium stygium|CROBD675 CROPS098-21|Neobisium stygium|CROBD676 CROPS099-21|Neobisium stygium|CROBD677 CROPS100-21|Neobisium stygium|CROBD678 CROPS110-21|Neobisium stygium|CROBD689 CROPS112-21|Neobisium stygium|CROBD691 CROPS113-21|Neobisium stygium|CROBD693 CROPS114-21|Neobisium stygium|CROBD695 CROPS126-21|Neobisium stygium|CROBD746 CROPS127-21|Neobisium stygium|CROBD747 CROPS136-21|Neobisium stygium|CROBD810 CROPS142-21|Neobisium aff. spelaeum|CROBD819 CROPS143-21|Neobisium stygium|CROBD823 CROPS149-21|Neobisium aff. spelaeum|CROBD831 CROPS158-21|Neobisium aff. spelaeum|CROBD847 CROPS159-21|Neobisium aff. spelaeum|CROBD848 CROPS171-21|Neobisium aff. spelaeum|CROBD887 CROPS253-21|Neobisium aff. spelaeum|CROBD301 CROPS254-21|Neobisium aff. spelaeum|CROBD302 CROPS267-21|Neobisium stygium|CROBD329 CROPS268-21|Neobisium stygium|CROBD330 CROPS276-21|Neobisium stygium|CROBD343 CROPS277-21|Neobisium stygium|CROBD344 CROPS303-21|Neobisium stygium|CROBD522 CROPS304-21|Neobisium stygium|CROBD524 CROPS308-21|Neobisium stygium|CROBD528 CROPS309-21|Neobisium stygium|CROBD529 CROPS310-21|Neobisium stygium|CROBD530 CROPS311-21|Neobisium stygium|CROBD531 CROPS312-21|Neobisium stygium|CROBD532 CROPS313-21|Neobisium stygium|CROBD533 CROPS314-21|Neobisium stygium|CROBD534 CROPS315-21|Neobisium stygium|CROBD535 CROPS320-21|Neobisium stygium|CROBD622 CROPS354-21|Neobisium stygium|CROBD703 CROPS355-21|Neobisium stygium|CROBD748
Group 11 n: 5; id: CROPS431-22|Insulocreagris sp. nov. PI|CROBD820 CROPS066-21|Insulocreagris sp. nov. PI|CROBD582 CROPS068-21|Insulocreagris sp. nov. PI|CROBD584 CROPS092-21|Insulocreagris sp. nov. PI|CROBD613 CROPS093-21|Insulocreagris sp. nov. PI|CROBD614
Group 12 n: 1; id: CROPS432-22|Insulocreagris sp. 2DH|CROBD830
Group 13 n: 4; id: CROPS433-22|Chthoniidae|CROBD841 CROPS436-22|Chthoniidae|CROBD866 CROPS135-21|Chthoniidae|CROBD807 CROPS229-21|Chthoniidae|CROBD259
Group 14 n: 2; id: CROPS435-22|Chthonius sp. nov. L|CROBD865 CROPS101-21|Chthonius sp. nov. L|CROBD679
Group 15 n: 6; id: CROPS437-22|Neobisium gentile gentile|CROBD872 CROPS485-22|Neobisium gentile gentile|CROBD1167 CROPS486-22|Neobisium gentile gentile|CROBD1168 CROPS146-21|Neobisium gentile gentile|CROBD827 CROPS166-21|Neobisium gentile gentile|CROBD871 CROPS167-21|Neobisium gentile gentile|CROBD873
Group 16 n: 1; id: CROPS438-22|Chthoniidae|CROBD875
Group 17 n: 6; id: CROPS439-22|Chthonius trebinjensis|CROBD876 CROPS494-22|Chthonius trebinjensis|CROBD1180 CROPS034-21|Chthonius trebinjensis|CROBD435 CROPS040-21|Chthonius trebinjensis|CROBD444 CROPS265-21|Chthonius trebinjensis|CROBD327 CROPS332-21|Chthonius trebinjensis|CROBD653
Group 18 n: 1; id: CROPS441-22|Neobisium heros|CROBD880
Group 19 n: 3; id: CROPS442-22|Chthoniidae|CROBD885 CROPS282-21|Chthoniidae|CROBD353 CROPS283-21|Chthoniidae|CROBD354
Group 20 n: 3; id: CROPS443-22|Neobisium gentile flavum|CROBD891 CROPS444-22|Neobisium gentile flavum|CROBD893 CROPS148-21|Neobisium gentile flavum|CROBD829
Group 21 n: 3; id: CROPS447-22|Chthonius aff. occultus|CROBD900 CROPS449-22|Chthonius aff. occultus|CROBD905 CROPS002-21|Chthonius aff. occultus|CROBD384
Group 22 n: 1; id: CROPS450-22|Chthoniidae|CROBD909
Group 23 n: 1; id: CROPS451-22|Roncus aff. lubricus 2|CROBD910
Group 24 n: 11; id: CROPS457-22|Neobisium tantaleum|CROBD918 CROPS013-21|Neobisium tantaleum|CROBD399 CROPS168-21|Neobisium tantaleum|CROBD877 CROPS179-21|Neobisium tantaleum|CROBD920 CROPS182-21|Neobisium tantaleum|CROBD923 CROPS216-21|Neobisium tantaleum|CROBD245 CROPS217-21|Neobisium tantaleum|CROBD246 CROPS219-21|Neobisium tantaleum|CROBD248 CROPS222-21|Neobisium tantaleum|CROBD251 CROPS333-21|Neobisium tantaleum|CROBD654 CROPS339-21|Neobisium tantaleum|CROBD660
Group 25 n: 1; id: CROPS508-22|Olpium pallipes|CROBD634
Group 26 n: 3; id: CROPS458-22|Neobisium sylvaticum|CROBD1134 CROPS207-21|Neobisium sylvaticum|CROBD233 CROPS208-21|Neobisium sylvaticum|CROBD234
Group 27 n: 4; id: CROPS459-22|Neobisium erythrodactylum|CROBD1135 CROPS460-22|Neobisium erythrodactylum|CROBD1136 CROPS461-22|Neobisium erythrodactylum|CROBD1137 CROPS356-21|Neobisium erythrodactylum|CROBD749
Group 28 n: 4; id: CROPS462-22|Neobisium aff. stygium|CROBD1138 CROPS278-21|Neobisium aff. stygium|CROBD346 CROPS318-21|Neobisium aff. stygium|CROBD620 CROPS335-21|Neobisium aff.

stygium|CROBD656
Group 29 n: 3; id: CROPS464-22|Roncus sp. 4DH|CROBD1142 CROPS467-22|Roncus sp. 4DH|CROBD1146 CROPS497-22|Roncus sp. 4DH|CROBD1183
Group 30 n: 5; id: CROPS465-22|Roncus aff. meledae|CROBD1143 CROPS189-21|Roncus aff. meledae|CROBD208 CROPS256-21|Roncus aff. meledae|CROBD305 CROPS257-21|Roncus aff. meledae|CROBD307 CROPS259-21|Roncus aff. meledae|CROBD314
Group 31 n: 5; id: CROPS466-22|Neobisium oculatum|CROBD1144 CROPS490-22|Neobisium aff. oculatum|CROBD1174 CROPS491-22|Neobisium aff. oculatum|CROBD1176 CROPS492-22|Neobisium aff. oculatum|CROBD1177 CROPS383-22|Neobisium oculatum|CROBD1011
Group 32 n: 2; id: CROPS468-22|Roncus aff. belluatii|CROBD1147 CROPS120-21|Roncus aff. belluatii|CROBD739
Group 33 n: 6; id: CROPS469-22|Chthonius raridentatus|CROBD1149 CROPS474-22|Chthonius sp. nov. K|CROBD1156 CROPS399-22|Chthonius raridentatus|CROBD1102 CROPS401-22|Chthonius raridentatus|CROBD1104 CROPS262-21|Chthonius sp. nov. K|CROBD321 CROPS307-21|Chthonius sp. nov. K|CROBD527
Group 34 n: 2; id: CROPS470-22|Chthonius sp. LI|CROBD1151 CROPS402-22|Chthonius sp. ZB|CROBD1105
Group 35 n: 3; id: CROPS471-22|Chthonius magnificus|CROBD1152 CROPS366-21|Chthonius magnificus|CROBD761 CROPS368-21|Chthonius magnificus|CROBD763
Group 36 n: 2; id: CROPS472-22|Chthonius aff. ischnocheles|CROBD1154 CROPS473-22|Chthonius aff. ischnocheles|CROBD1155
Group 37 n: 1; id: CROPS476-22|Chthonius subterraneus|CROBD1158
Group 38 n: 3; id: CROPS478-22|Roncus aff. belluatii 2|CROBD1160 CROPS119-21|Roncus aff. belluatii 2|CROBD738 CROPS352-21|Roncus aff. belluatii 2|CROBD674
Group 39 n: 11; id: CROPS481-22|Neobisium sp. 1DH|CROBD1163 CROPS489-22|Neobisium aff. svetovidi|CROBD1172 CROPS046-21|Neobisium aff. svetovidi|CROBD560 CROPS057-21|Neobisium aff. svetovidi|CROBD571 CROPS059-21|Neobisium aff. svetovidi|CROBD574 CROPS083-21|Neobisium aff. svetovidi|CROBD603 CROPS085-21|Neobisium aff. svetovidi|CROBD605 CROPS132-21|Neobisium aff. svetovidi|CROBD798 CROPS134-21|Neobisium aff. svetovidi|CROBD801 CROPS269-21|Neobisium aff. svetovidi|CROBD331 CROPS280-21|Neobisium aff. svetovidi|CROBD349
Group 40 n: 4; id: CROPS482-22|Roncus sp. nov. P|CROBD1164 CROPS047-21|Roncus sp. nov. P|CROBD561 CROPS074-21|Roncus sp. nov. P|CROBD590 CROPS237-21|Roncus sp. nov. P|CROBD271
Group 41 n: 5; id: CROPS483-22|Chthonius magnificus|CROBD1165 CROPS410-22|Chthonius magnificus|CROBD1116 CROPS412-22|Chthonius magnificus|CROBD1120 CROPS033-21|Chthonius magnificus|CROBD432 CROPS154-21|Chthonius magnificus|CROBD838
Group 42 n: 4; id: CROPS484-22|Chthoniidae|CROBD1166 CROPS017-21|Chthoniidae|CROBD403 CROPS350-21|Chthoniidae|CROBD672 CROPS351-21|Chthoniidae|CROBD673
Group 43 n: 3; id: CROPS487-22|Neobisium dalmatinum|CROBD1170 CROPS488-22|Neobisium dalmatinum|CROBD1171 CROPS157-21|Neobisium dalmatinum|CROBD846
Group 44 n: 7; id: CROPS493-22|Chthonius occultus|CROBD1178 CROPS502-22|Chthonius occultus|CROBD1189 CROPS024-21|Chthonius occultus|CROBD416 CROPS028-21|Chthonius occultus|CROBD420 CROPS035-21|Chthonius occultus|CROBD436 CROPS165-21|Chthonius occultus|CROBD869 CROPS244-21|Chthonius occultus|CROBD282
Group 45 n: 1; id: CROPS495-22|Roncus aff. lubricus 1|CROBD1181
Group 46 n: 4; id: CROPS496-22|Chthonius magnificus|CROBD1182 CROPS503-22|Chthonius magnificus|CROBD1190 CROPS416-22|Chthonius magnificus|CROBD1124 CROPS023-21|Chthonius magnificus|CROBD412
Group 47 n: 1; id: CROPS499-22|Chthoniidae|CROBD1186
Group 48 n: 12; id: CROPS506-22|Lasiochernes sp. 3DH|CROBD1194 CROPS386-22|Lasiochernes sp. 3DH|CROBD1015 CROPS067-21|Lasiochernes sp. 3DH|CROBD583 CROPS070-21|Lasiochernes sp. 3DH|CROBD586 CROPS072-21|Lasiochernes sp. 3DH|CROBD588 CROPS080-21|Lasiochernes sp. 3DH|CROBD599 CROPS086-21|Lasiochernes sp. 3DH|CROBD607 CROPS095-21|Lasiochernes sp. 3DH|CROBD617 CROPS236-21|Lasiochernes sp. 3DH|CROBD269 CROPS348-21|Lasiochernes sp. 3DH|CROBD670 CROPS349-21|Lasiochernes sp. 3DH|CROBD671 CROPS371-21|Lasiochernes sp. 3DH|CROBD767
Group 49 n: 1; id: CROPS507-22|Roncus sp. nov. SO|CROBD1195
Group 50 n: 1; id: CROPS382-22|Roncus aff. lubricus|CROBD1005
Group 51 n: 2; id: CROPS384-22|Chthonius sp. nov. M1|CROBD1012 CROPS385-22|Chthonius sp. nov. M1|CROBD1013
Group 52 n: 2; id: CROPS387-22|Chthonius aff. exarmatus|CROBD1016 CROPS388-22|Chthonius aff. exarmatus|CROBD1017
Group 53 n: 2; id: CROPS390-22|Lasiochernes sp. 2DH|CROBD1019 CROPS365-21| Lasiochernes sp. 2DH |CROBD760
Group 54 n: 2; id: CROPS391-22|Chthoniidae|CROBD1020 CROPS364-21|Chthoniidae|CROBD759
Group 55 n: 10; id: CROPS395-22|Chthonius aff. occultus|CROBD1081 CROPS197-21|Chthonius aff. occultus|CROBD220 CROPS198-21|Chthonius aff. occultus|CROBD221 CROPS199-21|Chthonius aff. occultus|CROBD222 CROPS200-21|Chthonius aff. occultus|CROBD223 CROPS201-21|Chthonius aff. occultus|CROBD224 CROPS235-21|Chthonius aff. occultus|CROBD268 CROPS246-21|Chthonius aff. occultus|CROBD284 CROPS375-21|Chthonius aff. occultus|CROBD772 CROPS376-21|Chthonius aff.

occultus|CROBD773
Group 56 n: 1; id: CROPS396-22|Roncus italicus|CROBD1099
Group 57 n: 3; id: CROPS397-22|Roncus italicus|CROBD1100 CROPS400-22|Roncus italicus|CROBD1103 CROPS403-22|Roncus italicus|CROBD1106
Group 58 n: 1; id: CROPS406-22|Chthonius ischnocheles reductus|CROBD1109
Group 59 n: 2; id: CROPS407-22|Chthonius ischnocheles ischnocheles|CROBD1110 CROPS409-22|Chthonius ischnocheles ischnocheles|CROBD1114
Group 60 n: 1; id: CROPS408-22|Chthonius sp. ZB|CROBD1113
Group 61 n: 2; id: CROPS411-22|Roncus aff. ragusae|CROBD1117 CROPS415-22|Roncus aff. ragusae|CROBD1123
Group 62 n: 2; id: CROPS413-22|Roncus insularis|CROBD1121 CROPS414-22|Roncus insularis|CROBD1122
Group 63 n: 1; id: CROPS001-21|Roncus lubricus|CROBD382
Group 64 n: 1; id: CROPS004-21|Chthoniidae|CROBD386
Group 65 n: 2; id: CROPS006-21|Neobisium peruni|CROBD388 CROPS019-21|Neobisium peruni|CROBD405
Group 66 n: 1; id: CROPS010-21|Chthoniidae|CROBD396
Group 67 n: 11; id: CROPS012-21|Chthonius aff. occultus|CROBD398 CROPS014-21|Chthonius aff. occultus|CROBD400 CROPS218-21|Chthonius aff. occultus|CROBD247 CROPS220-21|Chthonius aff. occultus|CROBD249 CROPS221-21|Chthonius aff. occultus|CROBD250 CROPS223-21|Chthonius aff. occultus|CROBD252 CROPS224-21|Chthonius aff. occultus|CROBD253 CROPS225-21|Chthonius aff. occultus|CROBD254 CROPS334-21|Chthonius aff. occultus|CROBD655 CROPS340-21|Chthonius aff. occultus|CROBD661 CROPS341-21|Chthonius aff. occultus|CROBD662
Group 68 n: 1; id: CROPS016-21|Troglochthonius aff. mirabilis|CROBD402
Group 69 n: 1; id: CROPS021-21|Roncus ragusae|CROBD410
Group 70 n: 1; id: CROPS022-21|Troglochthonius mirabilis|CROBD411
Group 71 n: 3; id: CROPS025-21|Chthonius subterraneus subterraneus|CROBD417 CROPS026-21|Chthonius subterraneus subterraneus|CROBD418 CROPS161-21|Chthonius subterraneus subterraneus|CROBD862
Group 72 n: 1; id: CROPS027-21|Neobisium heros|CROBD419
Group 73 n: 1; id: CROPS029-21|Chthoniidae|CROBD423
Group 74 n: 1; id: CROPS030-21|Insulocreagris sp. n. M|CROBD424
Group 75 n: 2; id: CROPS031-21|Roncus sp. n. P|CROBD426 CROPS032-21|Roncus sp. n. P|CROBD427
Group 76 n: 1; id: CROPS036-21|Chthoniidae|CROBD437
Group 77 n: 1; id: CROPS037-21|Neobisium aff. heros|CROBD439
Group 78 n: 1; id: CROPS038-21|Occidenchthonius sp. PEL|CROBD442
Group 79 n: 1; id: CROPS039-21|Roncus anophthalmus|CROBD443
Group 80 n: 1; id: CROPS041-21|Roncus sp. 3DH|CROBD554
Group 81 n: 1; id: CROPS042-21|Chthoniidae|CROBD555
Group 82 n: 1; id: CROPS043-21|Chthoniidae|CROBD557
Group 83 n: 1; id: CROPS049-21|Chthonius sp. nov. V|CROBD563
Group 84 n: 2; id: CROPS050-21|Chthonius aff. radjai|CROBD564 CROPS162-21|Chthonius aff. radjai|CROBD863
Group 85 n: 5; id: CROPS051-21|Neobisium aff. svetovidi|CROBD565 CROPS078-21|Neobisium aff. svetovidi|CROBD597 CROPS089-21|Neobisium aff. svetovidi|CROBD610 CROPS271-21|Neobisium aff. svetovidi|CROBD334 CROPS321-21|Neobisium aff. svetovidi|CROBD623
Group 86 n: 4; id: CROPS054-21|Chthonius aff. absoloni|CROBD568 CROPS056-21|Chthonius aff. absoloni|CROBD570 CROPS071-21|Chthonius aff. absoloni|CROBD587 CROPS084-21|Chthonius sp. nov. P|CROBD604
Group 87 n: 3; id: CROPS058-21|Insulocreagris sp. nov. P1|CROBD573 CROPS081-21|Insulocreagris sp. nov. P1|CROBD600 CROPS091-21|Insulocreagris sp. nov. P1|CROBD612
Group 88 n: 1; id: CROPS060-21|Insulocreagris sp. 3DH|CROBD575
Group 89 n: 1; id: CROPS061-21|Neobisium elegans|CROBD576
Group 90 n: 5; id: CROPS062-21|Chthonius sp. nov. PLJ|CROBD577 CROPS090-21|Chthonius sp. nov. PLJ|CROBD611 CROPS228-21|Chthonius aff. absoloni|CROBD258 CROPS369-21|Chthonius sp. nov. PLJ|CROBD764 CROPS379-21|Chthonius sp. nov. PLJ|CROBD778
Group 91 n: 3; id: CROPS073-21|Roncus lubricus|CROBD589 CROPS377-21|Roncus lubricus|CROBD776 CROPS378-21|Roncus lubricus|CROBD777
Group 92 n: 3; id: CROPS075-21|Neobisium aff. dalmatinum|CROBD591 CROPS079-21|Neobisium aff. dalmatinum|CROBD598 CROPS082-21|Neobisium aff. dalmatinum|CROBD601
Group 93 n: 1; id: CROPS076-21|Insulocreagris sp. nov. P2|CROBD592
Group 94 n: 1; id: CROPS094-21|Roncus narentae|CROBD616
Group 95 n: 3; id: CROPS096-21|Chthonius aff. absoloni|CROBD618 CROPS232-21|Chthonius aff. absoloni|CROBD262 CROPS233-21|Chthonius aff. absoloni|CROBD263
Group 96 n: 1; id: CROPS102-21|Chthonius sp. LK|CROBD680
Group 97 n: 5; id: CROPS109-21|Chthonius subterraneus meuseli|CROBD688 CROPS115-21|Chthonius subterraneus meuseli|CROBD696 CROPS121-21|Chthonius subterraneus meuseli|CROBD740 CROPS122-21|Chthonius subterraneus meuseli|CROBD741 CROPS124-21|Chthonius subterraneus meuseli|CROBD743

Group 98 n: 1; id: CROPS137-21|Chthoniidae|CROBD813
Group 99 n: 2; id: CROPS138-21|Roncus aff. lubricus 3|CROBD815 CROPS247-21|Roncus aff. lubricus 3|CROBD289
Group 100 n: 1; id: CROPS141-21|Neobisium gentile flavum|CROBD818
Group 101 n: 1; id: CROPS147-21|Neobisium aff. reimoseri|CROBD828
Group 102 n: 1; id: CROPS151-21|Chthoniidae|CROBD834
Group 103 n: 1; id: CROPS152-21|Neobisium sp. nov. KP|CROBD835
Group 104 n: 1; id: CROPS153-21|Insulocreagris sp. n. M|CROBD837
Group 105 n: 1; id: CROPS156-21|Neobisium gentile flavum|CROBD844
Group 106 n: 1; id: CROPS160-21|Roncus lubricus|CROBD851
Group 107 n: 2; id: CROPS164-21|Chthonius sp. nov. D|CROBD868 CROPS347-21|Chthonius sp. nov. KO|CROBD669
Group 108 n: 1; id: CROPS172-21|Neobisium gentile flavum|CROBD888
Group 109 n: 1; id: CROPS173-21|Neobisium vjetrenicae|CROBD889
Group 110 n: 1; id: CROPS174-21|Roncus aff. insularis|CROBD904
Group 111 n: 1; id: CROPS175-21|Roncus lubricus|CROBD906
Group 112 n: 1; id: CROPS176-21|Chthoniidae|CROBD907
Group 113 n: 3; id: CROPS180-21|Chthonius sp. nov. VL|CROBD921 CROPS181-21|Chthonius sp. nov. VL|CROBD922 CROPS290-21|Chthonius sp. nov. VL|CROBD368
Group 114 n: 3; id: CROPS183-21|Neobisium stribogi|CROBD201 CROPS192-21|Neobisium stribogi|CROBD213 CROPS193-21|Neobisium stribogi|CROBD215
Group 115 n: 2; id: CROPS184-21|Roncus lubricus|CROBD202 CROPS206-21|Roncus lubricus|CROBD232
Group 116 n: 2; id: CROPS186-21|Insulocreagris sp. 1DH|CROBD204 CROPS302-21|Insulocreagris sp. 1DH|CROBD521
Group 117 n: 2; id: CROPS187-21|Lasiochernes sp. 1DH|CROBD205 CROPS248-21|Lasiochernes sp. 1DH|CROBD290
Group 118 n: 2; id: CROPS191-21|Neobisium sp. nov. M|CROBD212 CROPS353-21|Neobisium sp. nov. M|CROBD700
Group 119 n: 2; id: CROPS194-21|Neobisium carcinoides|CROBD216 CROPS296-21|Neobisium carcinoides|CROBD378
Group 120 n: 3; id: CROPS195-21|Neobisium reimoseri|CROBD217 CROPS337-21|Neobisium reimoseri|CROBD658 CROPS338-21|Neobisium reimoseri|CROBD659
Group 121 n: 1; id: CROPS196-21|Roncus sp. 2DH|CROBD218
Group 122 n: 1; id: CROPS202-21|Chthonius sp. 1DH|CROBD225
Group 123 n: 3; id: CROPS203-21|Roncus sp. nov. G|CROBD229 CROPS204-21|Roncus sp. nov. G|CROBD230 CROPS205-21|Roncus sp. nov. G|CROBD231
Group 124 n: 2; id: CROPS211-21|Roncus podaga|CROBD237 CROPS213-21|Roncus podaga|CROBD242
Group 125 n: 3; id: CROPS212-21|Chthonius aff. alpicola|CROBD241 CROPS214-21|Chthonius aff. alpicola|CROBD243 CROPS245-21|Chthonius aff. alpicola|CROBD283
Group 126 n: 1; id: CROPS215-21|Chthonius sp. 2DH|CROBD244
Group 127 n: 2; id: CROPS226-21|Neobisium aff. elegans|CROBD256 CROPS330-21|Neobisium aff. elegans|CROBD645
Group 128 n: 1; id: CROPS231-21|Roncus aff. lubricus|CROBD261
Group 129 n: 1; id: CROPS234-21|Neobisium gentile alternum|CROBD267
Group 130 n: 3; id: CROPS239-21|Insulocreagris sp. TS|CROBD273 CROPS240-21|Insulocreagris sp. TS|CROBD274 CROPS241-21|Insulocreagris sp. TS|CROBD275
Group 131 n: 1; id: CROPS243-21|Roncus aff. lubricus|CROBD277
Group 132 n: 1; id: CROPS250-21|Neobisium reimoseri|CROBD294
Group 133 n: 2; id: CROPS251-21|Chthoniidae|CROBD298 CROPS252-21|Chthoniidae|CROBD300
Group 134 n: 2; id: CROPS258-21|Chthonius sp. nov. M2|CROBD310 CROPS260-21|Chthonius sp. nov. M2|CROBD315
Group 135 n: 2; id: CROPS264-21|Roncus anophthalmus|CROBD325 CROPS273-21|Roncus anophthalmus|CROBD337
Group 136 n: 1; id: CROPS284-21|Chthoniidae|CROBD355
Group 137 n: 4; id: CROPS285-21|Chthonius ischnocheles|CROBD358 CROPS323-21|Chthonius ischnocheles|CROBD635 CROPS325-21|Chthonius ischnocheles|CROBD637 CROPS326-21|Chthonius ischnocheles|CROBD638
Group 138 n: 4; id: CROPS286-21|Chthoniidae|CROBD359 CROPS287-21|Chthoniidae|CROBD362 CROPS288-21|Chthoniidae|CROBD364 CROPS289-21|Chthoniidae|CROBD365
Group 139 n: 2; id: CROPS292-21|Neobisium sp. nov. M|CROBD371 CROPS293-21|Neobisium sp. nov. M|CROBD372
Group 140 n: 3; id: CROPS294-21|Roncus sp. 1DH|CROBD376 CROPS297-21|Roncus sp. 1DH|CROBD379 CROPS298-21|Roncus sp. 1DH|CROBD380
Group 141 n: 1; id: CROPS295-21|Roncus aff. italicus|CROBD377
Group 142 n: 1; id: CROPS301-21|Garypus sp. nov. M|CROBD519
Group 143 n: 2; id: CROPS305-21|Roncus sp. nov. S|CROBD525 CROPS306-21|Roncus sp. nov. S|CROBD526
Group 144 n: 1; id: CROPS319-21|Neobisium sp. nov. M|CROBD621
Group 145 n: 1; id: CROPS324-21|Insulocreagris sp. 3DH|CROBD636
Group 146 n: 2; id: CROPS328-21|Rhacochelifer maculatus|CROBD641 CROPS331-21|Rhacochelifer maculatus|CROBD646
Group 147 n: 1; id: CROPS329-21|Chthonius aff. heterodactylus|CROBD643
Group 148 n: 1; id: CROPS342-21|Neobisium fuscimanum|CROBD663

Group 149 n: 2; id: CROPS343-21|Neobisium reimoseri|CROBD664 CROPS346-21|Neobisium reimoseri|CROBD667
Group 150 n: 1; id: CROPS357-21|Roncus aff. lubricus|CROBD750
Group 151 n: 1; id: CROPS360-21|Neobisium reimoseri|CROBD754
Group 152 n: 1; id: CROPS367-21|Roncus aff. ragusae|CROBD762
Group 153 n: 1; id: CROPS370-21|Chthoniidae|CROBD766
Group 154 n: 1; id: CROPS372-21|Chthoniidae|CROBD768
Group 155 n: 1; id: CROPS374-21|Roncus aff. lubricus|CROBD771

First recursive partition with prior maximal distance *p*=3.59e-02

Group 1 n: 23; id: CROPS417-22|Neobisium insulare|CROBD286|Neobisium insulare|Subgenus: Blothrus CROPS418-22|Neobisium staudacheri|CROBD407|Neobisium staudacheri|Subgenus: Ommatoblothrus CROPS445-22|Protoneobisium biocovense|CROBD898|Protoneobisium biocovense| CROPS446-22|Protoneobisium biocovense|CROBD899|Protoneobisium biocovense| CROPS452-22|Protoneobisium biocovense|CROBD911|Protoneobisium biocovense| CROPS453-22|Neobisium maderi|CROBD912|Neobisium maderi|Subgenus: Blothrus CROPS454-22|Protoneobisium biocovense|CROBD913|Protoneobisium biocovense| CROPS455-22|Neobisium maderi|CROBD916|Neobisium maderi|Subgenus: Blothrus CROPS456-22|Protoneobisium biocovense|CROBD917|Protoneobisium biocovense| CROPS003-21|Neobisium insulare|CROBD385|Neobisium insulare|Subgenus: Blothrus CROPS005-21|Neobisium staudacheri|CROBD387|Neobisium staudacheri|Subgenus: Ommatoblothrus CROPS008-21|Protoneobisium biocovense|CROBD390|Protoneobisium biocovense| CROPS009-21|Protoneobisium biocovense|CROBD393|Protoneobisium biocovense| CROPS020-21|Neobisium insulare|CROBD408|Neobisium insulare|Subgenus: Blothrus CROPS178-21|Neobisium maderi|CROBD919|Neobisium maderi|Subgenus: Blothrus CROPS185-21|Neobisium insulare|CROBD203|Neobisium insulare|Subgenus: Blothrus CROPS209-21|Neobisium insulare|CROBD235|Neobisium insulare|Subgenus: Blothrus CROPS210-21|Neobisium insulare|CROBD236|Neobisium insulare|Subgenus: Blothrus CROPS227-21|Neobisium insulare|CROBD257|Neobisium insulare|Subgenus: Blothrus CROPS230-21|Neobisium insulare|CROBD260|Neobisium insulare|Subgenus: Blothrus CROPS255-21|Neobisium insulare|CROBD303|Neobisium insulare|Subgenus: Blothrus CROPS291-21|Protoneobisium biocovense|CROBD370|Protoneobisium biocovense| CROPS381-21|Neobisium maderi|CROBD901|Neobisium maderi|Subgenus: Blothrus
Group 2 n: 7; id: CROPS419-22|Neobisium aff. vachoni|CROBD413|Neobisium aff. vachoni|Subgenus: Blothrus CROPS389-22|Neobisium dinaricum|CROBD1018|Neobisium dinaricum|Subgenus: Blothrus CROPS139-21|Neobisium dinaricum|CROBD816|Neobisium dinaricum|Subgenus: Blothrus CROPS144-21|Neobisium dinaricum|CROBD825|Neobisium dinaricum|Subgenus: Blothrus CROPS169-21|Neobisium dinaricum|CROBD879|Neobisium dinaricum|Subgenus: Blothrus CROPS261-21|Neobisium dinaricum|CROBD316|Neobisium dinaricum|Subgenus: Blothrus CROPS327-21|Neobisium dinaricum|CROBD639|Neobisium dinaricum|Subgenus: Blothrus
Group 3 n: 48; id: CROPS420-22|Neobisium stygium|CROBD624|Neobisium stygium|Subgenus: Blothrus CROPS463-22|Neobisium stygium|CROBD1139|Neobisium stygium|Subgenus: Blothrus CROPS048-21|Neobisium stygium|CROBD562|Neobisium stygium|Subgenus: Blothrus CROPS052-21|Neobisium stygium|CROBD566|Neobisium stygium|Subgenus: Blothrus CROPS053-21|Neobisium stygium|CROBD567|Neobisium stygium|Subgenus: Blothrus CROPS055-21|Neobisium stygium|CROBD569|Neobisium stygium|Subgenus: Blothrus CROPS063-21|Neobisium stygium|CROBD578|Neobisium stygium|Subgenus: Blothrus CROPS064-21|Neobisium stygium|CROBD580|Neobisium stygium|Subgenus: Blothrus CROPS065-21|Neobisium stygium|CROBD581|Neobisium stygium|Subgenus: Blothrus CROPS069-21|Neobisium stygium|CROBD585|Neobisium stygium|Subgenus: Blothrus CROPS077-21|Neobisium stygium|CROBD593|Neobisium stygium|Subgenus: Blothrus CROPS087-21|Neobisium stygium|CROBD608|Neobisium stygium|Subgenus: Blothrus CROPS088-21|Neobisium stygium|CROBD609|Neobisium stygium|Subgenus: Blothrus CROPS103-21|Neobisium stygium|CROBD681|Neobisium stygium|Subgenus: Blothrus CROPS108-21|Neobisium stygium|CROBD687|Neobisium stygium|Subgenus: Blothrus CROPS125-21|Neobisium stygium|CROBD744|Neobisium stygium|Subgenus: Blothrus CROPS128-21|Neobisium stygium|CROBD783|Neobisium stygium|Subgenus: Blothrus CROPS129-21|Neobisium stygium|CROBD786|Neobisium stygium|Subgenus: Blothrus CROPS130-21|Neobisium stygium|CROBD787|Neobisium stygium|Subgenus: Blothrus CROPS131-21|Neobisium stygium|CROBD797|Neobisium stygium|Subgenus: Blothrus CROPS133-21|Neobisium stygium|CROBD799|Neobisium stygium|Subgenus: Blothrus CROPS140-21|Neobisium stygium|CROBD817|Neobisium stygium|Subgenus: Blothrus CROPS145-21|Neobisium stygium|CROBD826|Neobisium stygium|Subgenus: Blothrus CROPS150-21|Neobisium stygium|CROBD832|Neobisium stygium|Subgenus: Blothrus CROPS155-21|Neobisium stygium|CROBD839|Neobisium stygium|Subgenus: Blothrus CROPS163-21|Neobisium stygium|CROBD864|Neobisium stygium|Subgenus: Blothrus CROPS170-21|Neobisium stygium|CROBD886|Neobisium stygium|Subgenus: Blothrus CROPS177-21|Neobisium stygium|CROBD915|Neobisium stygium|Subgenus: Blothrus CROPS188-21|Neobisium stygium|CROBD207|Neobisium stygium|Subgenus: Blothrus CROPS190-21|Neobisium stygium|CROBD209|Neobisium stygium|Subgenus: Blothrus CROPS249-21|Neobisium stygium|CROBD293|Neobisium stygium|Subgenus: Blothrus CROPS263-21|Neobisium aff.

spelaeum|CROBD323|Neobisium aff. spelaeum|Subgenus: Blothrus CROPS266-21|Neobisium stygium|CROBD328|Neobisium stygium|Subgenus: Blothrus CROPS270-21|Neobisium stygium|CROBD332|Neobisium stygium|Subgenus: Blothrus CROPS272-21|Neobisium stygium|CROBD335|Neobisium stygium|Subgenus: Blothrus CROPS274-21|Neobisium stygium|CROBD339|Neobisium stygium|Subgenus: Blothrus CROPS275-21|Neobisium aff. spelaeum|CROBD342|Neobisium aff. spelaeum|Subgenus: Blothrus CROPS279-21|Neobisium stygium|CROBD347|Neobisium stygium|Subgenus: Blothrus CROPS281-21|Neobisium stygium|CROBD351|Neobisium stygium|Subgenus: Blothrus CROPS336-21|Neobisium aff. spelaeum|CROBD657|Neobisium aff. spelaeum|Subgenus: Blothrus CROPS344-21|Neobisium stygium|CROBD665|Neobisium stygium|Subgenus: Blothrus CROPS345-21|Neobisium stygium|CROBD666|Neobisium stygium|Subgenus: Blothrus CROPS358-21|Neobisium stygium|CROBD752|Neobisium stygium|Subgenus: Blothrus CROPS359-21|Neobisium stygium|CROBD753|Neobisium stygium|Subgenus: Blothrus CROPS361-21|Neobisium stygium|CROBD756|Neobisium stygium|Subgenus: Blothrus CROPS362-21|Neobisium stygium|CROBD757|Neobisium stygium|Subgenus: Blothrus CROPS363-21|Neobisium stygium|CROBD758|Neobisium stygium|Subgenus: Blothrus CROPS380-21|Neobisium stygium|CROBD895|Neobisium stygium|Subgenus: Blothrus

Group 4 n: 4; id: CROPS421-22|Chernes hahnii|CROBD632|Chernes hahnii| CROPS300-21|Chernes hahnii|CROBD468|Chernes hahnii| CROPS316-21|Chernes hahnii|CROBD594|Chernes hahnii| CROPS317-21|Chernes hahnii|CROBD595|Chernes hahnii|

Group 5 n: 15; id: CROPS422-22|Chthonius absoloni|CROBD686|Chthonius absoloni| CROPS423-22|Chthonius absoloni|CROBD694|Chthonius absoloni| CROPS424-22|Chthonius absoloni|CROBD705|Chthonius absoloni| CROPS425-22|Chthonius absoloni|CROBD706|Chthonius absoloni| CROPS393-22|Chthonius absoloni|CROBD1022|Chthonius absoloni| CROPS394-22|Chthonius absoloni|CROBD1023|Chthonius absoloni| CROPS104-21|Chthonius absoloni|CROBD682|Chthonius absoloni| CROPS105-21|Chthonius absoloni|CROBD683|Chthonius absoloni| CROPS106-21|Chthonius absoloni|CROBD684|Chthonius absoloni| CROPS107-21|Chthonius absoloni|CROBD685|Chthonius absoloni| CROPS111-21|Chthonius absoloni|CROBD690|Chthonius absoloni| CROPS116-21|Chthonius absoloni|CROBD697|Chthonius absoloni| CROPS117-21|Chthonius absoloni|CROBD698|Chthonius absoloni| CROPS118-21|Chthonius absoloni|CROBD699|Chthonius absoloni| CROPS123-21|Chthonius absoloni|CROBD742|Chthonius absoloni|

Group 6 n: 4; id: CROPS426-22|Chthonius exarmatus|CROBD765|Chthonius exarmatus| CROPS044-21|Chthonius exarmatus|CROBD558|Chthonius exarmatus| CROPS045-21|Chthonius exarmatus|CROBD559|Chthonius exarmatus| CROPS238-21|Chthonius exarmatus|CROBD272|Chthonius exarmatus|

Group 7 n: 7; id: CROPS427-22|Neobisium gentile alternum|CROBD779|Neobisium gentile|Subgenus: Neobisium CROPS434-22|Neobisium gentile alternum|CROBD845|Neobisium gentile|Subgenus: Neobisium CROPS440-22|Neobisium gentile alternum|CROBD878|Neobisium gentile|Subgenus: Neobisium CROPS007-21|Neobisium gentile alternum|CROBD389|Neobisium gentile|Subgenus: Neobisium CROPS011-21|Neobisium gentile alternum|CROBD397|Neobisium gentile|Subgenus: Neobisium CROPS015-21|Neobisium gentile alternum|CROBD401|Neobisium gentile|Subgenus: Neobisium CROPS373-21|Neobisium gentile alternum|CROBD770|Neobisium gentile|Subgenus: Neobisium

Group 8 n: 1; id: CROPS428-22|Chthonius aff. subterraneus|CROBD800|Chthonius aff. subterraneus|Chthonius aff. subterraneus meuseli

Group 9 n: 1; id: CROPS429-22|Chthonius sp. nov. R|CROBD806|Chthonius sp. nov. R|

Group 10 n: 44; id: CROPS430-22|Neobisium stygium|CROBD809|Neobisium stygium|Subgenus: Blothrus CROPS448-22|Neobisium aff. spelaeum|CROBD902|Neobisium aff. spelaeum|Subgenus: Blothrus CROPS475-22|Neobisium stygium|CROBD1157|Neobisium stygium|Subgenus: Blothrus CROPS477-22|Neobisium stygium|CROBD1159|Neobisium stygium|Subgenus: Blothrus CROPS392-22|Neobisium stygium|CROBD1021|Neobisium stygium|Subgenus: Blothrus CROPS398-22|Neobisium stygium|CROBD1101|Neobisium stygium|Subgenus: Blothrus CROPS404-22|Neobisium stygium|CROBD1107|Neobisium stygium|Subgenus: Blothrus CROPS405-22|Neobisium stygium|CROBD1108|Neobisium stygium|Subgenus: Blothrus CROPS097-21|Neobisium stygium|CROBD675|Neobisium stygium|Subgenus: Blothrus CROPS098-21|Neobisium stygium|CROBD676|Neobisium stygium|Subgenus: Blothrus CROPS099-21|Neobisium stygium|CROBD677|Neobisium stygium|Subgenus: Blothrus CROPS100-21|Neobisium stygium|CROBD678|Neobisium stygium|Subgenus: Blothrus CROPS110-21|Neobisium stygium|CROBD689|Neobisium stygium|Subgenus: Blothrus CROPS112-21|Neobisium stygium|CROBD691|Neobisium stygium|Subgenus: Blothrus CROPS113-21|Neobisium stygium|CROBD693|Neobisium stygium|Subgenus: Blothrus CROPS114-21|Neobisium stygium|CROBD695|Neobisium stygium|Subgenus: Blothrus CROPS126-21|Neobisium stygium|CROBD746|Neobisium stygium|Subgenus: Blothrus CROPS127-21|Neobisium stygium|CROBD747|Neobisium stygium|Subgenus: Blothrus CROPS136-21|Neobisium stygium|CROBD810|Neobisium stygium|Subgenus: Blothrus CROPS142-21|Neobisium aff. spelaeum|CROBD819|Neobisium aff. spelaeum|Subgenus: Blothrus CROPS143-21|Neobisium stygium|CROBD823|Neobisium stygium|Subgenus: Blothrus CROPS149-21|Neobisium aff. spelaeum|CROBD831|Neobisium aff. spelaeum|Subgenus: Blothrus CROPS158-21|Neobisium aff. spelaeum|CROBD847|Neobisium aff. spelaeum|Subgenus: Blothrus CROPS159-21|Neobisium aff. spelaeum|CROBD848|Neobisium aff. spelaeum|Subgenus: Blothrus CROPS171-21|Neobisium aff. spelaeum|CROBD887|Neobisium aff. spelaeum|Subgenus: Blothrus CROPS253-21|Neobisium aff. spelaeum|CROBD301|Neobisium aff. spelaeum|Subgenus: Blothrus CROPS254-21|Neobisium aff. spelaeum|CROBD302|Neobisium aff. spelaeum|Subgenus: Blothrus CROPS267-21|Neobisium stygium|CROBD329|Neobisium stygium|Subgenus: Blothrus CROPS268-21|Neobisium stygium|CROBD330|Neobisium

stygium|Subgenus: Blothrus CROPS276-21|Neobisium stygium|CROBD343|Neobisium stygium|Subgenus: Blothrus CROPS277-21|Neobisium stygium|CROBD344|Neobisium stygium|Subgenus: Blothrus CROPS303-21|Neobisium stygium|CROBD522|Neobisium stygium|Subgenus: Blothrus CROPS304-21|Neobisium stygium|CROBD524|Neobisium stygium|Subgenus: Blothrus CROPS308-21|Neobisium stygium|CROBD528|Neobisium stygium|Subgenus: Blothrus CROPS309-21|Neobisium stygium|CROBD529|Neobisium stygium|Subgenus: Blothrus CROPS310-21|Neobisium stygium|CROBD530|Neobisium stygium|Subgenus: Blothrus CROPS311-21|Neobisium stygium|CROBD531|Neobisium stygium|Subgenus: Blothrus CROPS312-21|Neobisium stygium|CROBD532|Neobisium stygium|Subgenus: Blothrus CROPS313-21|Neobisium stygium|CROBD533|Neobisium stygium|Subgenus: Blothrus CROPS314-21|Neobisium stygium|CROBD534|Neobisium stygium|Subgenus: Blothrus CROPS315-21|Neobisium stygium|CROBD535|Neobisium stygium|Subgenus: Blothrus CROPS320-21|Neobisium stygium|CROBD622|Neobisium stygium|Subgenus: Blothrus CROPS354-21|Neobisium stygium|CROBD703|Neobisium stygium|Subgenus: Blothrus CROPS355-21|Neobisium stygium|CROBD748|Neobisium stygium|Subgenus: Blothrus

Group 11 n: 1; id: CROPS431-22|Insulocreagris sp. nov. PI|CROBD820|Insulocreagris sp. nov. PI|

Group 12 n: 1; id: CROPS432-22|Insulocreagris sp. 2DH|CROBD830|Insulocreagris sp. 2DH|

Group 13 n: 4; id: CROPS433-22|Chthoniidae|CROBD841||Globochthonius | Globochthonius caligatus CROPS436-22|Chthoniidae|CROBD866||Globochthonius | Globochthonius caligatus CROPS135-21|Chthoniidae|CROBD807||Globochthonius | Globochthonius caligatus CROPS229-21|Chthoniidae|CROBD259||Globochthonius | Globochthonius caligatus

Group 14 n: 2; id: CROPS435-22|Chthonius sp. nov. L|CROBD865|Chthonius sp. nov. L| CROPS101-21|Chthonius sp. nov. L|CROBD679|Chthonius sp. nov. L|

Group 15 n: 5; id: CROPS437-22|Neobisium gentile gentile|CROBD872|Neobisium gentile|Subgenus: Neobisium CROPS485-22|Neobisium gentile gentile|CROBD1167|Neobisium gentile|Subgenus: Neobisium | Neobisium aff. gentile gentile CROPS486-22|Neobisium gentile gentile|CROBD1168|Neobisium gentile|Subgenus: Neobisium | Neobisium aff. gentile gentile CROPS146-21|Neobisium gentile gentile|CROBD827|Neobisium gentile|Subgenus: Neobisium | Neobisium aff. gentile gentile CROPS167-21|Neobisium gentile gentile|CROBD873|Neobisium gentile|Subgenus: Neobisium

Group 16 n: 1; id: CROPS438-22|Chthoniidae|CROBD875||Globochthonius | Globochthonius sp. nov. S

Group 17 n: 6; id: CROPS439-22|Chthonius trebinjensis|CROBD876|Chthonius trebinjensis| CROPS494-22|Chthonius trebinjensis|CROBD1180|Chthonius trebinjensis| CROPS034-21|Chthonius trebinjensis|CROBD435|Chthonius trebinjensis| CROPS040-21|Chthonius trebinjensis|CROBD444|Chthonius trebinjensis| CROPS265-21|Chthonius trebinjensis|CROBD327|Chthonius trebinjensis| CROPS332-21|Chthonius trebinjensis|CROBD653|Chthonius trebinjensis|

Group 18 n: 1; id: CROPS441-22|Neobisium heros|CROBD880|Neobisium heros|Subgenus: Blothrus

Group 19 n: 3; id: CROPS442-22|Chthoniidae|CROBD885||Ephippiochthonius | Ephippiochthonius tetrachelatus CROPS282-21|Chthoniidae|CROBD353||Ephippiochthonius | Ephippiochthonius tetrachelatus CROPS283-21|Chthoniidae|CROBD354||Ephippiochthonius | Ephippiochthonius tetrachelatus

Group 20 n: 3; id: CROPS443-22|Neobisium gentile flavum|CROBD891|Neobisium gentile|Subgenus: Neobisium CROPS444-22|Neobisium gentile flavum|CROBD893|Neobisium gentile|Subgenus: Neobisium CROPS148-21|Neobisium gentile flavum|CROBD829|Neobisium gentile|Subgenus: Neobisium

Group 21 n: 3; id: CROPS447-22|Chthonius aff. occultus|CROBD900|Chthonius aff. occultus| CROPS449-22|Chthonius aff. occultus|CROBD905|Chthonius aff. occultus| CROPS002-21|Chthonius aff. occultus|CROBD384|Chthonius aff. occultus|

Group 22 n: 1; id: CROPS450-22|Chthoniidae|CROBD909||Globochthonius | Globochthonius sp. nov. B

Group 23 n: 1; id: CROPS451-22|Roncus aff. lubricus 2|CROBD910|Roncus aff. lubricus 2|

Group 24 n: 11; id: CROPS457-22|Neobisium tantaleum|CROBD918|Neobisium tantaleum|Subgenus: Blothrus CROPS013-21|Neobisium tantaleum|CROBD399|Neobisium tantaleum|Subgenus: Blothrus CROPS168-21|Neobisium tantaleum|CROBD877|Neobisium tantaleum|Subgenus: Blothrus CROPS179-21|Neobisium tantaleum|CROBD920|Neobisium tantaleum|Subgenus: Blothrus CROPS182-21|Neobisium tantaleum|CROBD923|Neobisium tantaleum|Subgenus: Blothrus CROPS216-21|Neobisium tantaleum|CROBD245|Neobisium tantaleum|Subgenus: Blothrus CROPS217-21|Neobisium tantaleum|CROBD246|Neobisium tantaleum|Subgenus: Blothrus CROPS219-21|Neobisium tantaleum|CROBD248|Neobisium tantaleum|Subgenus: Blothrus CROPS222-21|Neobisium tantaleum|CROBD251|Neobisium tantaleum|Subgenus: Blothrus CROPS333-21|Neobisium tantaleum|CROBD654|Neobisium tantaleum|Subgenus: Blothrus CROPS339-21|Neobisium tantaleum|CROBD660|Neobisium tantaleum|Subgenus: Blothrus

Group 25 n: 1; id: CROPS508-22|Olpium pallipes|CROBD634|Olpium pallipes|

Group 26 n: 3; id: CROPS458-22|Neobisium sylvaticum|CROBD1134|Neobisium sylvaticum|Subgenus: Neobisium CROPS207-21|Neobisium sylvaticum|CROBD233|Neobisium sylvaticum|Subgenus: Neobisium CROPS208-21|Neobisium sylvaticum|CROBD234|Neobisium sylvaticum|Subgenus: Neobisium

Group 27 n: 4; id: CROPS459-22|Neobisium erythrodactylum|CROBD1135|Neobisium erythrodactylum|Subgenus: Neobisium CROPS460-22|Neobisium erythrodactylum|CROBD1136|Neobisium erythrodactylum|Subgenus: Neobisium CROPS461-22|Neobisium erythrodactylum|CROBD1137|Neobisium erythrodactylum|Subgenus: Neobisium CROPS356-21|Neobisium erythrodactylum|CROBD749|Neobisium erythrodactylum|Subgenus: Neobisium

Group 28 n: 4; id: CROPS462-22|Neobisium aff. stygium|CROBD1138|Neobisium aff. stygium|Subgenus: Blothrus

CROPS278-21|Neobisium aff. stygium|CROBD346|Neobisium aff. stygium|Subgenus: Blothrus CROPS318-21|Neobisium aff. stygium|CROBD620|Neobisium aff. stygium|Subgenus: Blothrus CROPS335-21|Neobisium aff. stygium|CROBD656|Neobisium aff. stygium|Subgenus: Blothrus

Group 29 n: 3; id: CROPS464-22|Roncus sp. 4DH|CROBD1142|Roncus sp. 4DH| CROPS467-22|Roncus sp. 4DH|CROBD1146|Roncus sp. 4DH| CROPS497-22|Roncus sp. 4DH|CROBD1183|Roncus sp. 4DH|

Group 30 n: 5; id: CROPS465-22|Roncus aff. meledae|CROBD1143|Roncus aff. meledae| CROPS189-21|Roncus aff. meledae|CROBD208|Roncus aff. meledae| CROPS256-21|Roncus aff. meledae|CROBD305|Roncus aff. meledae| CROPS257-21|Roncus aff. meledae|CROBD307|Roncus aff. meledae| CROPS259-21|Roncus aff. meledae|CROBD314|Roncus aff. meledae|

Group 31 n: 5; id: CROPS466-22|Neobisium oculatum|CROBD1144|Neobisium oculatum|Subgenus: Neobisium CROPS490-22|Neobisium aff. oculatum|CROBD1174|Neobisium aff. oculatum|Subgenus: Neobisium CROPS491-22|Neobisium aff. oculatum|CROBD1176|Neobisium aff. oculatum|Subgenus: Neobisium CROPS492-22|Neobisium aff. oculatum|CROBD1177|Neobisium aff. oculatum|Subgenus: Neobisium CROPS383-22|Neobisium oculatum|CROBD1011|Neobisium oculatum|Subgenus: Neobisium

Group 32 n: 2; id: CROPS468-22|Roncus aff. belluatii|CROBD1147|Roncus aff. belluatii| CROPS120-21|Roncus aff. belluatii|CROBD739|Roncus aff. belluatii|

Group 33 n: 6; id: CROPS469-22|Chthonius raridentatus|CROBD1149|Chthonius raridentatus| CROPS474-22|Chthonius sp. nov. K|CROBD1156|Chthonius sp. nov. K| CROPS399-22|Chthonius raridentatus|CROBD1102|Chthonius raridentatus| CROPS401-22|Chthonius raridentatus|CROBD1104|Chthonius raridentatus| CROPS262-21|Chthonius sp. nov. K|CROBD321|Chthonius sp. nov. K| CROPS307-21|Chthonius sp. nov. K|CROBD527|Chthonius sp. nov. K|

Group 34 n: 2; id: CROPS470-22|Chthonius sp. LI|CROBD1151|Chthonius sp. LI| CROPS402-22|Chthonius sp. ZB|CROBD1105|Chthonius sp. ZB|

Group 35 n: 3; id: CROPS471-22|Chthonius magnificus|CROBD1152|Chthonius magnificus| CROPS366-21|Chthonius magnificus|CROBD761|Chthonius magnificus| CROPS368-21|Chthonius magnificus|CROBD763|Chthonius magnificus|

Group 36 n: 2; id: CROPS472-22|Chthonius aff. ischnocheles|CROBD1154|Chthonius aff. ischnocheles| CROPS473-22|Chthonius aff. ischnocheles|CROBD1155|Chthonius aff. ischnocheles|

Group 37 n: 1; id: CROPS476-22|Chthonius subterraneus|CROBD1158|Chthonius subterraneus|

Group 38 n: 3; id: CROPS478-22|Roncus aff. belluatii 2|CROBD1160|Roncus aff. belluatii 2| CROPS119-21|Roncus aff. belluatii 2|CROBD738|Roncus aff. belluatii 2| CROPS352-21|Roncus aff. belluatii 2|CROBD674|Roncus aff. belluatii 2|

Group 39 n: 11; id: CROPS481-22|Neobisium sp. 1DH|CROBD1163|Neobisium sp. 1DH| CROPS489-22|Neobisium aff. svetovidi|CROBD1172|Neobisium aff. svetovidi|Subgenus: Blothrus CROPS046-21|Neobisium aff. svetovidi|CROBD560|Neobisium aff. svetovidi|Subgenus: Blothrus CROPS057-21|Neobisium aff. svetovidi|CROBD571|Neobisium aff. svetovidi|Subgenus: Blothrus CROPS059-21|Neobisium aff. svetovidi|CROBD574|Neobisium aff. svetovidi|Subgenus: Blothrus CROPS083-21|Neobisium aff. svetovidi|CROBD603|Neobisium aff. svetovidi|Subgenus: Blothrus CROPS085-21|Neobisium aff. svetovidi|CROBD605|Neobisium aff. svetovidi|Subgenus: Blothrus CROPS132-21|Neobisium aff. svetovidi|CROBD798|Neobisium aff. svetovidi|Subgenus: Blothrus CROPS134-21|Neobisium aff. svetovidi|CROBD801|Neobisium aff. svetovidi|Subgenus: Blothrus CROPS269-21|Neobisium aff. svetovidi|CROBD331|Neobisium aff. svetovidi|Subgenus: Blothrus CROPS280-21|Neobisium aff. svetovidi|CROBD349|Neobisium aff. svetovidi|Subgenus: Blothrus

Group 40 n: 4; id: CROPS482-22|Roncus sp. nov. P|CROBD1164|Roncus sp. nov. P| CROPS047-21|Roncus sp. nov. P|CROBD561|Roncus sp. nov. P| CROPS074-21|Roncus sp. nov. P|CROBD590|Roncus sp. nov. P| CROPS237-21|Roncus sp. nov. P|CROBD271|Roncus sp. nov. P|

Group 41 n: 5; id: CROPS483-22|Chthonius magnificus|CROBD1165|Chthonius magnificus| CROPS410-22|Chthonius magnificus|CROBD1116|Chthonius magnificus| CROPS412-22|Chthonius magnificus|CROBD1120|Chthonius magnificus| CROPS033-21|Chthonius magnificus|CROBD432|Chthonius magnificus| CROPS154-21|Chthonius magnificus|CROBD838|Chthonius magnificus|

Group 42 n: 4; id: CROPS484-22|Chthoniidae|CROBD1166||Globochthonius | Globochthonius sp. nov. D CROPS017-21|Chthoniidae|CROBD403||Globochthonius | Globochthonius sp. n. D CROPS350-21|Chthoniidae|CROBD672||Globochthonius | Globochthonius sp. n. D CROPS351-21|Chthoniidae|CROBD673||Globochthonius | Globochthonius sp. n. D

Group 43 n: 3; id: CROPS487-22|Neobisium dalmatinum|CROBD1170|Neobisium dalmatinum|Subgenus: Blothrus CROPS488-22|Neobisium dalmatinum|CROBD1171|Neobisium dalmatinum|Subgenus: Blothrus CROPS157-21|Neobisium dalmatinum|CROBD846|Neobisium dalmatinum|Subgenus: Blothrus

Group 44 n: 7; id: CROPS493-22|Chthonius occultus|CROBD1178|Chthonius occultus| CROPS502-22|Chthonius occultus|CROBD1189|Chthonius occultus| CROPS024-21|Chthonius occultus|CROBD416|Chthonius occultus| CROPS028-21|Chthonius occultus|CROBD420|Chthonius occultus| CROPS035-21|Chthonius occultus|CROBD436|Chthonius occultus| CROPS165-21|Chthonius occultus|CROBD869|Chthonius occultus| CROPS244-21|Chthonius occultus|CROBD282|Chthonius occultus|

Group 45 n: 1; id: CROPS495-22|Roncus aff. lubricus 1|CROBD1181|Roncus aff. lubricus 1|

Group 46 n: 4; id: CROPS496-22|Chthonius magnificus|CROBD1182|Chthonius magnificus| CROPS503-22|Chthonius magnificus|CROBD1190|Chthonius magnificus| CROPS416-22|Chthonius magnificus|CROBD1124|Chthonius magnificus|

CROPS023-21|Chthonius magnificus|CROBD412|Chthonius magnificus|
Group 47 n: 1; id: CROPS499-22|Chthoniidae|CROBD1186||Ephippiochthonius | Ephippiochthonius insularis
Group 48 n: 12; id: CROPS506-22|Lasiochernes|CROBD1194||sp. 3DH CROPS386-22|Lasiochernes|CROBD1015||sp. 3DH CROPS067-21|Lasiochernes|CROBD583||sp. 3DH CROPS070-21|Lasiochernes|CROBD586||sp. 3DH CROPS072-21|Lasiochernes|CROBD588||sp. 3DH CROPS080-21|Lasiochernes|CROBD599||sp. 3DH CROPS086-21|Lasiochernes|CROBD607||sp. 3DH CROPS095-21|Lasiochernes|CROBD617||sp. 3DH CROPS236-21|Lasiochernes|CROBD269||sp. 3DH CROPS348-21|Lasiochernes|CROBD670||sp. 3DH CROPS349-21|Lasiochernes|CROBD671||sp. 3DH CROPS371-21|Lasiochernes|CROBD767||sp. 3DH
Group 49 n: 1; id: CROPS507-22|Roncus sp. nov. SO|CROBD1195|Roncus sp. nov. SO|
Group 50 n: 1; id: CROPS382-22|Roncus aff. lubricus|CROBD1005|Roncus aff. lubricus|
Group 51 n: 2; id: CROPS384-22|Chthonius sp. nov. M1|CROBD1012|Chthonius sp. nov. M1| CROPS385-22|Chthonius sp. nov. M1|CROBD1013|Chthonius sp. nov. M1|
Group 52 n: 2; id: CROPS387-22|Chthonius aff. exarmatus|CROBD1016|Chthonius aff. exarmatus| CROPS388-22|Chthonius aff. exarmatus|CROBD1017|Chthonius aff. exarmatus|
Group 53 n: 2; id: CROPS390-22|Lasiochernes|CROBD1019||sp. 2DH CROPS365-21|Lasiochernes|CROBD760||sp. 2DH
Group 54 n: 2; id: CROPS391-22|Chthoniidae|CROBD1020||Globochthonius | Globochthonius caligatus CROPS364-21|Chthoniidae|CROBD759||Globochthonius | Globochthonius caligatus
Group 55 n: 4; id: CROPS395-22|Chthonius aff. occultus|CROBD1081|Chthonius aff. occultus| CROPS246-21|Chthonius aff. occultus|CROBD284|Chthonius aff. occultus| CROPS375-21|Chthonius aff. occultus|CROBD772|Chthonius aff. occultus| CROPS376-21|Chthonius aff. occultus|CROBD773|Chthonius aff. occultus|
Group 56 n: 1; id: CROPS396-22|Roncus italicus|CROBD1099|Roncus italicus|
Group 57 n: 3; id: CROPS397-22|Roncus italicus|CROBD1100|Roncus italicus| CROPS400-22|Roncus italicus|CROBD1103|Roncus italicus| CROPS403-22|Roncus italicus|CROBD1106|Roncus italicus|
Group 58 n: 1; id: CROPS406-22|Chthonius ischnocheles reductus|CROBD1109|Chthonius ischnocheles|
Group 59 n: 2; id: CROPS407-22|Chthonius ischnocheles ischnocheles|CROBD1110|Chthonius ischnocheles| CROPS409-22|Chthonius ischnocheles ischnocheles|CROBD1114|Chthonius ischnocheles|
Group 60 n: 1; id: CROPS408-22|Chthonius sp. ZB|CROBD1113|Chthonius sp. ZB|
Group 61 n: 2; id: CROPS411-22|Roncus aff. ragusae|CROBD1117|Roncus aff. ragusae| CROPS415-22|Roncus aff. ragusae|CROBD1123|Roncus aff. ragusae|
Group 62 n: 2; id: CROPS413-22|Roncus insularis|CROBD1121|Roncus insularis| CROPS414-22|Roncus insularis|CROBD1122|Roncus insularis|
Group 63 n: 1; id: CROPS001-21|Roncus lubricus|CROBD382|Roncus lubricus| Archaeoroncus dalmatinus
Group 64 n: 1; id: CROPS004-21|Chthoniidae|CROBD386||Globochthonius | Globochthonius sp. n. B
Group 65 n: 2; id: CROPS006-21|Neobisium peruni|CROBD388|Neobisium peruni|Subgenus: Blothrus CROPS019-21|Neobisium peruni|CROBD405|Neobisium peruni|Subgenus: Blothrus
Group 66 n: 1; id: CROPS010-21|Chthoniidae|CROBD396||Globochthonius | Globochthonius sp. n. B
Group 67 n: 11; id: CROPS012-21|Chthonius aff. occultus|CROBD398|Chthonius aff. occultus| CROPS014-21|Chthonius aff. occultus|CROBD400|Chthonius aff. occultus| CROPS218-21|Chthonius aff. occultus|CROBD247|Chthonius aff. occultus| CROPS220-21|Chthonius aff. occultus|CROBD249|Chthonius aff. occultus| CROPS221-21|Chthonius aff. occultus|CROBD250|Chthonius aff. occultus| CROPS223-21|Chthonius aff. occultus|CROBD252|Chthonius aff. occultus| CROPS224-21|Chthonius aff. occultus|CROBD253|Chthonius aff. occultus| CROPS225-21|Chthonius aff. occultus|CROBD254|Chthonius aff. occultus| CROPS334-21|Chthonius aff. occultus|CROBD655|Chthonius aff. occultus| CROPS340-21|Chthonius aff. occultus|CROBD661|Chthonius aff. occultus| CROPS341-21|Chthonius aff. occultus|CROBD662|Chthonius aff. occultus|
Group 68 n: 1; id: CROPS016-21|Troglochthonius aff. mirabilis|CROBD402|Troglochthonius aff. mirabilis|
Group 69 n: 1; id: CROPS021-21|Roncus ragusae|CROBD410|Roncus ragusae|
Group 70 n: 1; id: CROPS022-21|Troglochthonius mirabilis|CROBD411|Troglochthonius mirabilis|
Group 71 n: 3; id: CROPS025-21|Chthonius subterraneus subterraneus|CROBD417|Chthonius subterraneus| CROPS026-21|Chthonius subterraneus subterraneus|CROBD418|Chthonius subterraneus| CROPS161-21|Chthonius subterraneus subterraneus|CROBD862|Chthonius subterraneus|
Group 72 n: 1; id: CROPS027-21|Neobisium heros|CROBD419|Neobisium heros|Subgenus: Blothrus
Group 73 n: 1; id: CROPS029-21|Chthoniidae|CROBD423||Ephippiochthonius | Ephippiochthonius insularis
Group 74 n: 1; id: CROPS030-21|Insulocreagris sp. n. M|CROBD424|Insulocreagris sp. n. M|
Group 75 n: 2; id: CROPS031-21|Roncus sp. n. P|CROBD426|Roncus sp. n. P| CROPS032-21|Roncus sp. n. P|CROBD427|Roncus sp. n. P|
Group 76 n: 1; id: CROPS036-21|Chthoniidae|CROBD437||Globochthonius | Globochthonius caligatus
Group 77 n: 1; id: CROPS037-21|Neobisium aff. heros|CROBD439|Neobisium aff. heros|
Group 78 n: 1; id: CROPS038-21|Occidenchthonius sp. PEL|CROBD442|Occidenchthonius sp. PEL|
Group 79 n: 1; id: CROPS039-21|Roncus anophthalmus|CROBD443|Roncus anophthalmus|
Group 80 n: 1; id: CROPS041-21|Roncus sp. 3DH|CROBD554|Roncus sp. 3DH|
Group 81 n: 1; id: CROPS042-21|Chthoniidae|CROBD555||Ephippiochthonius | Ephippiochthonius sp. 1DH
Group 82 n: 1; id: CROPS043-21|Chthoniidae|CROBD557||Ephippiochthonius | Ephippiochthonius insularis

Group 83 n: 1; id: CROPS049-21|Chthonius sp. nov. V|CROBD563|Chthonius sp. nov. V|
Group 84 n: 2; id: CROPS050-21|Chthonius aff. radjai|CROBD564|Chthonius aff. radjai| CROPS162-21|Chthonius aff. radjai|CROBD863|Chthonius aff. radjai|
Group 85 n: 5; id: CROPS051-21|Neobisium aff. svetovidi|CROBD565|Neobisium aff. svetovidi|Subgenus: Blothrus CROPS078-21|Neobisium aff. svetovidi|CROBD597|Neobisium aff. svetovidi|Subgenus: Blothrus CROPS089-21|Neobisium aff. svetovidi|CROBD610|Neobisium aff. svetovidi|Subgenus: Blothrus CROPS271-21|Neobisium aff. svetovidi|CROBD334|Neobisium aff. svetovidi|Subgenus: Blothrus CROPS321-21|Neobisium aff. svetovidi|CROBD623|Neobisium aff. svetovidi|Subgenus: Blothrus
Group 86 n: 4; id: CROPS054-21|Chthonius aff. absoloni|CROBD568|Chthonius aff. absoloni| CROPS056-21|Chthonius aff. absoloni|CROBD570|Chthonius aff. absoloni| CROPS071-21|Chthonius aff. absoloni|CROBD587|Chthonius aff. absoloni| CROPS084-21|Chthonius sp. nov. P|CROBD604|Chthonius sp. nov. P|
Group 87 n: 3; id: CROPS058-21|Insulocreagris sp. nov. P1|CROBD573|Insulocreagris sp. nov. P1| CROPS081-21|Insulocreagris sp. nov. P1|CROBD600|Insulocreagris sp. nov. P1| CROPS091-21|Insulocreagris sp. nov. P1|CROBD612|Insulocreagris sp. nov. P1|
Group 88 n: 1; id: CROPS060-21|Insulocreagris sp. 3DH|CROBD575|Insulocreagris sp. 3DH|
Group 89 n: 1; id: CROPS061-21|Neobisium elegans|CROBD576|Neobisium elegans|Subgenus: Neobisium
Group 90 n: 5; id: CROPS062-21|Chthonius sp. nov. PLJ|CROBD577|Chthonius sp. nov. PLJ| CROPS090-21|Chthonius sp. nov. PLJ|CROBD611|Chthonius sp. nov. PLJ| CROPS228-21|Chthonius aff. absoloni|CROBD258|Chthonius aff. absoloni| CROPS369-21|Chthonius sp. nov. PLJ|CROBD764|Chthonius sp. nov. PLJ| CROPS379-21|Chthonius sp. nov. PLJ|CROBD778|Chthonius sp. nov. PLJ|
Group 91 n: 3; id: CROPS073-21|Roncus lubricus|CROBD589|Roncus lubricus| CROPS377-21|Roncus lubricus|CROBD776|Roncus lubricus| CROPS378-21|Roncus lubricus|CROBD777|Roncus lubricus|
Group 92 n: 3; id: CROPS075-21|Neobisium aff. dalmatinum|CROBD591|Neobisium aff. dalmatinum|Subgenus: Blothrus CROPS079-21|Neobisium aff. dalmatinum|CROBD598|Neobisium aff. dalmatinum|Subgenus: Blothrus CROPS082-21|Neobisium aff. dalmatinum|CROBD601|Neobisium aff. dalmatinum|Subgenus: Blothrus
Group 93 n: 1; id: CROPS076-21|Insulocreagris sp. nov. P2|CROBD592|Insulocreagris sp. nov. P2|
Group 94 n: 1; id: CROPS094-21|Roncus narentae|CROBD616|Roncus narentae|
Group 95 n: 3; id: CROPS096-21|Chthonius aff. absoloni|CROBD618|Chthonius aff. absoloni| CROPS232-21|Chthonius aff. absoloni|CROBD262|Chthonius aff. absoloni| CROPS233-21|Chthonius aff. absoloni|CROBD263|Chthonius aff. absoloni|
Group 96 n: 1; id: CROPS102-21|Chthonius sp. LK|CROBD680|Chthonius sp. LK|
Group 97 n: 5; id: CROPS109-21|Chthonius subterraneus meuseli|CROBD688|Chthonius subterraneus| CROPS115-21|Chthonius subterraneus meuseli|CROBD696|Chthonius subterraneus| CROPS121-21|Chthonius subterraneus meuseli|CROBD740|Chthonius subterraneus| CROPS122-21|Chthonius subterraneus meuseli|CROBD741|Chthonius subterraneus| CROPS124-21|Chthonius subterraneus meuseli|CROBD743|Chthonius subterraneus|
Group 98 n: 1; id: CROPS137-21|Chthoniidae|CROBD813||Globochthonius | Globochthonius sp. nov. DO
Group 99 n: 2; id: CROPS138-21|Roncus aff. lubricus 3|CROBD815|Roncus aff. lubricus 3| CROPS247-21|Roncus aff. lubricus 3|CROBD289|Roncus aff. lubricus 3|
Group 100 n: 1; id: CROPS141-21|Neobisium gentile flavum|CROBD818|Neobisium gentile|Subgenus: Neobisium
Group 101 n: 1; id: CROPS147-21|Neobisium aff. reimoseri|CROBD828|Neobisium aff. reimoseri|Subgenus: Blothrus
Group 102 n: 1; id: CROPS151-21|Chthoniidae|CROBD834||Globochthonius | Globochthonius aff. caligatus
Group 103 n: 1; id: CROPS152-21|Neobisium sp. nov. KP|CROBD835|Neobisium sp. nov. KP|
Group 104 n: 1; id: CROPS153-21|Insulocreagris sp. n. M|CROBD837|Insulocreagris sp. n. M|
Group 105 n: 1; id: CROPS156-21|Neobisium gentile flavum|CROBD844|Neobisium gentile|Subgenus: Neobisium
Group 106 n: 1; id: CROPS160-21|Roncus lubricus|CROBD851|Roncus lubricus|
Group 107 n: 2; id: CROPS164-21|Chthonius sp. nov. D|CROBD868|Chthonius sp. nov. D| CROPS347-21|Chthonius sp. nov. KO|CROBD669|Chthonius sp. nov. KO|
Group 108 n: 1; id: CROPS172-21|Neobisium gentile flavum|CROBD888|Neobisium gentile|Subgenus: Neobisium
Group 109 n: 1; id: CROPS173-21|Neobisium vjetrenicae|CROBD889|Neobisium vjetrenicae|Subgenus: Blothrus
Group 110 n: 1; id: CROPS174-21|Roncus aff. insularis|CROBD904|Roncus aff. insularis|
Group 111 n: 1; id: CROPS175-21|Roncus lubricus|CROBD906|Roncus lubricus|Archaeoroncus dalmatinus
Group 112 n: 1; id: CROPS176-21|Chthoniidae|CROBD907||Microchthonius | Microchthonius elegantissimus
Group 113 n: 3; id: CROPS180-21|Chthonius sp. nov. VL|CROBD921|Chthonius sp. nov. VL| CROPS181-21|Chthonius sp. nov. VL|CROBD922|Chthonius sp. nov. VL| CROPS290-21|Chthonius sp. nov. VL|CROBD368|Chthonius sp. nov. VL|
Group 114 n: 3; id: CROPS183-21|Neobisium stribogi|CROBD201|Neobisium stribogi|Subgenus: Pennobisium CROPS192-21|Neobisium stribogi|CROBD213|Neobisium stribogi|Subgenus: Pennobisium CROPS193-21|Neobisium stribogi|CROBD215|Neobisium stribogi|Subgenus: Pennobisium
Group 115 n: 2; id: CROPS184-21|Roncus lubricus|CROBD202|Roncus lubricus|Archaeoroncus dalmatinus CROPS206-21|Roncus lubricus|CROBD232|Roncus lubricus|Archaeoroncus dalmatinus
Group 116 n: 2; id: CROPS186-21|Insulocreagris sp. 1DH|CROBD204|Insulocreagris sp. 1DH| CROPS302-21|Insulocreagris sp. 1DH|CROBD521|Insulocreagris sp. 1DH|
Group 117 n: 2; id: CROPS187-21|Lasiochernes|CROBD205||sp. 1DH CROPS248-21|Lasiochernes|CROBD290||sp. 1DH

Group 118 n: 2; id: CROPS191-21|Neobisium sp. nov. M|CROBD212|Neobisium sp. nov. M| CROPS353-21|Neobisium sp. nov. M|CROBD700|Neobisium sp. nov. M|
Group 119 n: 2; id: CROPS194-21|Neobisium carcinoides|CROBD216|Neobisium carcinoides|Subgenus: Neobisium CROPS296-21|Neobisium carcinoides|CROBD378|Neobisium carcinoides|Subgenus: Neobisium
Group 120 n: 3; id: CROPS195-21|Neobisium reimoseri|CROBD217|Neobisium reimoseri|Subgenus: Blothrus CROPS337-21|Neobisium reimoseri|CROBD658|Neobisium reimoseri|Subgenus: Blothrus CROPS338-21|Neobisium reimoseri|CROBD659|Neobisium reimoseri|Subgenus: Blothrus
Group 121 n: 1; id: CROPS196-21|Roncus sp. 2DH|CROBD218|Roncus sp. 2DH|
Group 122 n: 1; id: CROPS202-21|Chthonius sp. 1DH|CROBD225|Chthonius sp. 1DH|
Group 123 n: 3; id: CROPS203-21|Roncus sp. nov. G|CROBD229|Roncus sp. nov. G| CROPS204-21|Roncus sp. nov. G|CROBD230|Roncus sp. nov. G| CROPS205-21|Roncus sp. nov. G|CROBD231|Roncus sp. nov. G|
Group 124 n: 2; id: CROPS211-21|Roncus podaga|CROBD237|Roncus podaga| CROPS213-21|Roncus podaga|CROBD242|Roncus podaga|
Group 125 n: 3; id: CROPS212-21|Chthonius aff. alpicola|CROBD241|Chthonius aff. alpicola| CROPS214-21|Chthonius aff. alpicola|CROBD243|Chthonius aff. alpicola| CROPS245-21|Chthonius aff. alpicola|CROBD283|Chthonius aff. alpicola|
Group 126 n: 1; id: CROPS215-21|Chthonius sp. 2DH|CROBD244|Chthonius sp. 2DH|
Group 127 n: 2; id: CROPS226-21|Neobisium aff. elegans|CROBD256|Neobisium aff. elegans|Subgenus: Neobisium CROPS330-21|Neobisium aff. elegans|CROBD645|Neobisium aff. elegans|Subgenus: Neobisium
Group 128 n: 1; id: CROPS231-21|Roncus aff. lubricus|CROBD261|Roncus aff. lubricus|
Group 129 n: 1; id: CROPS234-21|Neobisium gentile alternum|CROBD267|Neobisium gentile|Subgenus: Neobisium
Group 130 n: 3; id: CROPS239-21|Insulocreagris sp. TS|CROBD273|Insulocreagris sp. TS| CROPS240-21|Insulocreagris sp. TS|CROBD274|Insulocreagris sp. TS| CROPS241-21|Insulocreagris sp. TS|CROBD275|Insulocreagris sp. TS|
Group 131 n: 1; id: CROPS243-21|Roncus aff. lubricus|CROBD277|Roncus aff. lubricus|
Group 132 n: 1; id: CROPS250-21|Neobisium reimoseri|CROBD294|Neobisium reimoseri|Subgenus: Blothrus
Group 133 n: 2; id: CROPS251-21|Chthoniidae|CROBD298||Ephippiochthonius | Ephippiochthonius insularis CROPS252-21|Chthoniidae|CROBD300||Ephippiochthonius | Ephippiochthonius insularis
Group 134 n: 2; id: CROPS258-21|Chthonius sp. nov. M2|CROBD310|Chthonius sp. nov. M2| CROPS260-21|Chthonius sp. nov. M2|CROBD315|Chthonius sp. nov. M2|
Group 135 n: 2; id: CROPS264-21|Roncus anophthalmus|CROBD325|Roncus anophthalmus| CROPS273-21|Roncus anophthalmus|CROBD337|Roncus anophthalmus|
Group 136 n: 1; id: CROPS284-21|Chthoniidae|CROBD355||Ephippiochthonius | Ephippiochthonius tetrachelatus
Group 137 n: 4; id: CROPS285-21|Chthonius ischnocheles|CROBD358|Chthonius ischnocheles| CROPS323-21|Chthonius ischnocheles|CROBD635|Chthonius ischnocheles| CROPS325-21|Chthonius ischnocheles|CROBD637|Chthonius ischnocheles| CROPS326-21|Chthonius ischnocheles|CROBD638|Chthonius ischnocheles|
Group 138 n: 4; id: CROPS286-21|Chthoniidae|CROBD359||Ephippiochthonius | Ephippiochthonius tetrachelatus CROPS287-21|Chthoniidae|CROBD362||Ephippiochthonius | Ephippiochthonius tetrachelatus CROPS288-21|Chthoniidae|CROBD364||Ephippiochthonius | Ephippiochthonius tetrachelatus CROPS289-21|Chthoniidae|CROBD365||Ephippiochthonius | Ephippiochthonius tetrachelatus
Group 139 n: 2; id: CROPS292-21|Neobisium sp. nov. M|CROBD371|Neobisium sp. nov. M| CROPS293-21|Neobisium sp. nov. M|CROBD372|Neobisium sp. nov. M|
Group 140 n: 3; id: CROPS294-21|Roncus sp. 1DH|CROBD376|Roncus sp. 1DH| CROPS297-21|Roncus sp. 1DH|CROBD379|Roncus sp. 1DH| CROPS298-21|Roncus sp. 1DH|CROBD380|Roncus sp. 1DH|
Group 141 n: 1; id: CROPS295-21|Roncus aff. italicus|CROBD377|Roncus aff. italicus|
Group 142 n: 1; id: CROPS301-21|Garypus sp. nov. M|CROBD519|Garypus sp. nov. M|
Group 143 n: 2; id: CROPS305-21|Roncus sp. nov. S|CROBD525|Roncus sp. nov. S| CROPS306-21|Roncus sp. nov. S|CROBD526|Roncus sp. nov. S|
Group 144 n: 1; id: CROPS319-21|Neobisium sp. nov. M|CROBD621|Neobisium sp. nov. M|
Group 145 n: 1; id: CROPS324-21|Insulocreagris sp. 3DH|CROBD636|Insulocreagris sp. 3DH|
Group 146 n: 2; id: CROPS328-21|Rhacochelifer maculatus|CROBD641|Rhacochelifer maculatus| CROPS331-21|Rhacochelifer maculatus|CROBD646|Rhacochelifer maculatus|
Group 147 n: 1; id: CROPS329-21|Chthonius aff. heterodactylus|CROBD643|Chthonius aff. heterodactylus|
Group 148 n: 1; id: CROPS342-21|Neobisium fuscimanum|CROBD663|Neobisium fuscimanum|Subgenus: Neobisium
Group 149 n: 2; id: CROPS343-21|Neobisium reimoseri|CROBD664|Neobisium reimoseri|Subgenus: Blothrus CROPS346-21|Neobisium reimoseri|CROBD667|Neobisium reimoseri|Subgenus: Blothrus
Group 150 n: 1; id: CROPS357-21|Roncus aff. lubricus|CROBD750|Roncus aff. lubricus|
Group 151 n: 1; id: CROPS360-21|Neobisium reimoseri|CROBD754|Neobisium reimoseri|Subgenus: Blothrus
Group 152 n: 1; id: CROPS367-21|Roncus aff. ragusae|CROBD762|Roncus aff. ragusae|
Group 153 n: 1; id: CROPS370-21|Chthoniidae|CROBD766||Occidenchthonius | Occidenchthonius sp. nov. PR
Group 154 n: 1; id: CROPS372-21|Chthoniidae|CROBD768||Ephippiochthonius | Ephippiochthonius aff. insularis
Group 155 n: 1; id: CROPS374-21|Roncus aff. lubricus|CROBD771|Roncus aff. lubricus|
Group 156 n: 2; id: CROPS479-22|Chthonius sp. nov. PL|CROBD1161|Chthonius sp. nov. PL| CROPS480-22|Chthonius sp. nov. PL|CROBD1162|Chthonius sp. nov. PL|

Group 157 n: 4; id: CROPS066-21|Insulocreagris sp. nov. PI|CROBD582|Insulocreagris sp. nov. PI| CROPS068-21|Insulocreagris sp. nov. PI|CROBD584|Insulocreagris sp. nov. PI| CROPS092-21|Insulocreagris sp. nov. PI|CROBD613|Insulocreagris sp. nov. PI| CROPS093-21|Insulocreagris sp. nov. PI|CROBD614|Insulocreagris sp. nov. PI|
Group 158 n: 1; id: CROPS166-21|Neobisium gentile gentile|CROBD871|Neobisium gentile|Subgenus: Neobisium
Group 159 n: 6; id: CROPS197-21|Chthonius aff. occultus|CROBD220|Chthonius aff. occultus| CROPS198-21|Chthonius aff. occultus|CROBD221|Chthonius aff. occultus| CROPS199-21|Chthonius aff. occultus|CROBD222|Chthonius aff. occultus| CROPS200-21|Chthonius aff. occultus|CROBD223|Chthonius aff. occultus| CROPS201-21|Chthonius aff. occultus|CROBD224|Chthonius aff. occultus| CROPS235-21|Chthonius aff. occultus|CROBD268|Chthonius aff. occultus|

ASAP

Group 1 n: 9; id: CROPS417-22|Neobisium insulare|CROBD286|Neobisium insulare|Subgenus: Blothrus CROPS185-21|Neobisium insulare|CROBD203|Neobisium insulare|Subgenus: Blothrus CROPS209-21|Neobisium insulare|CROBD235|Neobisium insulare|Subgenus: Blothrus CROPS210-21|Neobisium insulare|CROBD236|Neobisium insulare|Subgenus: Blothrus CROPS227-21|Neobisium insulare|CROBD257|Neobisium insulare|Subgenus: Blothrus CROPS230-21|Neobisium insulare|CROBD260|Neobisium insulare|Subgenus: Blothrus CROPS255-21|Neobisium insulare|CROBD303|Neobisium insulare|Subgenus: Blothrus CROPS003-21|Neobisium insulare|CROBD385|Neobisium insulare|Subgenus: Blothrus CROPS020-21|Neobisium insulare|CROBD408|Neobisium insulare|Subgenus: Blothrus
Group 2 n: 10; id: CROPS418-22|Neobisium staudacheri|CROBD407|Neobisium staudacheri|Subgenus: Ommatoblothrus CROPS005-21|Neobisium staudacheri|CROBD387|Neobisium staudacheri|Subgenus: Ommatoblothrus CROPS445-22|Protoneobisium biocovense|CROBD898|Protoneobisium biocovense| CROPS446-22|Protoneobisium biocovense|CROBD899|Protoneobisium biocovense| CROPS008-21|Protoneobisium biocovense|CROBD390|Protoneobisium biocovense| CROPS291-21|Protoneobisium biocovense|CROBD370|Protoneobisium biocovense| CROPS452-22|Protoneobisium biocovense|CROBD911|Protoneobisium biocovense| CROPS454-22|Protoneobisium biocovense|CROBD913|Protoneobisium biocovense| CROPS456-22|Protoneobisium biocovense|CROBD917|Protoneobisium biocovense| CROPS009-21|Protoneobisium biocovense|CROBD393|Protoneobisium biocovense|
Group 3 n: 1; id: CROPS419-22|Neobisium aff. vachoni|CROBD413|Neobisium aff. vachoni|Subgenus: Blothrus
Group 4 n: 48; id: CROPS420-22|Neobisium stygium|CROBD624|Neobisium stygium|Subgenus: Blothrus CROPS048-21|Neobisium stygium|CROBD562|Neobisium stygium|Subgenus: Blothrus CROPS087-21|Neobisium stygium|CROBD608|Neobisium stygium|Subgenus: Blothrus CROPS272-21|Neobisium stygium|CROBD335|Neobisium stygium|Subgenus: Blothrus CROPS053-21|Neobisium stygium|CROBD567|Neobisium stygium|Subgenus: Blothrus CROPS055-21|Neobisium stygium|CROBD569|Neobisium stygium|Subgenus: Blothrus CROPS088-21|Neobisium stygium|CROBD609|Neobisium stygium|Subgenus: Blothrus CROPS163-21|Neobisium stygium|CROBD864|Neobisium stygium|Subgenus: Blothrus CROPS188-21|Neobisium stygium|CROBD207|Neobisium stygium|Subgenus: Blothrus CROPS190-21|Neobisium stygium|CROBD209|Neobisium stygium|Subgenus: Blothrus CROPS270-21|Neobisium stygium|CROBD332|Neobisium stygium|Subgenus: Blothrus CROPS274-21|Neobisium stygium|CROBD339|Neobisium stygium|Subgenus: Blothrus CROPS279-21|Neobisium stygium|CROBD347|Neobisium stygium|Subgenus: Blothrus CROPS052-21|Neobisium stygium|CROBD566|Neobisium stygium|Subgenus: Blothrus CROPS069-21|Neobisium stygium|CROBD585|Neobisium stygium|Subgenus: Blothrus CROPS131-21|Neobisium stygium|CROBD797|Neobisium stygium|Subgenus: Blothrus CROPS177-21|Neobisium stygium|CROBD915|Neobisium stygium|Subgenus: Blothrus CROPS063-21|Neobisium stygium|CROBD578|Neobisium stygium|Subgenus: Blothrus CROPS064-21|Neobisium stygium|CROBD580|Neobisium stygium|Subgenus: Blothrus CROPS077-21|Neobisium stygium|CROBD593|Neobisium stygium|Subgenus: Blothrus CROPS128-21|Neobisium stygium|CROBD783|Neobisium stygium|Subgenus: Blothrus CROPS065-21|Neobisium stygium|CROBD581|Neobisium stygium|Subgenus: Blothrus CROPS130-21|Neobisium stygium|CROBD787|Neobisium stygium|Subgenus: Blothrus CROPS133-21|Neobisium stygium|CROBD799|Neobisium stygium|Subgenus: Blothrus CROPS361-21|Neobisium stygium|CROBD756|Neobisium stygium|Subgenus: Blothrus CROPS362-21|Neobisium stygium|CROBD757|Neobisium stygium|Subgenus: Blothrus CROPS363-21|Neobisium stygium|CROBD758|Neobisium stygium|Subgenus: Blothrus CROPS103-21|Neobisium stygium|CROBD681|Neobisium stygium|Subgenus: Blothrus CROPS108-21|Neobisium stygium|CROBD687|Neobisium stygium|Subgenus: Blothrus CROPS125-21|Neobisium stygium|CROBD744|Neobisium stygium|Subgenus: Blothrus CROPS266-21|Neobisium stygium|CROBD328|Neobisium stygium|Subgenus: Blothrus CROPS281-21|Neobisium stygium|CROBD351|Neobisium stygium|Subgenus: Blothrus CROPS129-21|Neobisium stygium|CROBD786|Neobisium stygium|Subgenus: Blothrus CROPS380-21|Neobisium stygium|CROBD895|Neobisium stygium|Subgenus: Blothrus CROPS140-21|Neobisium stygium|CROBD817|Neobisium stygium|Subgenus: Blothrus CROPS155-21|Neobisium stygium|CROBD839|Neobisium stygium|Subgenus: Blothrus CROPS344-21|Neobisium stygium|CROBD665|Neobisium stygium|Subgenus: Blothrus CROPS345-21|Neobisium stygium|CROBD666|Neobisium stygium|Subgenus: Blothrus CROPS359-21|Neobisium stygium|CROBD753|Neobisium stygium|Subgenus: Blothrus CROPS145-21|Neobisium stygium|CROBD826|Neobisium stygium|Subgenus: Blothrus CROPS358-21|Neobisium stygium|CROBD752|Neobisium stygium|Subgenus: Blothrus CROPS249-21|Neobisium stygium|CROBD293|Neobisium stygium|Subgenus: Blothrus CROPS150-21|Neobisium stygium|CROBD832|Neobisium stygium|Subgenus: Blothrus CROPS170-21|Neobisium stygium|CROBD886|Neobisium stygium|Subgenus: Blothrus CROPS263-21|Neobisium aff. spelaeum|CROBD323|Neobisium aff. spelaeum|Subgenus:

Blothrus CROPS336-21|Neobisium aff. spelaeum|CROBD657|Neobisium aff. spelaeum|Subgenus: Blothrus CROPS275-21|Neobisium aff. spelaeum|CROBD342|Neobisium aff. spelaeum|Subgenus: Blothrus CROPS463-22|Neobisium stygium|CROBD1139|Neobisium stygium|Subgenus: Blothrus

Group 5 n: 4; id: CROPS421-22|Chernes hahnii|CROBD632|Chernes hahnii| CROPS317-21|Chernes hahnii|CROBD595|Chernes hahnii| CROPS300-21|Chernes hahnii|CROBD468|Chernes hahnii| CROPS316-21|Chernes hahnii|CROBD594|Chernes hahnii|

Group 6 n: 17; id: CROPS422-22|Chthonius absoloni|CROBD686|Chthonius absoloni| CROPS423-22|Chthonius absoloni|CROBD694|Chthonius absoloni| CROPS424-22|Chthonius absoloni|CROBD705|Chthonius absoloni| CROPS425-22|Chthonius absoloni|CROBD706|Chthonius absoloni| CROPS393-22|Chthonius absoloni|CROBD1022|Chthonius absoloni| CROPS394-22|Chthonius absoloni|CROBD1023|Chthonius absoloni| CROPS104-21|Chthonius absoloni|CROBD682|Chthonius absoloni| CROPS105-21|Chthonius absoloni|CROBD683|Chthonius absoloni| CROPS106-21|Chthonius absoloni|CROBD684|Chthonius absoloni| CROPS107-21|Chthonius absoloni|CROBD685|Chthonius absoloni| CROPS116-21|Chthonius absoloni|CROBD697|Chthonius absoloni| CROPS117-21|Chthonius absoloni|CROBD698|Chthonius absoloni| CROPS111-21|Chthonius absoloni|CROBD690|Chthonius absoloni| CROPS118-21|Chthonius absoloni|CROBD699|Chthonius absoloni| CROPS123-21|Chthonius absoloni|CROBD742|Chthonius absoloni| CROPS479-22|Chthonius sp. nov. PL|CROBD1161|Chthonius sp. nov. PL| CROPS480-22|Chthonius sp. nov. PL|CROBD1162|Chthonius sp. nov. PL|

Group 7 n: 4; id: CROPS426-22|Chthonius exarmatus|CROBD765|Chthonius exarmatus| CROPS044-21|Chthonius exarmatus|CROBD558|Chthonius exarmatus| CROPS045-21|Chthonius exarmatus|CROBD559|Chthonius exarmatus| CROPS238-21|Chthonius exarmatus|CROBD272|Chthonius exarmatus|

Group 8 n: 8; id: CROPS427-22|Neobisium gentile alternum|CROBD779|Neobisium gentile|Subgenus: Neobisium CROPS015-21|Neobisium gentile alternum|CROBD401|Neobisium gentile|Subgenus: Neobisium CROPS434-22|Neobisium gentile alternum|CROBD845|Neobisium gentile|Subgenus: Neobisium CROPS373-21|Neobisium gentile alternum|CROBD770|Neobisium gentile|Subgenus: Neobisium CROPS440-22|Neobisium gentile alternum|CROBD878|Neobisium gentile|Subgenus: Neobisium CROPS011-21|Neobisium gentile alternum|CROBD397|Neobisium gentile|Subgenus: Neobisium CROPS007-21|Neobisium gentile alternum|CROBD389|Neobisium gentile|Subgenus: Neobisium CROPS234-21|Neobisium gentile alternum|CROBD267|Neobisium gentile|Subgenus: Neobisium

Group 9 n: 1; id: CROPS428-22|Chthonius aff. subterraneus|CROBD800|Chthonius aff. subterraneus|Chthonius aff. subterraneus meuseli

Group 10 n: 1; id: CROPS429-22|Chthonius sp. nov. R|CROBD806|Chthonius sp. nov. R|

Group 11 n: 44; id: CROPS430-22|Neobisium stygium|CROBD809|Neobisium stygium|Subgenus: Blothrus CROPS392-22|Neobisium stygium|CROBD1021|Neobisium stygium|Subgenus: Blothrus CROPS099-21|Neobisium stygium|CROBD677|Neobisium stygium|Subgenus: Blothrus CROPS100-21|Neobisium stygium|CROBD678|Neobisium stygium|Subgenus: Blothrus CROPS110-21|Neobisium stygium|CROBD689|Neobisium stygium|Subgenus: Blothrus CROPS114-21|Neobisium stygium|CROBD695|Neobisium stygium|Subgenus: Blothrus CROPS127-21|Neobisium stygium|CROBD747|Neobisium stygium|Subgenus: Blothrus CROPS268-21|Neobisium stygium|CROBD330|Neobisium stygium|Subgenus: Blothrus CROPS303-21|Neobisium stygium|CROBD522|Neobisium stygium|Subgenus: Blothrus CROPS304-21|Neobisium stygium|CROBD524|Neobisium stygium|Subgenus: Blothrus CROPS320-21|Neobisium stygium|CROBD622|Neobisium stygium|Subgenus: Blothrus CROPS355-21|Neobisium stygium|CROBD748|Neobisium stygium|Subgenus: Blothrus CROPS097-21|Neobisium stygium|CROBD675|Neobisium stygium|Subgenus: Blothrus CROPS098-21|Neobisium stygium|CROBD676|Neobisium stygium|Subgenus: Blothrus CROPS126-21|Neobisium stygium|CROBD746|Neobisium stygium|Subgenus: Blothrus CROPS113-21|Neobisium stygium|CROBD693|Neobisium stygium|Subgenus: Blothrus CROPS112-21|Neobisium stygium|CROBD691|Neobisium stygium|Subgenus: Blothrus CROPS354-21|Neobisium stygium|CROBD703|Neobisium stygium|Subgenus: Blothrus CROPS267-21|Neobisium stygium|CROBD329|Neobisium stygium|Subgenus: Blothrus CROPS276-21|Neobisium stygium|CROBD343|Neobisium stygium|Subgenus: Blothrus CROPS277-21|Neobisium stygium|CROBD344|Neobisium stygium|Subgenus: Blothrus CROPS475-22|Neobisium stygium|CROBD1157|Neobisium stygium|Subgenus: Blothrus CROPS477-22|Neobisium stygium|CROBD1159|Neobisium stygium|Subgenus: Blothrus CROPS308-21|Neobisium stygium|CROBD528|Neobisium stygium|Subgenus: Blothrus CROPS310-21|Neobisium stygium|CROBD530|Neobisium stygium|Subgenus: Blothrus CROPS309-21|Neobisium stygium|CROBD529|Neobisium stygium|Subgenus: Blothrus CROPS448-22|Neobisium aff. spelaeum|CROBD902|Neobisium aff. spelaeum|Subgenus: Blothrus CROPS253-21|Neobisium aff. spelaeum|CROBD301|Neobisium aff. spelaeum|Subgenus: Blothrus CROPS254-21|Neobisium aff. spelaeum|CROBD302|Neobisium aff. spelaeum|Subgenus: Blothrus CROPS142-21|Neobisium aff. spelaeum|CROBD819|Neobisium aff. spelaeum|Subgenus: Blothrus CROPS149-21|Neobisium aff. spelaeum|CROBD831|Neobisium aff. spelaeum|Subgenus: Blothrus CROPS171-21|Neobisium aff. spelaeum|CROBD887|Neobisium aff. spelaeum|Subgenus: Blothrus CROPS158-21|Neobisium aff. spelaeum|CROBD847|Neobisium aff. spelaeum|Subgenus: Blothrus CROPS159-21|Neobisium aff. spelaeum|CROBD848|Neobisium aff. spelaeum|Subgenus: Blothrus CROPS404-22|Neobisium stygium|CROBD1107|Neobisium stygium|Subgenus: Blothrus CROPS405-22|Neobisium stygium|CROBD1108|Neobisium stygium|Subgenus: Blothrus CROPS398-22|Neobisium stygium|CROBD1101|Neobisium stygium|Subgenus: Blothrus CROPS136-21|Neobisium stygium|CROBD810|Neobisium stygium|Subgenus: Blothrus CROPS143-21|Neobisium stygium|CROBD823|Neobisium stygium|Subgenus: Blothrus CROPS311-21|Neobisium stygium|CROBD531|Neobisium stygium|Subgenus: Blothrus CROPS313-21|Neobisium stygium|CROBD533|Neobisium stygium|Subgenus: Blothrus CROPS314-21|Neobisium stygium|CROBD534|Neobisium stygium|Subgenus: Blothrus CROPS315-21|Neobisium stygium|CROBD535|Neobisium stygium|Subgenus: Blothrus CROPS312-21|Neobisium stygium|CROBD532|Neobisium stygium|Subgenus: Blothrus

Group 12 n: 5; id: CROPS431-22|Insulocreagris sp. nov. PI|CROBD820|Insulocreagris sp. nov. PI| CROPS066-21|Insulocreagris sp. nov. PI|CROBD582|Insulocreagris sp. nov. PI| CROPS092-21|Insulocreagris sp. nov. PI|CROBD613|Insulocreagris sp. nov. PI| CROPS093-21|Insulocreagris sp. nov. PI|CROBD614|Insulocreagris sp. nov. PI| CROPS068-21|Insulocreagris sp. nov. PI|CROBD584|Insulocreagris sp. nov. PI|
Group 13 n: 1; id: CROPS432-22|Insulocreagris sp. 2DH|CROBD830|Insulocreagris sp. 2DH|
Group 14 n: 6; id: CROPS433-22|Chthoniidae|CROBD841||Globochthonius | Globochthonius caligatus CROPS436-22|Chthoniidae|CROBD866||Globochthonius | Globochthonius caligatus CROPS135-21|Chthoniidae|CROBD807||Globochthonius | Globochthonius caligatus CROPS229-21|Chthoniidae|CROBD259||Globochthonius | Globochthonius caligatus CROPS391-22|Chthoniidae|CROBD1020||Globochthonius | Globochthonius caligatus CROPS364-21|Chthoniidae|CROBD759||Globochthonius | Globochthonius caligatus
Group 15 n: 2; id: CROPS435-22|Chthonius sp. nov. L|CROBD865|Chthonius sp. nov. L| CROPS101-21|Chthonius sp. nov. L|CROBD679|Chthonius sp. nov. L|
Group 16 n: 6; id: CROPS437-22|Neobisium gentile gentile|CROBD872|Neobisium gentile|Subgenus: Neobisium CROPS167-21|Neobisium gentile gentile|CROBD873|Neobisium gentile|Subgenus: Neobisium CROPS485-22|Neobisium gentile gentile|CROBD1167|Neobisium gentile|Subgenus: Neobisium | Neobisium aff. gentile gentile CROPS486-22|Neobisium gentile gentile|CROBD1168|Neobisium gentile|Subgenus: Neobisium | Neobisium aff. gentile gentile CROPS146-21|Neobisium gentile gentile|CROBD827|Neobisium gentile|Subgenus: Neobisium | Neobisium aff. gentile gentile CROPS166-21|Neobisium gentile gentile|CROBD871|Neobisium gentile|Subgenus: Neobisium
Group 17 n: 1; id: CROPS438-22|Chthoniidae|CROBD875||Globochthonius | Globochthonius sp. nov. S
Group 18 n: 6; id: CROPS439-22|Chthonius trebinjensis|CROBD876|Chthonius trebinjensis| CROPS494-22|Chthonius trebinjensis|CROBD1180|Chthonius trebinjensis| CROPS332-21|Chthonius trebinjensis|CROBD653|Chthonius trebinjensis| CROPS034-21|Chthonius trebinjensis|CROBD435|Chthonius trebinjensis| CROPS040-21|Chthonius trebinjensis|CROBD444|Chthonius trebinjensis| CROPS265-21|Chthonius trebinjensis|CROBD327|Chthonius trebinjensis|
Group 19 n: 1; id: CROPS441-22|Neobisium heros|CROBD880|Neobisium heros|Subgenus: Blothrus
Group 20 n: 7; id: CROPS442-22|Chthoniidae|CROBD885||Ephippiochthonius | Ephippiochthonius tetrachelatus CROPS282-21|Chthoniidae|CROBD353||Ephippiochthonius | Ephippiochthonius tetrachelatus CROPS283-21|Chthoniidae|CROBD354||Ephippiochthonius | Ephippiochthonius tetrachelatus CROPS286-21|Chthoniidae|CROBD359||Ephippiochthonius | Ephippiochthonius tetrachelatus CROPS287-21|Chthoniidae|CROBD362||Ephippiochthonius | Ephippiochthonius tetrachelatus CROPS288-21|Chthoniidae|CROBD364||Ephippiochthonius | Ephippiochthonius tetrachelatus CROPS289-21|Chthoniidae|CROBD365||Ephippiochthonius | Ephippiochthonius tetrachelatus
Group 21 n: 6; id: CROPS443-22|Neobisium gentile flavum|CROBD891|Neobisium gentile|Subgenus: Neobisium CROPS444-22|Neobisium gentile flavum|CROBD893|Neobisium gentile|Subgenus: Neobisium CROPS148-21|Neobisium gentile flavum|CROBD829|Neobisium gentile|Subgenus: Neobisium CROPS172-21|Neobisium gentile flavum|CROBD888|Neobisium gentile|Subgenus: Neobisium CROPS141-21|Neobisium gentile flavum|CROBD818|Neobisium gentile|Subgenus: Neobisium CROPS156-21|Neobisium gentile flavum|CROBD844|Neobisium gentile|Subgenus: Neobisium
Group 22 n: 24; id: CROPS447-22|Chthonius aff. occultus|CROBD900|Chthonius aff. occultus| CROPS449-22|Chthonius aff. occultus|CROBD905|Chthonius aff. occultus| CROPS002-21|Chthonius aff. occultus|CROBD384|Chthonius aff. occultus| CROPS395-22|Chthonius aff. occultus|CROBD1081|Chthonius aff. occultus| CROPS375-21|Chthonius aff. occultus|CROBD772|Chthonius aff. occultus| CROPS376-21|Chthonius aff. occultus|CROBD773|Chthonius aff. occultus| CROPS246-21|Chthonius aff. occultus|CROBD284|Chthonius aff. occultus| CROPS197-21|Chthonius aff. occultus|CROBD220|Chthonius aff. occultus| CROPS200-21|Chthonius aff. occultus|CROBD223|Chthonius aff. occultus| CROPS235-21|Chthonius aff. occultus|CROBD268|Chthonius aff. occultus| CROPS198-21|Chthonius aff. occultus|CROBD221|Chthonius aff. occultus| CROPS199-21|Chthonius aff. occultus|CROBD222|Chthonius aff. occultus| CROPS201-21|Chthonius aff. occultus|CROBD224|Chthonius aff. occultus| CROPS012-21|Chthonius aff. occultus|CROBD398|Chthonius aff. occultus| CROPS014-21|Chthonius aff. occultus|CROBD400|Chthonius aff. occultus| CROPS334-21|Chthonius aff. occultus|CROBD655|Chthonius aff. occultus| CROPS218-21|Chthonius aff. occultus|CROBD247|Chthonius aff. occultus| CROPS220-21|Chthonius aff. occultus|CROBD249|Chthonius aff. occultus| CROPS221-21|Chthonius aff. occultus|CROBD250|Chthonius aff. occultus| CROPS223-21|Chthonius aff. occultus|CROBD252|Chthonius aff. occultus| CROPS224-21|Chthonius aff. occultus|CROBD253|Chthonius aff. occultus| CROPS225-21|Chthonius aff. occultus|CROBD254|Chthonius aff. occultus| CROPS340-21|Chthonius aff. occultus|CROBD661|Chthonius aff. occultus| CROPS341-21|Chthonius aff. occultus|CROBD662|Chthonius aff. occultus|
Group 23 n: 3; id: CROPS450-22|Chthoniidae|CROBD909||Globochthonius | Globochthonius sp. nov. B CROPS004-21|Chthoniidae|CROBD386||Globochthonius | Globochthonius sp. n. B CROPS010-21|Chthoniidae|CROBD396||Globochthonius | Globochthonius sp. n. B
Group 24 n: 1; id: CROPS451-22|Roncus aff. lubricus 2|CROBD910|Roncus aff. lubricus 2|
Group 25 n: 4; id: CROPS453-22|Neobisium maderi|CROBD912|Neobisium maderi|Subgenus: Blothrus CROPS178-21|Neobisium maderi|CROBD919|Neobisium maderi|Subgenus: Blothrus CROPS381-21|Neobisium maderi|CROBD901|Neobisium maderi|Subgenus: Blothrus CROPS455-22|Neobisium maderi|CROBD916|Neobisium maderi|Subgenus: Blothrus
Group 26 n: 11; id: CROPS457-22|Neobisium tantaleum|CROBD918|Neobisium tantaleum|Subgenus: Blothrus CROPS182-21|Neobisium tantaleum|CROBD923|Neobisium tantaleum|Subgenus: Blothrus CROPS217-21|Neobisium tantaleum|CROBD246|Neobisium tantaleum|Subgenus: Blothrus CROPS222-21|Neobisium tantaleum|CROBD251|Neobisium tantaleum|Subgenus: Blothrus CROPS333-21|Neobisium tantaleum|CROBD654|Neobisium tantaleum|Subgenus: Blothrus CROPS168-21|Neobisium

tantaleum|CROBD877|Neobisium tantaleum|Subgenus: Blothrus CROPS339-21|Neobisium tantaleum|CROBD660|Neobisium tantaleum|Subgenus: Blothrus CROPS179-21|Neobisium tantaleum|CROBD920|Neobisium tantaleum|Subgenus: Blothrus CROPS013-21|Neobisium tantaleum|CROBD399|Neobisium tantaleum|Subgenus: Blothrus CROPS216-21|Neobisium tantaleum|CROBD245|Neobisium tantaleum|Subgenus: Blothrus CROPS219-21|Neobisium tantaleum|CROBD248|Neobisium tantaleum|Subgenus: Blothrus

Group 27 n: 1; id: CROPS508-22|Olpium pallipes|CROBD634|Olpium pallipes|

Group 28 n: 3; id: CROPS458-22|Neobisium sylvaticum|CROBD1134|Neobisium sylvaticum|Subgenus: Neobisium CROPS208-21|Neobisium sylvaticum|CROBD234|Neobisium sylvaticum|Subgenus: Neobisium CROPS207-21|Neobisium sylvaticum|CROBD233|Neobisium sylvaticum|Subgenus: Neobisium

Group 29 n: 4; id: CROPS459-22|Neobisium erythrodactylum|CROBD1135|Neobisium erythrodactylum|Subgenus: Neobisium CROPS460-22|Neobisium erythrodactylum|CROBD1136|Neobisium erythrodactylum|Subgenus: Neobisium CROPS461-22|Neobisium erythrodactylum|CROBD1137|Neobisium erythrodactylum|Subgenus: Neobisium CROPS356-21|Neobisium erythrodactylum|CROBD749|Neobisium erythrodactylum|Subgenus: Neobisium

Group 30 n: 4; id: CROPS462-22|Neobisium aff. stygium|CROBD1138|Neobisium aff. stygium|Subgenus: Blothrus CROPS318-21|Neobisium aff. stygium|CROBD620|Neobisium aff. stygium|Subgenus: Blothrus CROPS335-21|Neobisium aff. stygium|CROBD656|Neobisium aff. stygium|Subgenus: Blothrus CROPS278-21|Neobisium aff. stygium|CROBD346|Neobisium aff. stygium|Subgenus: Blothrus

Group 31 n: 3; id: CROPS464-22|Roncus sp. 4DH|CROBD1142|Roncus sp. 4DH| CROPS467-22|Roncus sp. 4DH|CROBD1146|Roncus sp. 4DH| CROPS497-22|Roncus sp. 4DH|CROBD1183|Roncus sp. 4DH|

Group 32 n: 5; id: CROPS465-22|Roncus aff. meledae|CROBD1143|Roncus aff. meledae| CROPS256-21|Roncus aff. meledae|CROBD305|Roncus aff. meledae| CROPS257-21|Roncus aff. meledae|CROBD307|Roncus aff. meledae| CROPS189-21|Roncus aff. meledae|CROBD208|Roncus aff. meledae| CROPS259-21|Roncus aff. meledae|CROBD314|Roncus aff. meledae|

Group 33 n: 5; id: CROPS466-22|Neobisium oculatum|CROBD1144|Neobisium oculatum|Subgenus: Neobisium CROPS490-22|Neobisium aff. oculatum|CROBD1174|Neobisium aff. oculatum|Subgenus: Neobisium CROPS492-22|Neobisium aff. oculatum|CROBD1177|Neobisium aff. oculatum|Subgenus: Neobisium CROPS491-22|Neobisium aff. oculatum|CROBD1176|Neobisium aff. oculatum|Subgenus: Neobisium CROPS383-22|Neobisium oculatum|CROBD1011|Neobisium oculatum|Subgenus: Neobisium

Group 34 n: 2; id: CROPS468-22|Roncus aff. belluatii|CROBD1147|Roncus aff. belluatii| CROPS120-21|Roncus aff. belluatii|CROBD739|Roncus aff. belluatii|

Group 35 n: 6; id: CROPS469-22|Chthonius raridentatus|CROBD1149|Chthonius raridentatus| CROPS399-22|Chthonius raridentatus|CROBD1102|Chthonius raridentatus| CROPS401-22|Chthonius raridentatus|CROBD1104|Chthonius raridentatus| CROPS474-22|Chthonius sp. nov. K|CROBD1156|Chthonius sp. nov. K| CROPS262-21|Chthonius sp. nov. K|CROBD321|Chthonius sp. nov. K| CROPS307-21|Chthonius sp. nov. K|CROBD527|Chthonius sp. nov. K|

Group 36 n: 2; id: CROPS470-22|Chthonius sp. LI|CROBD1151|Chthonius sp. LI| CROPS402-22|Chthonius sp. ZB|CROBD1105|Chthonius sp. ZB|

Group 37 n: 12; id: CROPS471-22|Chthonius magnificus|CROBD1152|Chthonius magnificus| CROPS366-21|Chthonius magnificus|CROBD761|Chthonius magnificus| CROPS368-21|Chthonius magnificus|CROBD763|Chthonius magnificus| CROPS483-22|Chthonius magnificus|CROBD1165|Chthonius magnificus| CROPS410-22|Chthonius magnificus|CROBD1116|Chthonius magnificus| CROPS412-22|Chthonius magnificus|CROBD1120|Chthonius magnificus| CROPS154-21|Chthonius magnificus|CROBD838|Chthonius magnificus| CROPS033-21|Chthonius magnificus|CROBD432|Chthonius magnificus| CROPS496-22|Chthonius magnificus|CROBD1182|Chthonius magnificus| CROPS023-21|Chthonius magnificus|CROBD412|Chthonius magnificus| CROPS503-22|Chthonius magnificus|CROBD1190|Chthonius magnificus| CROPS416-22|Chthonius magnificus|CROBD1124|Chthonius magnificus|

Group 38 n: 2; id: CROPS472-22|Chthonius aff. ischnocheles|CROBD1154|Chthonius aff. ischnocheles| CROPS473-22|Chthonius aff. ischnocheles|CROBD1155|Chthonius aff. ischnocheles|

Group 39 n: 6; id: CROPS476-22|Chthonius subterraneus|CROBD1158|Chthonius subterraneus| CROPS109-21|Chthonius subterraneus meuseli|CROBD688|Chthonius subterraneus| CROPS115-21|Chthonius subterraneus meuseli|CROBD696|Chthonius subterraneus| CROPS124-21|Chthonius subterraneus meuseli|CROBD743|Chthonius subterraneus| CROPS122-21|Chthonius subterraneus meuseli|CROBD741|Chthonius subterraneus| CROPS121-21|Chthonius subterraneus meuseli|CROBD740|Chthonius subterraneus|

Group 40 n: 3; id: CROPS478-22|Roncus aff. belluatii 2|CROBD1160|Roncus aff. belluatii 2| CROPS119-21|Roncus aff. belluatii 2|CROBD738|Roncus aff. belluatii 2| CROPS352-21|Roncus aff. belluatii 2|CROBD674|Roncus aff. belluatii 2|

Group 41 n: 11; id: CROPS481-22|Neobisium sp. 1DH|CROBD1163|Neobisium sp. 1DH| CROPS489-22|Neobisium aff. svetovidi|CROBD1172|Neobisium aff. svetovidi|Subgenus: Blothrus CROPS083-21|Neobisium aff. svetovidi|CROBD603|Neobisium aff. svetovidi|Subgenus: Blothrus CROPS046-21|Neobisium aff. svetovidi|CROBD560|Neobisium aff. svetovidi|Subgenus: Blothrus CROPS057-21|Neobisium aff. svetovidi|CROBD571|Neobisium aff. svetovidi|Subgenus: Blothrus CROPS280-21|Neobisium aff. svetovidi|CROBD349|Neobisium aff. svetovidi|Subgenus: Blothrus CROPS059-21|Neobisium aff. svetovidi|CROBD574|Neobisium aff. svetovidi|Subgenus: Blothrus CROPS085-21|Neobisium aff. svetovidi|CROBD605|Neobisium aff. svetovidi|Subgenus: Blothrus CROPS132-21|Neobisium aff. svetovidi|CROBD798|Neobisium aff. svetovidi|Subgenus: Blothrus CROPS269-21|Neobisium aff. svetovidi|CROBD331|Neobisium aff. svetovidi|Subgenus: Blothrus CROPS134-21|Neobisium aff. svetovidi|CROBD801|Neobisium aff. svetovidi|Subgenus: Blothrus

Group 42 n: 4; id: CROPS482-22|Roncus sp. nov. P|CROBD1164|Roncus sp. nov. P| CROPS047-21|Roncus sp. nov. P|CROBD561|Roncus sp. nov. P| CROPS074-21|Roncus sp. nov. P|CROBD590|Roncus sp. nov. P| CROPS237-21|Roncus sp. nov. P|CROBD271|Roncus sp. nov. P|
Group 43 n: 4; id: CROPS484-22|Chthoniidae|CROBD1166||Globochthonius | Globochthonius sp. nov. D CROPS017-21|Chthoniidae|CROBD403||Globochthonius | Globochthonius sp. n. D CROPS350-21|Chthoniidae|CROBD672||Globochthonius | Globochthonius sp. n. D CROPS351-21|Chthoniidae|CROBD673||Globochthonius | Globochthonius sp. n. D
Group 44 n: 3; id: CROPS487-22|Neobisium dalmatinum|CROBD1170|Neobisium dalmatinum|Subgenus: Blothrus CROPS488-22|Neobisium dalmatinum|CROBD1171|Neobisium dalmatinum|Subgenus: Blothrus CROPS157-21|Neobisium dalmatinum|CROBD846|Neobisium dalmatinum|Subgenus: Blothrus
Group 45 n: 7; id: CROPS493-22|Chthonius occultus|CROBD1178|Chthonius occultus| CROPS165-21|Chthonius occultus|CROBD869|Chthonius occultus| CROPS035-21|Chthonius occultus|CROBD436|Chthonius occultus| CROPS028-21|Chthonius occultus|CROBD420|Chthonius occultus| CROPS024-21|Chthonius occultus|CROBD416|Chthonius occultus| CROPS502-22|Chthonius occultus|CROBD1189|Chthonius occultus| CROPS244-21|Chthonius occultus|CROBD282|Chthonius occultus|
Group 46 n: 1; id: CROPS495-22|Roncus aff. lubricus 1|CROBD1181|Roncus aff. lubricus 1|
Group 47 n: 1; id: CROPS499-22|Chthoniidae|CROBD1186||Ephippiochthonius | Ephippiochthonius insularis
Group 48 n: 12; id: CROPS506-22|Lasiochernes|CROBD1194||sp. 3DH CROPS386-22|Lasiochernes|CROBD1015||sp. 3DH CROPS070-21|Lasiochernes|CROBD586||sp. 3DH CROPS095-21|Lasiochernes|CROBD617||sp. 3DH CROPS236-21|Lasiochernes|CROBD269||sp. 3DH CROPS348-21|Lasiochernes|CROBD670||sp. 3DH CROPS349-21|Lasiochernes|CROBD671||sp. 3DH CROPS067-21|Lasiochernes|CROBD583||sp. 3DH CROPS072-21|Lasiochernes|CROBD588||sp. 3DH CROPS080-21|Lasiochernes|CROBD599||sp. 3DH CROPS086-21|Lasiochernes|CROBD607||sp. 3DH CROPS371-21|Lasiochernes|CROBD767||sp. 3DH
Group 49 n: 1; id: CROPS507-22|Roncus sp. nov. SO|CROBD1195|Roncus sp. nov. SO|
Group 50 n: 2; id: CROPS382-22|Roncus aff. lubricus|CROBD1005|Roncus aff. lubricus| CROPS243-21|Roncus aff. lubricus|CROBD277|Roncus aff. lubricus|
Group 51 n: 2; id: CROPS384-22|Chthonius sp. nov. M1|CROBD1012|Chthonius sp. nov. M1| CROPS385-22|Chthonius sp. nov. M1|CROBD1013|Chthonius sp. nov. M1|
Group 52 n: 2; id: CROPS387-22|Chthonius aff. exarmatus|CROBD1016|Chthonius aff. exarmatus| CROPS388-22|Chthonius aff. exarmatus|CROBD1017|Chthonius aff. exarmatus|
Group 53 n: 7; id: CROPS389-22|Neobisium dinaricum|CROBD1018|Neobisium dinaricum|Subgenus: Blothrus CROPS327-21|Neobisium dinaricum|CROBD639|Neobisium dinaricum|Subgenus: Blothrus CROPS261-21|Neobisium dinaricum|CROBD316|Neobisium dinaricum|Subgenus: Blothrus CROPS139-21|Neobisium dinaricum|CROBD816|Neobisium dinaricum|Subgenus: Blothrus CROPS169-21|Neobisium dinaricum|CROBD879|Neobisium dinaricum|Subgenus: Blothrus CROPS144-21|Neobisium dinaricum|CROBD825|Neobisium dinaricum|Subgenus: Blothrus CROPS173-21|Neobisium vjetrenicae|CROBD889|Neobisium vjetrenicae|Subgenus: Blothrus
Group 54 n: 4; id: CROPS390-22|Lasiochernes|CROBD1019||sp. 2DH CROPS365-21|Lasiochernes|CROBD760||sp. 2DH CROPS187-21|Lasiochernes|CROBD205||sp. 1DH CROPS248-21|Lasiochernes|CROBD290||sp. 1DH
Group 55 n: 1; id: CROPS396-22|Roncus italicus|CROBD1099|Roncus italicus|
Group 56 n: 3; id: CROPS397-22|Roncus italicus|CROBD1100|Roncus italicus| CROPS400-22|Roncus italicus|CROBD1103|Roncus italicus| CROPS403-22|Roncus italicus|CROBD1106|Roncus italicus|
Group 57 n: 1; id: CROPS406-22|Chthonius ischnocheles reductus|CROBD1109|Chthonius ischnocheles|
Group 58 n: 6; id: CROPS407-22|Chthonius ischnocheles ischnocheles|CROBD1110|Chthonius ischnocheles| CROPS409-22|Chthonius ischnocheles ischnocheles|CROBD1114|Chthonius ischnocheles| CROPS285-21|Chthonius ischnocheles|CROBD358|Chthonius ischnocheles| CROPS323-21|Chthonius ischnocheles|CROBD635|Chthonius ischnocheles| CROPS325-21|Chthonius ischnocheles|CROBD637|Chthonius ischnocheles| CROPS326-21|Chthonius ischnocheles|CROBD638|Chthonius ischnocheles|
Group 59 n: 1; id: CROPS408-22|Chthonius sp. ZB|CROBD1113|Chthonius sp. ZB|
Group 60 n: 3; id: CROPS411-22|Roncus aff. ragusae|CROBD1117|Roncus aff. ragusae| CROPS415-22|Roncus aff. ragusae|CROBD1123|Roncus aff. ragusae| CROPS021-21|Roncus ragusae|CROBD410|Roncus ragusae|
Group 61 n: 2; id: CROPS413-22|Roncus insularis|CROBD1121|Roncus insularis| CROPS414-22|Roncus insularis|CROBD1122|Roncus insularis|
Group 62 n: 3; id: CROPS001-21|Roncus lubricus|CROBD382|Roncus lubricus|Archaeoroncus dalmatinus CROPS184-21|Roncus lubricus|CROBD202|Roncus lubricus|Archaeoroncus dalmatinus CROPS206-21|Roncus lubricus|CROBD232|Roncus lubricus|Archaeoroncus dalmatinus
Group 63 n: 2; id: CROPS006-21|Neobisium peruni|CROBD388|Neobisium peruni|Subgenus: Blothrus CROPS019-21|Neobisium peruni|CROBD405|Neobisium peruni|Subgenus: Blothrus
Group 64 n: 1; id: CROPS016-21|Troglochthonius aff. mirabilis|CROBD402|Troglochthonius aff. mirabilis|
Group 65 n: 1; id: CROPS022-21|Troglochthonius mirabilis|CROBD411|Troglochthonius mirabilis|
Group 66 n: 3; id: CROPS025-21|Chthonius subterraneus subterraneus|CROBD417|Chthonius subterraneus| CROPS026-21|Chthonius subterraneus subterraneus|CROBD418|Chthonius subterraneus| CROPS161-21|Chthonius subterraneus subterraneus|CROBD862|Chthonius subterraneus|
Group 67 n: 1; id: CROPS027-21|Neobisium heros|CROBD419|Neobisium heros|Subgenus: Blothrus
Group 68 n: 1; id: CROPS029-21|Chthoniidae|CROBD423||Ephippiochthonius | Ephippiochthonius insularis
Group 69 n: 1; id: CROPS030-21|Insulocreagris sp. n. M|CROBD424|Insulocreagris sp. n. M|

Group 70 n: 2; id: CROPS031-21|Roncus sp. n. P|CROBD426|Roncus sp. n. P| CROPS032-21|Roncus sp. n. P|CROBD427|Roncus sp. n. P|
Group 71 n: 1; id: CROPS036-21|Chthoniidae|CROBD437||Globochthonius | Globochthonius caligatus
Group 72 n: 1; id: CROPS037-21|Neobisium aff. heros|CROBD439|Neobisium aff. heros|
Group 73 n: 1; id: CROPS038-21|Occidenchthonius sp. PEL|CROBD442|Occidenchthonius sp. PEL|
Group 74 n: 3; id: CROPS039-21|Roncus anophthalmus|CROBD443|Roncus anophthalmus| CROPS264-21|Roncus anophthalmus|CROBD325|Roncus anophthalmus| CROPS273-21|Roncus anophthalmus|CROBD337|Roncus anophthalmus|
Group 75 n: 1; id: CROPS041-21|Roncus sp. 3DH|CROBD554|Roncus sp. 3DH|
Group 76 n: 1; id: CROPS042-21|Chthoniidae|CROBD555||Ephippiochthonius | Ephippiochthonius sp. 1DH
Group 77 n: 1; id: CROPS043-21|Chthoniidae|CROBD557||Ephippiochthonius | Ephippiochthonius insularis
Group 78 n: 1; id: CROPS049-21|Chthonius sp. nov. V|CROBD563|Chthonius sp. nov. V|
Group 79 n: 2; id: CROPS050-21|Chthonius aff. radjai|CROBD564|Chthonius aff. radjai| CROPS162-21|Chthonius aff. radjai|CROBD863|Chthonius aff. radjai|
Group 80 n: 5; id: CROPS051-21|Neobisium aff. svetovidi|CROBD565|Neobisium aff. svetovidi|Subgenus: Blothrus CROPS271-21|Neobisium aff. svetovidi|CROBD334|Neobisium aff. svetovidi|Subgenus: Blothrus CROPS078-21|Neobisium aff. svetovidi|CROBD597|Neobisium aff. svetovidi|Subgenus: Blothrus CROPS089-21|Neobisium aff. svetovidi|CROBD610|Neobisium aff. svetovidi|Subgenus: Blothrus CROPS321-21|Neobisium aff. svetovidi|CROBD623|Neobisium aff. svetovidi|Subgenus: Blothrus
Group 81 n: 4; id: CROPS054-21|Chthonius aff. absoloni|CROBD568|Chthonius aff. absoloni| CROPS056-21|Chthonius aff. absoloni|CROBD570|Chthonius aff. absoloni| CROPS071-21|Chthonius aff. absoloni|CROBD587|Chthonius aff. absoloni| CROPS084-21|Chthonius sp. nov. P|CROBD604|Chthonius sp. nov. P|
Group 82 n: 3; id: CROPS058-21|Insulocreagris sp. nov. P1|CROBD573|Insulocreagris sp. nov. P1| CROPS081-21|Insulocreagris sp. nov. P1|CROBD600|Insulocreagris sp. nov. P1| CROPS091-21|Insulocreagris sp. nov. P1|CROBD612|Insulocreagris sp. nov. P1|
Group 83 n: 1; id: CROPS060-21|Insulocreagris sp. 3DH|CROBD575|Insulocreagris sp. 3DH|
Group 84 n: 1; id: CROPS061-21|Neobisium elegans|CROBD576|Neobisium elegans|Subgenus: Neobisium
Group 85 n: 12; id: CROPS062-21|Chthonius sp. nov. PLJ|CROBD577|Chthonius sp. nov. PLJ| CROPS379-21|Chthonius sp. nov. PLJ|CROBD778|Chthonius sp. nov. PLJ| CROPS090-21|Chthonius sp. nov. PLJ|CROBD611|Chthonius sp. nov. PLJ| CROPS369-21|Chthonius sp. nov. PLJ|CROBD764|Chthonius sp. nov. PLJ| CROPS228-21|Chthonius aff. absoloni|CROBD258|Chthonius aff. absoloni| CROPS096-21|Chthonius aff. absoloni|CROBD618|Chthonius aff. absoloni| CROPS232-21|Chthonius aff. absoloni|CROBD262|Chthonius aff. absoloni| CROPS233-21|Chthonius aff. absoloni|CROBD263|Chthonius aff. absoloni| CROPS202-21|Chthonius sp. 1DH|CROBD225|Chthonius sp. 1DH| CROPS212-21|Chthonius aff. alpicola|CROBD241|Chthonius aff. alpicola| CROPS214-21|Chthonius aff. alpicola|CROBD243|Chthonius aff. alpicola| CROPS245-21|Chthonius aff. alpicola|CROBD283|Chthonius aff. alpicola|
Group 86 n: 3; id: CROPS073-21|Roncus lubricus|CROBD589|Roncus lubricus| CROPS377-21|Roncus lubricus|CROBD776|Roncus lubricus| CROPS378-21|Roncus lubricus|CROBD777|Roncus lubricus|
Group 87 n: 3; id: CROPS075-21|Neobisium aff. dalmatinum|CROBD591|Neobisium aff. dalmatinum|Subgenus: Blothrus CROPS082-21|Neobisium aff. dalmatinum|CROBD601|Neobisium aff. dalmatinum|Subgenus: Blothrus CROPS079-21|Neobisium aff. dalmatinum|CROBD598|Neobisium aff. dalmatinum|Subgenus: Blothrus
Group 88 n: 1; id: CROPS076-21|Insulocreagris sp. nov. P2|CROBD592|Insulocreagris sp. nov. P2|
Group 89 n: 1; id: CROPS094-21|Roncus narentae|CROBD616|Roncus narentae|
Group 90 n: 1; id: CROPS102-21|Chthonius sp. LK|CROBD680|Chthonius sp. LK|
Group 91 n: 1; id: CROPS137-21|Chthoniidae|CROBD813||Globochthonius | Globochthonius sp. nov. DO
Group 92 n: 2; id: CROPS138-21|Roncus aff. lubricus 3|CROBD815|Roncus aff. lubricus 3| CROPS247-21|Roncus aff. lubricus 3|CROBD289|Roncus aff. lubricus 3|
Group 93 n: 8; id: CROPS147-21|Neobisium aff. reimoseri|CROBD828|Neobisium aff. reimoseri|Subgenus: Blothrus CROPS195-21|Neobisium reimoseri|CROBD217|Neobisium reimoseri|Subgenus: Blothrus CROPS337-21|Neobisium reimoseri|CROBD658|Neobisium reimoseri|Subgenus: Blothrus CROPS338-21|Neobisium reimoseri|CROBD659|Neobisium reimoseri|Subgenus: Blothrus CROPS343-21|Neobisium reimoseri|CROBD664|Neobisium reimoseri|Subgenus: Blothrus CROPS346-21|Neobisium reimoseri|CROBD667|Neobisium reimoseri|Subgenus: Blothrus CROPS250-21|Neobisium reimoseri|CROBD294|Neobisium reimoseri|Subgenus: Blothrus CROPS360-21|Neobisium reimoseri|CROBD754|Neobisium reimoseri|Subgenus: Blothrus
Group 94 n: 1; id: CROPS151-21|Chthoniidae|CROBD834||Globochthonius | Globochthonius aff. caligatus
Group 95 n: 1; id: CROPS152-21|Neobisium sp. nov. KP|CROBD835|Neobisium sp. nov. KP|
Group 96 n: 1; id: CROPS153-21|Insulocreagris sp. n. M|CROBD837|Insulocreagris sp. n. M|
Group 97 n: 1; id: CROPS160-21|Roncus lubricus|CROBD851|Roncus lubricus|
Group 98 n: 2; id: CROPS164-21|Chthonius sp. nov. D|CROBD868|Chthonius sp. nov. D| CROPS347-21|Chthonius sp. nov. KO|CROBD669|Chthonius sp. nov. KO|
Group 99 n: 1; id: CROPS174-21|Roncus aff. insularis|CROBD904|Roncus aff. insularis|
Group 100 n: 1; id: CROPS175-21|Roncus lubricus|CROBD906|Roncus lubricus|Archaeoroncus dalmatinus
Group 101 n: 1; id: CROPS176-21|Chthoniidae|CROBD907||Microchthonius | Microchthonius elegantissimus
Group 102 n: 3; id: CROPS180-21|Chthonius sp. nov. VL|CROBD921|Chthonius sp. nov. VL| CROPS181-21|Chthonius sp. nov. VL|CROBD922|Chthonius sp. nov. VL| CROPS290-21|Chthonius sp. nov. VL|CROBD368|Chthonius sp. nov. VL|
Group 103 n: 3; id: CROPS183-21|Neobisium stribogi|CROBD201|Neobisium stribogi|Subgenus: Pennobisium CROPS192-21|Neobisium stribogi|CROBD213|Neobisium stribogi|Subgenus: Pennobisium CROPS193-21|Neobisium stribogi|CROBD215|Neobisium stribogi|Subgenus: Pennobisium

Group 104 n: 2; id: CROPS186-21|Insulocreagris sp. 1DH|CROBD204|Insulocreagris sp. 1DH| CROPS302-21|Insulocreagris sp. 1DH|CROBD521|Insulocreagris sp. 1DH|
Group 105 n: 3; id: CROPS191-21|Neobisium sp. nov. M|CROBD212|Neobisium sp. nov. M| CROPS353-21|Neobisium sp. nov. M|CROBD700|Neobisium sp. nov. M| CROPS319-21|Neobisium sp. nov. M|CROBD621|Neobisium sp. nov. M|
Group 106 n: 2; id: CROPS194-21|Neobisium carcinoides|CROBD216|Neobisium carcinoides|Subgenus: Neobisium CROPS296-21|Neobisium carcinoides|CROBD378|Neobisium carcinoides|Subgenus: Neobisium
Group 107 n: 1; id: CROPS196-21|Roncus sp. 2DH|CROBD218|Roncus sp. 2DH|
Group 108 n: 3; id: CROPS203-21|Roncus sp. nov. G|CROBD229|Roncus sp. nov. G| CROPS204-21|Roncus sp. nov. G|CROBD230|Roncus sp. nov. G| CROPS205-21|Roncus sp. nov. G|CROBD231|Roncus sp. nov. G|
Group 109 n: 2; id: CROPS211-21|Roncus podaga|CROBD237|Roncus podaga| CROPS213-21|Roncus podaga|CROBD242|Roncus podaga|
Group 110 n: 1; id: CROPS215-21|Chthonius sp. 2DH|CROBD244|Chthonius sp. 2DH|
Group 111 n: 2; id: CROPS226-21|Neobisium aff. elegans|CROBD256|Neobisium aff. elegans|Subgenus: Neobisium CROPS330-21|Neobisium aff. elegans|CROBD645|Neobisium aff. elegans|Subgenus: Neobisium
Group 112 n: 1; id: CROPS231-21|Roncus aff. lubricus|CROBD261|Roncus aff. lubricus|
Group 113 n: 3; id: CROPS239-21|Insulocreagris sp. TS|CROBD273|Insulocreagris sp. TS| CROPS240-21|Insulocreagris sp. TS|CROBD274|Insulocreagris sp. TS| CROPS241-21|Insulocreagris sp. TS|CROBD275|Insulocreagris sp. TS|
Group 114 n: 2; id: CROPS251-21|Chthoniidae|CROBD298||Ephippiochthonius | Ephippiochthonius insularis CROPS252-21|Chthoniidae|CROBD300||Ephippiochthonius | Ephippiochthonius insularis
Group 115 n: 2; id: CROPS258-21|Chthonius sp. nov. M2|CROBD310|Chthonius sp. nov. M2| CROPS260-21|Chthonius sp. nov. M2|CROBD315|Chthonius sp. nov. M2|
Group 116 n: 1; id: CROPS284-21|Chthoniidae|CROBD355||Ephippiochthonius | Ephippiochthonius tetrachelatus
Group 117 n: 2; id: CROPS292-21|Neobisium sp. nov. M|CROBD371|Neobisium sp. nov. M| CROPS293-21|Neobisium sp. nov. M|CROBD372|Neobisium sp. nov. M|
Group 118 n: 3; id: CROPS294-21|Roncus sp. 1DH|CROBD376|Roncus sp. 1DH| CROPS297-21|Roncus sp. 1DH|CROBD379|Roncus sp. 1DH| CROPS298-21|Roncus sp. 1DH|CROBD380|Roncus sp. 1DH|
Group 119 n: 1; id: CROPS295-21|Roncus aff. italicus|CROBD377|Roncus aff. italicus|
Group 120 n: 1; id: CROPS301-21|Garypus sp. nov. M|CROBD519|Garypus sp. nov. M|
Group 121 n: 2; id: CROPS305-21|Roncus sp. nov. S|CROBD525|Roncus sp. nov. S| CROPS306-21|Roncus sp. nov. S|CROBD526|Roncus sp. nov. S|
Group 122 n: 1; id: CROPS324-21|Insulocreagris sp. 3DH|CROBD636|Insulocreagris sp. 3DH|
Group 123 n: 2; id: CROPS328-21|Rhacochelifer maculatus|CROBD641|Rhacochelifer maculatus| CROPS331-21|Rhacochelifer maculatus|CROBD646|Rhacochelifer maculatus|
Group 124 n: 1; id: CROPS329-21|Chthonius aff. heterodactylus|CROBD643|Chthonius aff. heterodactylus|
Group 125 n: 1; id: CROPS342-21|Neobisium fuscimanum|CROBD663|Neobisium fuscimanum|Subgenus: Neobisium
Group 126 n: 1; id: CROPS357-21|Roncus aff. lubricus|CROBD750|Roncus aff. lubricus|
Group 127 n: 1; id: CROPS367-21|Roncus aff. ragusae|CROBD762|Roncus aff. ragusae|
Group 128 n: 1; id: CROPS370-21|Chthoniidae|CROBD766||Occidenchthonius | Occidenchthonius sp. nov. PR
Group 129 n: 1; id: CROPS372-21|Chthoniidae|CROBD768||Ephippiochthonius | Ephippiochthonius aff. insularis
Group 130 n: 1; id: CROPS374-21|Roncus aff. lubricus|CROBD771|Roncus aff. lubricus|

bPTP

Group 1 (support = 1.000)
'CROPS508-22|Olpium_pallipes|CROBD634|Olpium_pallip'
Group 2 (support = 1.000)
'CROPS301-21|Garypus_sp._nov. M|CROBD519|Garypus_sp'
Group 3 (support = 0.499)
'CROPS302-21|Insulocreagris_sp._1DH|CROBD521|Insulo','CROPS186-21|Insulocreagris_sp._1DH|CROBD204|Insulo'
Group 4 (support = 1.000)
'CROPS432-22|Insulocreagris_sp._2DH|CROBD830|Insulo'
Group 5 (support = 1.000)
'CROPS076-21|Insulocreagris_sp._nov.P2|CROBD592|In'
Group 6 (support = 1.000)
'CROPS370-21|Chthoniidae|CROBD766||Occidenchthonius'
Group 7 (support = 1.000)
'CROPS038-21|Occidenchthonius_sp._PEL|CROBD442|Occi'
Group 8 (support = 1.000)
'CROPS049-21|Chthonius_sp._nov.V|CROBD563|Chthoniu'
Group 9 (support = 0.517)

'CROPS316-21|Chernes_hahnii|CROBD594|Chernes_hahnii','CROPS30021|Chernes_hahnii|CROBD468|Chernes_hahnii','CROPS421-22|Chernes_hahnii|CROBD632|Chernes_hahnii','CROPS317-21|Chernes_hahnii|CROBD595|Chernes_hahnii'

Group 10 (support = 0.987)

'CROPS328-21|Rhacochelifer_maculatus|CROBD641|Rhaco','CROPS331-21|Rhacochelifer_maculatus|CROBD646|Rhaco'

Group 11 (support = 1.000)

'CROPS174-21|Roncus_aff._insularis|CROBD904|Roncus'

Group 12 (support = 0.909)

'CROPS211-21|Roncus_podaga|CROBD237|Roncus_podaga|','CROPS213-21|Roncus_podaga|CROBD242|Roncus_podaga|'

Group 13 (support = 0.987)

'CROPS414-22|Roncus_insularis|CROBD1122|Roncus_insu','CROPS413-22|Roncus_insularis|CROBD1121|Roncus_insu'

Group 14 (support = 1.000)

'CROPS396-22|Roncus_italicus|CROBD1099|Roncus_itali'

Group 15 (support = 1.000)

'CROPS495-22|Roncus_aff._lubricus_1|CROBD1181|Roncu'

Group 16 (support = 0.738)

'CROPS208-21|Neobisium_sylvaticum|CROBD234|Neobisiu','CROPS207-21|Neobisium_sylvaticum|CROBD233|Neobisiu','CROPS458-22|Neobisium_sylvaticum|CROBD1134|Neobisi'

Group 17 (support = 0.879)

'CROPS031-21|Roncus_sp._n._P|CROBD426|Roncus_sp._n.','CROPS032-21|Roncus_sp._n._P|CROBD427|Roncus_sp._n.'

Group 18 (support = 1.000)

'CROPS342-21|Neobisium_fuscimanum|CROBD663|Neobisiu'

Group 19 (support = 0.235)

'CROPS461-22|Neobisium_erythrodactylum|CROBD1137|Ne','CROPS459-22|Neobisium_erythrodactylum|CROBD1135|Ne','CROPS356-21|Neobisium_erythrodactylum|CROBD749|Neo','CROPS460-22|Neobisium_erythrodactylum|CROBD1136|Ne'

Group 20 (support = 0.717)

'CROPS488-22|Neobisium_dalmatinum|CROBD1171|Neobisi','CROPS487-22|Neobisium_dalmatinum|CROBD1170|Neobisi','CROPS157-21|Neobisium_dalmatinum|CROBD846|Neobisiu'

Group 21 (support = 0.270)

'CROPS296-21|Neobisium_carcinoides|CROBD378|Neobisi','CROPS194-21|Neobisium_carcinoides|CROBD216|Neobisi'

Group 22 (support = 1.000)

'CROPS215-21|Chthonius_sp._2DH|CROBD244|Chthonius_s'

Group 23 (support = 1.000)

'CROPS016-21|Troglochthonius_aff._mirabilis|CROBD40'

Group 24 (support = 1.000)

'CROPS022-21|Troglochthonius_mirabilis|CROBD411|Tro'

Group 25 (support = 1.000)

'CROPS041-21|Roncus_sp._3DH|CROBD554|Roncus_sp._3DH'

Group 26 (support = 1.000)

'CROPS094-21|Roncus_narentae|CROBD616|Roncus_narent'

Group 27 (support = 0.892)

'CROPS402-22|Chthonius_sp._ZB|CROBD1105|Chthonius_s','CROPS470-22|Chthonius_sp._LI|CROBD1151|Chthonius_s'

Group 28 (support = 1.000)

'CROPS408-22|Chthonius_sp._ZB|CROBD1113|Chthonius_s'

Group 29 (support = 1.000)

'CROPS042-21|Chthoniidae|CROBD555||Ephippiochthoniu'

Group 30 (support = 1.000)

'CROPS176-21|Chthoniidae|CROBD907||Microchthonius_|'

Group 31 (support = 1.000)

'CROPS102-21|Chthonius_sp._LK|CROBD680|Chthonius_sp'

Group 32 (support = 1.000)

'CROPS160-21|Roncus_lubricus|CROBD851|Roncus_lubric'

Group 33 (support = 0.511)

'CROPS247-21|Roncus_aff._lubricus_3|CROBD289|Roncus','CROPS138-21|Roncus_aff._lubricus_3|CROBD815|Roncus'

Group 34 (support = 1.000)

'CROPS507-22|Roncus_sp._nov._SO|CROBD1195|Roncus_sp'

Group 35 (support = 0.903)

'CROPS073-21|Roncus_lubricus|CROBD589|Roncus_lubric','CROPS377-21|Roncus_lubricus|CROBD776|Roncus_lubric','CROPS378-21|Roncus_lubricus|CROBD777|Roncus_lubric'

Group 36 (support = 1.000)
'CROPS438-22|Chthoniidae|CROBD875||Globochthonius_|'
Group 37 (support = 1.000)
'CROPS329-21|Chthonius_aff._heterodactylus|CROBD643'
Group 38 (support = 1.000)
'CROPS428-22|Chthonius_aff._subterraneus|CROBD800|C'
Group 39 (support = 1.000)
'CROPS406-22|Chthonius_ischnocheles_reductus|CROBD1'
Group 40 (support = 0.517)
'CROPS473-22|Chthonius_aff._ischnocheles|CROBD1155|','CROPS472-22|Chthonius_aff._ischnocheles|CROBD1154|'
Group 41 (support = 0.333)
'CROPS241-21|Insulocreagris_sp._TS|CROBD275|Insuloc','CROPS239-21|Insulocreagris_sp._TS|CROBD273|Insuloc','CROPS240-21|Insulocreagris_sp._TS|CROBD274|Insuloc'
Group 42 (support = 0.762)
'CROPS101-21|Chthonius_sp._nov._L|CROBD679|Chthoniu','CROPS435-22|Chthonius_sp._nov._L|CROBD865|Chthoniu'
Group 43 (support = 0.872)
'CROPS387-22|Chthonius_aff._exarmatus|CROBD1016|Cht','CROPS388-22|Chthonius_aff._exarmatus|CROBD1017|Cht'
Group 44 (support = 0.724)
'CROPS238-21|Chthonius_exarmatus|CROBD272|Chthonius','CROPS044-21|Chthonius_exarmatus|CROBD558|Chthonius','CROPS045-21|Chthonius_exarmatus|CROBD559|Chthonius','CROPS426-22|Chthonius_exarmatus|CROBD765|Chthonius'
Group 45 (support = 0.892)
'CROPS287-21|Chthoniidae|CROBD362||Ephippiochthoniu','CROPS286-21|Chthoniidae|CROBD359||Ephippiochthoniu','CROPS289-21|Chthoniidae|CROBD365||Ephippiochthoniu'
Group 46 (support = 1.000)
'CROPS284-21|Chthoniidae|CROBD355||Ephippiochthoniu'
Group 47 (support = 1.000)
'CROPS288-21|Chthoniidae|CROBD364||Ephippiochthoniu'
Group 48 (support = 1.000)
'CROPS499-22|Chthoniidae|CROBD1186||Ephippiochthoni'
Group 49 (support = 1.000)
'CROPS137-21|Chthoniidae|CROBD813||Globochthonius_|'
Group 50 (support = 1.000)
'CROPS196-21|Roncus_sp._2DH|CROBD218|Roncus_sp._2DH'
Group 51 (support = 1.000)
'CROPS451-22|Roncus_aff._lubricus_2|CROBD910|Roncus'
Group 52 (support = 0.891)
'CROPS305-21|Roncus_sp._nov._S|CROBD525|Roncus_sp.','CROPS306-21|Roncus_sp._nov._S|CROBD526|Roncus_sp.'
Group 53 (support = 1.000)
'CROPS175-21|Roncus_lubricus|CROBD906|Roncus_lubric'
Group 54 (support = 1.000)
'CROPS357-21|Roncus_aff._lubricus|CROBD750|Roncus_a'
Group 55 (support = 1.000)
'CROPS295-21|Roncus_aff._italicus|CROBD377|Roncus_a'
Group 56 (support = 1.000)
'CROPS151-21|Chthoniidae|CROBD834||Globochthonius_|'
Group 57 (support = 1.000)
'CROPS037-21|Neobisium_aff._heros|CROBD439|Neobisiu'
Group 58 (support = 0.534)
'CROPS079-21|Neobisium_aff._dalmatinum|CROBD598|Neo','CROPS082-21|Neobisium_aff._dalmatinum|CROBD601|Neo','CROPS075-21|Neobisium_aff._dalmatinum|CROBD591|Neo'
Group 59 (support = 1.000)
'CROPS429-22|Chthonius_sp._nov._R|CROBD806|Chthoniu'
Group 60 (support = 0.486)
'CROPS096-21|Chthonius_aff._absoloni|CROBD618|Chtho','CROPS233-21|Chthonius_aff._absoloni|CROBD263|Chtho','CROPS232-21|Chthonius_aff._absoloni|CROBD262|Chtho'
Group 61 (support = 1.000)
'CROPS419-22|Neobisium_aff._vachoni|CROBD413|Neobis'
Group 62 (support = 0.790)
'CROPS482-22|Roncus_sp._nov._P|CROBD1164|Roncus_sp.','CROPS074-21|Roncus_sp._nov._P|CROBD590|Roncus_sp.','CROPS237-21|Roncus_sp._nov._P|CROBD271|Roncus_sp.','CROPS047-21|Roncus_sp._nov._P|CROBD561|Roncus_sp.'
Group 63 (support = 0.808)

'CROPS205-21|Roncus_sp._nov._G|CROBD231|Roncus_sp.','CROPS204-21|Roncus_sp._nov._G|CROBD230|Roncus_sp.','CROPS203-21|Roncus_sp._nov._G|CROBD229|Roncus_sp.'
Group 64 (support = 1.000)
'CROPS374-21|Roncus_aff._lubricus|CROBD771|Roncus_a'
Group 65 (support = 1.000)
'CROPS231-21|Roncus_aff._lubricus|CROBD261|Roncus_a'

Group 66 (support = 0.873)
'CROPS181-21|Chthonius_sp._nov._VL|CROBD922|Chthoni','CROPS180-21|Chthonius_sp._nov._VL|CROBD921|Chthoni','CROPS290-21|Chthonius_sp._nov._VL|CROBD368|Chthoni'
Group 67 (support = 0.247)
'CROPS162-21|Chthonius_aff._radjai|CROBD863|Chthoni','CROPS050-21|Chthonius_aff._radjai|CROBD564|Chthoni'
Group 68 (support = 1.000)
'CROPS061-21|Neobisium_elegans|CROBD576|Neobisium_e'
Group 69 (support = 0.289)
'CROPS502-22|Chthonius_occultus|CROBD1189|Chthonius','CROPS244-21|Chthonius_occultus|CROBD282|Chthonius','CROPS024-21|Chthonius_occultus|CROBD416|Chthonius','CROPS035-21|Chthonius_occultus|CROBD436|Chthonius','CROPS493-22|Chthonius_occultus|CROBD1178|Chthonius','CROPS165-21|Chthonius_occultus|CROBD869|Chthonius','CROPS028-21|Chthonius_occultus|CROBD420|Chthonius'
Group 70 (support = 0.821)
'CROPS293-21|Neobisium_sp._nov._M|CROBD372|Neobisiu','CROPS292-21|Neobisium_sp._nov._M|CROBD371|Neobisiu'
Group 71 (support = 0.813)
'CROPS439-22|Chthonius_trebinjensis|CROBD876|Chthon','CROPS265-21|Chthonius_trebinjensis|CROBD327|Chthon','CROPS040-21|Chthonius_trebinjensis|CROBD444|Chthon','CROPS034-21|Chthonius_trebinjensis|CROBD435|Chthon','CROPS332-21|Chthonius_trebinjensis|CROBD653|Chthon','CROPS494-22|Chthonius_trebinjensis|CROBD1180|Chtho'
Group 72 (support = 0.926)
'CROPS091-21|Insulocreagris_sp._nov._P1|CROBD612|In','CROPS058-21|Insulocreagris_sp._nov._P1|CROBD573|In','CROPS081-21|Insulocreagris_sp._nov._P1|CROBD600|In'
Group 73 (support = 0.455)
'CROPS189-21|Roncus_aff._meledae|CROBD208|Roncus_af','CROPS259-21|Roncus_aff._meledae|CROBD314|Roncus_af','CROPS257-21|Roncus_aff._meledae|CROBD307|Roncus_af','CROPS465-22|Roncus_aff._meledae|CROBD1143|Roncus_a','CROPS256-21|Roncus_aff._meledae|CROBD305|Roncus_af'
Group 74 (support = 0.933)
'CROPS497-22|Roncus_sp._4DH|CROBD1183|Roncus_sp._4D','CROPS464-22|Roncus_sp._4DH|CROBD1142|Roncus_sp._4D','CROPS467-22|Roncus_sp._4DH|CROBD1146|Roncus_sp._4D'
Group 75 (support = 1.000)
'CROPS030-21|Insulocreagris_sp._n._M|CROBD424|Insul'
Group 76 (support = 1.000)
'CROPS153-21|Insulocreagris_sp._n._M|CROBD837|Insul'
Group 77 (support = 1.000)
'CROPS029-21|Chthoniidae|CROBD423||Ephippiochthoniu'
Group 78 (support = 1.000)
'CROPS043-21|Chthoniidae|CROBD557||Ephippiochthoniu'
Group 79 (support = 1.000)
'CROPS036-21|Chthoniidae|CROBD437||Globochthonius_|'
Group 80 (support = 0.564)
'CROPS468-22|Roncus_aff._belluatii|CROBD1147|Roncus','CROPS120-21|Roncus_aff._belluatii|CROBD739|Roncus'
Group 81 (support = 1.000)
'CROPS027-21|Neobisium_heros|CROBD419|Neobisium_her'
Group 82 (support = 1.000)
'CROPS441-22|Neobisium_heros|CROBD880|Neobisium_her'
Group 83 (support = 1.000)
'CROPS173-21|Neobisium_vjetrenicae|CROBD889|Neobisi'
Group 84 (support = 0.371)
'CROPS327-21|Neobisium_dinaricum|CROBD639|Neobisium','CROPS389-22|Neobisium_dinaricum|CROBD1018|Neobisiu','CROPS261-21|Neobisium_dinaricum|CROBD316|Neobisium','CROPS169-21|Neobisium_dinaricum|CROBD879|Neobisium','CROPS139-21|Neobisium_dinaricum|CROBD816|Neobisium'
Group 85 (support = 1.000)
'CROPS144-21|Neobisium_dinaricum|CROBD825|Neobisium'
Group 86 (support = 1.000)
'CROPS152-21|Neobisium_sp._nov._KP|CROBD835|Neobisi'
Group 87 (support = 0.885)

'CROPS352-21|Roncus_belluatii|CROBD674|Roncus_bellu','CROPS119-21|Roncus_belluatii|CROBD738|Roncus_bellu','CROPS478-22|Roncus_belluatii|CROBD1160|Roncus_bell'

Group 88 (support = 1.000)

'CROPS060-21|Insulocreagris_sp._3DH|CROBD575|Insulo'

Group 89 (support = 1.000)

'CROPS324-21|Insulocreagris_sp._3DH|CROBD636|Insulo'

Group 90 (support = 0.483)

'CROPS067-21|Lasiochernes|CROBD583||sp._3DH','CROPS236-21|Lasiochernes|CROBD269||sp._3DH','CROPS371-21|Lasiochernes|CROBD767||sp._3DH','CROPS072-21|Lasiochernes|CROBD588||sp._3DH','CROPS080-21|Lasiochernes|CROBD599||sp._3DH','CROPS086-21|Lasiochernes|CROBD607||sp._3DH','CROPS506-22|Lasiochernes|CROBD1194||sp._3DH','CROPS349-21|Lasiochernes|CROBD671||sp._3DH','CROPS348-21|Lasiochernes|CROBD670||sp._3DH','CROPS386-22|Lasiochernes|CROBD1015||sp._3DH','CROPS095-21|Lasiochernes|CROBD617||sp._3DH','CROPS070-21|Lasiochernes|CROBD586||sp._3DH'

Group 91 (support = 0.852)

'CROPS384-22|Chthonius_sp._nov._M1|CROBD1012|Chthon','CROPS385-22|Chthonius_sp._nov._M1|CROBD1013|Chthon'

Group 92 (support = 0.887)

'CROPS251-21|Chthoniidae|CROBD298||Ephippiochthoniu','CROPS252-21|Chthoniidae|CROBD300||Ephippiochthoniu'

Group 93 (support = 1.000)

'CROPS372-21|Chthoniidae|CROBD768||Ephippiochthoniu'

Group 94 (support = 0.820)

'CROPS417-22|Neobisium_insulare|CROBD286|Neobisium','CROPS255-21|Neobisium_insulare|CROBD303|Neobisium','CROPS227-21|Neobisium_insulare|CROBD257|Neobisium','CROPS230-21|Neobisium_insulare|CROBD260|Neobisium','CROPS210-21|Neobisium_insulare|CROBD236|Neobisium','CROPS209-21|Neobisium_insulare|CROBD235|Neobisium','CROPS020-21|Neobisium_insulare|CROBD408|Neobisium','CROPS003-21|Neobisium_insulare|CROBD385|Neobisium','CROPS185-21|Neobisium_insulare|CROBD203|Neobisium'

Group 95 (support = 0.871)

'CROPS168-21|Neobisium_tantaleum|CROBD877|Neobisium','CROPS219-21|Neobisium_tantaleum|CROBD248|Neobisium','CROPS216-21|Neobisium_tantaleum|CROBD245|Neobisium','CROPS222-21|Neobisium_tantaleum|CROBD251|Neobisium','CROPS217-21|Neobisium_tantaleum|CROBD246|Neobisium','CROPS339-21|Neobisium_tantaleum|CROBD660|Neobisium','CROPS179-21|Neobisium_tantaleum|CROBD920|Neobisium','CROPS013-21|Neobisium_tantaleum|CROBD399|Neobisium','CROPS333-21|Neobisium_tantaleum|CROBD654|Neobisium','CROPS182-21|Neobisium_tantaleum|CROBD923|Neobisium','CROPS457-22|Neobisium_tantaleum|CROBD918|Neobisium'

Group 96 (support = 1.000)

'CROPS360-21|Neobisium_reimoseri|CROBD754|Neobisium'

Group 97 (support = 1.000)

'CROPS250-21|Neobisium_reimoseri|CROBD294|Neobisium'

Group 98 (support = 0.884)

'CROPS338-21|Neobisium_reimoseri|CROBD659|Neobisium','CROPS195-21|Neobisium_reimoseri|CROBD217|Neobisium','CROPS337-21|Neobisium_reimoseri|CROBD658|Neobisium'

Group 99 (support = 0.962)

'CROPS346-21|Neobisium_reimoseri|CROBD667|Neobisium','CROPS343-21|Neobisium_reimoseri|CROBD664|Neobisium'

Group 100 (support = 1.000)

'CROPS147-21|Neobisium_aff._reimoseri|CROBD828|Neob'

Group 101 (support = 1.000)

'CROPS141-21|Neobisium_gentile_flavum|CROBD818|Neob'

Group 102 (support = 1.000)

'CROPS156-21|Neobisium_gentile_flavum|CROBD844|Neob'

Group 103 (support = 1.000)

'CROPS319-21|Neobisium_sp._nov._M|CROBD621|Neobisiu'

Group 104 (support = 0.962)

'CROPS353-21|Neobisium_sp._nov._M|CROBD700|Neobisiu','CROPS191-21|Neobisium_sp._nov._M|CROBD212|Neobisiu'

Group 105 (support = 0.909)

'CROPS321-21|Neobisium_aff._svetovidi|CROBD623|Neob','CROPS078-21|Neobisium_aff._svetovidi|CROBD597|Neob','CROPS051-21|Neobisium_aff._svetovidi|CROBD565|Neob','CROPS089-21|Neobisium_aff._svetovidi|CROBD610|Neob','CROPS271-21|Neobisium_aff._svetovidi|CROBD334|Neob'

Group 106 (support = 0.942)

'CROPS183-21|Neobisium_stribogi|CROBD201|Neobisium','CROPS192-21|Neobisium_stribogi|CROBD213|Neobisium','CROPS193-21|Neobisium_stribogi|CROBD215|Neobisium'

Group 107 (support = 0.660)
'CROPS278-21|Neobisium_aff._stygium|CROBD346|Neobis','CROPS318-21|Neobisium_aff._stygium|CROBD620|Neobis','CROPS335-21|Neobisium_aff._stygium|CROBD656|Neobis','CROPS462-22|Neobisium_aff._stygium|CROBD1138|Neobi'
Group 108 (support = 0.685)
'CROPS115-21|Chthonius_subterraneus_meuseli|CROBD69','CROPS109-21|Chthonius_subterraneus_meuseli|CROBD68','CROPS124-21|Chthonius_subterraneus_meuseli|CROBD74','CROPS122-21|Chthonius_subterraneus_meuseli|CROBD74','CROPS121-21|Chthonius_subterraneus_meuseli|CROBD74'
Group 109 (support = 0.998)
'CROPS476-22|Chthonius_subterraneus|CROBD1158|Chtho'
Group 110 (support = 1.000)
'CROPS367-21|Roncus_aff._ragusae|CROBD762|Roncus_af'
Group 111 (support = 1.000)
'CROPS004-21|Chthoniidae|CROBD386||Globochthonius_|'
Group 112 (support = 1.000)
'CROPS450-22|Chthoniidae|CROBD909||Globochthonius_|'
Group 113 (support = 1.000)
'CROPS010-21|Chthoniidae|CROBD396||Globochthonius_|'
Group 114 (support = 1.000)
'CROPS202-21|Chthonius_sp._1DH|CROBD225|Chthonius_s'
Group 115 (support = 0.494)
'CROPS411-22|Roncus_aff._ragusae|CROBD1117|Roncus_a','CROPS415-22|Roncus_aff._ragusae|CROBD1123|Roncus_a'
Group 116 (support = 1.000)
'CROPS021-21|Roncus_ragusae|CROBD410|Roncus_ragusae'
Group 117 (support = 0.833)
'CROPS452-22|Protoneobisium_biocovense|CROBD911|Pro','CROPS446-22|Protoneobisium_biocovense|CROBD899|Pro','CROPS454-22|Protoneobisium_biocovense|CROBD913|Pro','CROPS445-22|Protoneobisium_biocovense|CROBD898|Pro','CROPS009-21|Protoneobisium_biocovense|CROBD393|Pro','CROPS456-22|Protoneobisium_biocovense|CROBD917|Pro','CROPS008-21|Protoneobisium_biocovense|CROBD390|Pro','CROPS291-21|Protoneobisium_biocovense|CROBD370|Pro'
Group 118 (support = 0.969)
'CROPS418-22|Neobisium_staudacheri|CROBD407|Neobisi','CROPS005-21|Neobisium_staudacheri|CROBD387|Neobisi'
Group 119 (support = 0.714)
'CROPS225-21|Chthonius_aff._occultus|CROBD254|Chtho','CROPS224-21|Chthonius_aff._occultus|CROBD253|Chtho','CROPS223-21|Chthonius_aff._occultus|CROBD252|Chtho','CROPS221-21|Chthonius_aff._occultus|CROBD250|Chtho','CROPS220-21|Chthonius_aff._occultus|CROBD249|Chtho','CROPS218-21|Chthonius_aff._occultus|CROBD247|Chtho','CROPS334-21|Chthonius_aff._occultus|CROBD655|Chtho','CROPS014-21|Chthonius_aff._occultus|CROBD400|Chtho','CROPS340-21|Chthonius_aff._occultus|CROBD661|Chtho','CROPS341-21|Chthonius_aff._occultus|CROBD662|Chtho','CROPS012-21|Chthonius_aff._occultus|CROBD398|Chtho'
Group 120 (support = 0.343)
'CROPS447-22|Chthonius_aff._occultus|CROBD900|Chtho','CROPS002-21|Chthonius_aff._occultus|CROBD384|Chtho','CROPS449-22|Chthonius_aff._occultus|CROBD905|Chtho'
Group 121 (support = 0.810)
'CROPS068-21|Insulocreagris_sp._nov._PI|CROBD584|In','CROPS093-21|Insulocreagris_sp._nov._PI|CROBD614|In','CROPS092-21|Insulocreagris_sp._nov._PI|CROBD613|In','CROPS066-21|Insulocreagris_sp._nov._PI|CROBD582|In'
Group 122 (support = 0.999)
'CROPS431-22|Insulocreagris_sp._nov._PI|CROBD820|In'
Group 123 (support = 0.500)
'CROPS264-21|Roncus_anophthalmus|CROBD325|Roncus_an','CROPS273-21|Roncus_anophthalmus|CROBD337|Roncus_an'
Group 124 (support = 1.000)
'CROPS039-21|Roncus_anophthalmus|CROBD443|Roncus_an'
Group 125 (support = 0.327)
'CROPS443-22|Neobisium_gentile_flavum|CROBD891|Neob','CROPS444-22|Neobisium_gentile_flavum|CROBD893|Neob','CROPS148-21|Neobisium_gentile_flavum|CROBD829|Neob'
Group 126 (support = 1.000)
'CROPS172-21|Neobisium_gentile_flavum|CROBD888|Neob'
Group 127 (support = 1.000)
'CROPS483-22|Chthonius_magnificus|CROBD1165|Chthoni'
Group 128 (support = 0.336)
'CROPS366-21|Chthonius_magnificus|CROBD761|Chthoniu','CROPS368-21|Chthonius_magnificus|CROBD763|Chthoniu','CROPS471-22|Chthonius_magnificus|CROBD1152|Chthoni'
Group 129 (support = 0.858)

'CROPS023-21|Chthonius_magnificus|CROBD412|Chthoniu','CROPS496-22|Chthonius_magnificus|CROBD1182|Chthoni','CROPS503-22|Chthonius_magnificus|CROBD1190|Chthoni','CROPS416-22|Chthonius_magnificus|CROBD1124|Chthoni'

Group 130 (support = 0.978)

'CROPS463-22|Neobisium_stygium|CROBD1139|Neobisium'

Group 131 (support = 0.755)

'CROPS140-21|Neobisium_stygium|CROBD817|Neobisium_s','CROPS155-21|Neobisium_stygium|CROBD839|Neobisium_s','CROPS359-21|Neobisium_stygium|CROBD753|Neobisium_s','CROPS344-21|Neobisium_stygium|CROBD665|Neobisium_s','CROPS345-21|Neobisium_stygium|CROBD666|Neobisium_s'

Group 132 (support = 0.989)

'CROPS249-21|Neobisium_stygium|CROBD293|Neobisium_s'

Group 133 (support = 0.448)

'CROPS363-21|Neobisium_stygium|CROBD758|Neobisium_s','CROPS361-21|Neobisium_stygium|CROBD756|Neobisium_s','CROPS362-21|Neobisium_stygium|CROBD757|Neobisium_s','CROPS279-21|Neobisium_stygium|CROBD347|Neobisium_s','CROPS163-21|Neobisium_stygium|CROBD864|Neobisium_s','CROPS048-21|Neobisium_stygium|CROBD562|Neobisium_s','CROPS087-21|Neobisium_stygium|CROBD608|Neobisium_s','CROPS088-21|Neobisium_stygium|CROBD609|Neobisium_s','CROPS053-21|Neobisium_stygium|CROBD567|Neobisium_s','CROPS055-21|Neobisium_stygium|CROBD569|Neobisium_s','CROPS130-21|Neobisium_stygium|CROBD787|Neobisium_s','CROPS128-21|Neobisium_stygium|CROBD783|Neobisium_s','CROPS077-21|Neobisium_stygium|CROBD593|Neobisium_s','CROPS064-21|Neobisium_stygium|CROBD580|Neobisium_s','CROPS063-21|Neobisium_stygium|CROBD578|Neobisium_s','CROPS133-21|Neobisium_stygium|CROBD799|Neobisium_s','CROPS065-21|Neobisium_stygium|CROBD581|Neobisium_s','CROPS052-21|Neobisium_stygium|CROBD566|Neobisium_s','CROPS177-21|Neobisium_stygium|CROBD915|Neobisium_s','CROPS131-21|Neobisium_stygium|CROBD797|Neobisium_s','CROPS069-21|Neobisium_stygium|CROBD585|Neobisium_s','CROPS420-22|Neobisium_stygium|CROBD624|Neobisium_s','CROPS272-21|Neobisium_stygium|CROBD335|Neobisium_s','CROPS274-21|Neobisium_stygium|CROBD339|Neobisium_s','CROPS188-21|Neobisium_stygium|CROBD207|Neobisium_s','CROPS270-21|Neobisium_stygium|CROBD332|Neobisium_s','CROPS190-21|Neobisium_stygium|CROBD209|Neobisium_s'

Group 134 (support = 0.519)

'CROPS129-21|Neobisium_stygium|CROBD786|Neobisium_s','CROPS380-21|Neobisium_stygium|CROBD895|Neobisium_s'

Group 135 (support = 0.164)

'CROPS266-21|Neobisium_stygium|CROBD328|Neobisium_s','CROPS281-21|Neobisium_stygium|CROBD351|Neobisium_s','CROPS108-21|Neobisium_stygium|CROBD687|Neobisium_s','CROPS103-21|Neobisium_stygium|CROBD681|Neobisium_s','CROPS125-21|Neobisium_stygium|CROBD744|Neobisium_s'

Group 136 (support = 0.773)

'CROPS405-22|Neobisium_stygium|CROBD1108|Neobisium','CROPS404-22|Neobisium_stygium|CROBD1107|Neobisium'

Group 137 (support = 0.962)

'CROPS398-22|Neobisium_stygium|CROBD1101|Neobisium'

Group 138 (support = 0.449)

'CROPS448-22|Neobisium_aff._spelaeum|CROBD902|Neobi','CROPS254-21|Neobisium_aff._spelaeum|CROBD302|Neobi','CROPS253-21|Neobisium_aff._spelaeum|CROBD301|Neobi','CROPS149-21|Neobisium_aff._spelaeum|CROBD831|Neobi','CROPS142-21|Neobisium_aff._spelaeum|CROBD819|Neobi','CROPS171-21|Neobisium_aff._spelaeum|CROBD887|Neobi','CROPS159-21|Neobisium_aff._spelaeum|CROBD848|Neobi','CROPS158-21|Neobisium_aff._spelaeum|CROBD847|Neobi','CROPS309-21|Neobisium_stygium|CROBD529|Neobisium_s','CROPS310-21|Neobisium_stygium|CROBD530|Neobisium_s','CROPS308-21|Neobisium_stygium|CROBD528|Neobisium_s','CROPS475-22|Neobisium_stygium|CROBD1157|Neobisium','CROPS477-22|Neobisium_stygium|CROBD1159|Neobisium','CROPS267-21|Neobisium_stygium|CROBD329|Neobisium_s','CROPS277-

21|Neobisium_stygium|CROBD344|Neobisium_s','CROPS276-21|Neobisium_stygium|CROBD343|Neobisium_s','CROPS354-21|Neobisium_stygium|CROBD703|Neobisium_s','CROPS430-22|Neobisium_stygium|CROBD809|Neobisium_s','CROPS112-21|Neobisium_stygium|CROBD691|Neobisium_s','CROPS113-21|Neobisium_stygium|CROBD693|Neobisium_s','CROPS355-21|Neobisium_stygium|CROBD748|Neobisium_s','CROPS099-21|Neobisium_stygium|CROBD677|Neobisium_s','CROPS127-21|Neobisium_stygium|CROBD747|Neobisium_s','CROPS304-21|Neobisium_stygium|CROBD524|Neobisium_s','CROPS268-21|Neobisium_stygium|CROBD330|Neobisium_s','CROPS114-21|Neobisium_stygium|CROBD695|Neobisium_s','CROPS110-21|Neobisium_stygium|CROBD689|Neobisium_s','CROPS320-21|Neobisium_stygium|CROBD622|Neobisium_s','CROPS100-21|Neobisium_stygium|CROBD678|Neobisium_s','CROPS303-21|Neobisium_stygium|CROBD522|Neobisium_s','CROPS098-21|Neobisium_stygium|CROBD676|Neobisium_s','CROPS097-21|Neobisium_stygium|CROBD675|Neobisium_s','CROPS126-21|Neobisium_stygium|CROBD746|Neobisium_s','CROPS392-22|Neobisium_stygium|CROBD1021|Neobisium'

Group 139 (support = 0.890)

'CROPS136-21|Neobisium_stygium|CROBD810|Neobisium_s','CROPS143-21|Neobisium_stygium|CROBD823|Neobisium_s','CROPS314-21|Neobisium_stygium|CROBD534|Neobisium_s','CROPS311-21|Neobisium_stygium|CROBD531|Neobisium_s','CROPS315-21|Neobisium_stygium|CROBD535|Neobisium_s','CROPS313-21|Neobisium_stygium|CROBD533|Neobisium_s','CROPS312-21|Neobisium_stygium|CROBD532|Neobisium_s'

Group 140 (support = 0.510)

'CROPS187-21|Lasiochernes|CROBD205||sp._1DH','CROPS248-21|Lasiochernes|CROBD290||sp._1DH'

Group 141 (support = 0.884)

'CROPS390-22|Lasiochernes|CROBD1019||sp._2DH','CROPS365-21|Lasiochernes|CROBD760||sp._2DH'

Group 142 (support = 0.468)

'CROPS229-21|Chthoniidae|CROBD259||Globochthonius_|','CROPS135-21|Chthoniidae|CROBD807||Globochthonius_|','CROPS436-22|Chthoniidae|CROBD866||Globochthonius_|','CROPS433-22|Chthoniidae|CROBD841||Globochthonius_|'

Group 143 (support = 0.489)

'CROPS364-21|Chthoniidae|CROBD759||Globochthonius_|','CROPS391-22|Chthoniidae|CROBD1020||Globochthonius'

Group 144 (support = 0.995)

'CROPS001-21|Roncus_lubricus|CROBD382|Roncus_lubric'

Group 145 (support = 0.511)

'CROPS206-21|Roncus_lubricus|CROBD232|Roncus_lubric','CROPS184-21|Roncus_lubricus|CROBD202|Roncus_lubric'

Group 146 (support = 1.000)

'CROPS234-21|Neobisium_gentile_alternum|CROBD267|Ne'

Group 147 (support = 0.400)

'CROPS369-21|Chthonius_sp._nov._PLJ|CROBD764|Chthon','CROPS090-21|Chthonius_sp._nov._PLJ|CROBD611|Chthon','CROPS379-21|Chthonius_sp._nov._PLJ|CROBD778|Chthon','CROPS062-21|Chthonius_sp._nov._PLJ|CROBD577|Chthon'

Group 148 (support = 0.984)

'CROPS228-21|Chthonius_aff._absoloni|CROBD258|Chtho'

Group 149 (support = 0.999)

'CROPS007-21|Neobisium_gentile_alternum|CROBD389|Ne'

Group 150 (support = 0.988)

'CROPS011-21|Neobisium_gentile_alternum|CROBD397|Ne'

Group 151 (support = 0.208)

'CROPS440-22|Neobisium_gentile_alternum|CROBD878|Ne','CROPS434-22|Neobisium_gentile_alternum|CROBD845|Ne','CROPS373-21|Neobisium_gentile_alternum|CROBD770|Ne','CROPS427-22|Neobisium_gentile_alternum|CROBD779|Ne','CROPS015-21|Neobisium_gentile_alternum|CROBD401|Ne'

Group 152 (support = 0.334)

'CROPS017-21|Chthoniidae|CROBD403||Globochthonius_|','CROPS350-21|Chthoniidae|CROBD672||Globochthonius_|','CROPS351-21|Chthoniidae|CROBD673||Globochthonius_|'

Group 153 (support = 1.000)

'CROPS484-22|Chthoniidae|CROBD1166||Globochthonius'

Group 154 (support = 0.975)

'CROPS033-21|Chthonius_magnificus|CROBD432|Chthoniu'

Group 155 (support = 0.769)

'CROPS154-21|Chthonius_magnificus|CROBD838|Chthoniu','CROPS412-22|Chthonius_magnificus|CROBD1120|Chthoni','CROPS410-22|Chthonius_magnificus|CROBD1116|Chthoni'

Group 156 (support = 0.889)
'CROPS201-21|Chthonius_aff._occultus|CROBD224|Chtho','CROPS200-21|Chthonius_aff._occultus|CROBD223|Chtho','CROPS235-21|Chthonius_aff._occultus|CROBD268|Chtho','CROPS197-21|Chthonius_aff._occultus|CROBD220|Chtho','CROPS198-21|Chthonius_aff._occultus|CROBD221|Chtho','CROPS199-21|Chthonius_aff._occultus|CROBD222|Chtho'

Group 157 (support = 0.723)
'CROPS246-21|Chthonius_aff._occultus|CROBD284|Chtho','CROPS375-21|Chthonius_aff._occultus|CROBD772|Chtho','CROPS376-21|Chthonius_aff._occultus|CROBD773|Chtho','CROPS395-22|Chthonius_aff._occultus|CROBD1081|Chth'
Group 158 (support = 0.990)
'CROPS442-22|Chthoniidae|CROBD885||Ephippiochthoniu'
Group 159 (support = 0.587)
'CROPS282-21|Chthoniidae|CROBD353||Ephippiochthoniu','CROPS283-21|Chthoniidae|CROBD354||Ephippiochthoniu'
Group 160 (support = 0.998)
'CROPS382-22|Roncus_aff._lubricus|CROBD1005|Roncus'
Group 161 (support = 0.998)
'CROPS243-21|Roncus_aff._lubricus|CROBD277|Roncus_a'
Group 162 (support = 0.924)
'CROPS275-21|Neobisium_aff._spelaeum|CROBD342|Neobi','CROPS263-21|Neobisium_aff._spelaeum|CROBD323|Neobi','CROPS336-21|Neobisium_aff._spelaeum|CROBD657|Neobi'
Group 163 (support = 0.998)
'CROPS019-21|Neobisium_peruni|CROBD405|Neobisium_pe'
Group 164 (support = 0.998)
'CROPS006-21|Neobisium_peruni|CROBD388|Neobisium_pe'
Group 165 (support = 0.964)
'CROPS285-21|Chthonius_ischnocheles|CROBD358|Chthon'
Group 166 (support = 0.944)
'CROPS326-21|Chthonius_ischnocheles|CROBD638|Chthon','CROPS323-21|Chthonius_ischnocheles|CROBD635|Chthon','CROPS325-21|Chthonius_ischnocheles|CROBD637|Chthon'
Group 167 (support = 0.765)
'CROPS145-21|Neobisium_stygium|CROBD826|Neobisium_s','CROPS358-21|Neobisium_stygium|CROBD752|Neobisium_s'
Group 168 (support = 0.870)
'CROPS150-21|Neobisium_stygium|CROBD832|Neobisium_s','CROPS170-21|Neobisium_stygium|CROBD886|Neobisium_s'
Group 169 (support = 0.995)
'CROPS403-22|Roncus_italicus|CROBD1106|Roncus_itali'
Group 170 (support = 0.967)
'CROPS400-22|Roncus_italicus|CROBD1103|Roncus_itali','CROPS397-22|Roncus_italicus|CROBD1100|Roncus_itali'
Group 171 (support = 0.880)
'CROPS480-22|Chthonius_sp._nov._PL|CROBD1162|Chthon','CROPS479-22|Chthonius_sp._nov._PL|CROBD1161|Chthon'
Group 172 (support = 0.112)
'CROPS111-21|Chthonius_absoloni|CROBD690|Chthonius','CROPS118-21|Chthonius_absoloni|CROBD699|Chthonius','CROPS123-21|Chthonius_absoloni|CROBD742|Chthonius','CROPS424-22|Chthonius_absoloni|CROBD705|Chthonius','CROPS423-22|Chthonius_absoloni|CROBD694|Chthonius','CROPS394-22|Chthonius_absoloni|CROBD1023|Chthonius','CROPS116-21|Chthonius_absoloni|CROBD697|Chthonius','CROPS105-21|Chthonius_absoloni|CROBD683|Chthonius','CROPS106-21|Chthonius_absoloni|CROBD684|Chthonius','CROPS117-21|Chthonius_absoloni|CROBD698|Chthonius','CROPS107-21|Chthonius_absoloni|CROBD685|Chthonius','CROPS393-22|Chthonius_absoloni|CROBD1022|Chthonius','CROPS104-21|Chthonius_absoloni|CROBD682|Chthonius','CROPS425-22|Chthonius_absoloni|CROBD706|Chthonius','CROPS422-22|Chthonius_absoloni|CROBD686|Chthonius'
Group 173 (support = 0.676)
'CROPS469-22|Chthonius_raridentatus|CROBD1149|Chtho','CROPS399-22|Chthonius_raridentatus|CROBD1102|Chtho','CROPS401-22|Chthonius_raridentatus|CROBD1104|Chtho'
Group 174 (support = 0.882)
'CROPS474-22|Chthonius_sp._nov._K|CROBD1156|Chthoni','CROPS262-21|Chthonius_sp._nov._K|CROBD321|Chthoniu','CROPS307-21|Chthonius_sp._nov._K|CROBD527|Chthoniu'
Group 175 (support = 0.985)
'CROPS260-21|Chthonius_sp._nov._M2|CROBD315|Chthoni'
Group 176 (support = 0.985)
'CROPS258-21|Chthonius_sp._nov._M2|CROBD310|Chthoni'
Group 177 (support = 0.662)
'CROPS212-21|Chthonius_aff._alpicola|CROBD241|Chtho','CROPS214-21|Chthonius_aff._alpicola|CROBD243|Chtho'
Group 178 (support = 0.863)

'CROPS245-21|Chthonius_aff._alpicola|CROBD283|Chtho'
Group 179 (support = 0.910)
'CROPS294-21|Roncus_sp._1DH|CROBD376|Roncus_sp._1DH'
Group 180 (support = 0.849)
'CROPS297-21|Roncus_sp._1DH|CROBD379|Roncus_sp._1DH','CROPS298-21|Roncus_sp._1DH|CROBD380|Roncus_sp._1DH'
Group 181 (support = 0.936)
'CROPS481-22|Neobisium_sp._1DH|CROBD1163|Neobisium'
Group 182 (support = 0.875)
'CROPS134-21|Neobisium_aff._svetovidi|CROBD801|Neob','CROPS046-21|Neobisium_aff._svetovidi|CROBD560|Neob','CROPS057-21|Neobisium_aff._svetovidi|CROBD571|Neob','CROPS280-21|Neobisium_aff._svetovidi|CROBD349|Neob','CROPS083-21|Neobisium_aff._svetovidi|CROBD603|Neob','CROPS489-22|Neobisium_aff._svetovidi|CROBD1172|Neo','CROPS132-21|Neobisium_aff._svetovidi|CROBD798|Neob','CROPS269-21|Neobisium_aff._svetovidi|CROBD331|Neob','CROPS059-21|Neobisium_aff._svetovidi|CROBD574|Neob','CROPS085-21|Neobisium_aff._svetovidi|CROBD605|Neob'
Group 183 (support = 0.622)
'CROPS056-21|Chthonius_aff._absoloni|CROBD570|Chtho','CROPS054-21|Chthonius_aff._absoloni|CROBD568|Chtho','CROPS071-21|Chthonius_aff._absoloni|CROBD587|Chtho'
Group 184 (support = 0.822)
'CROPS084-21|Chthonius_sp._nov._P|CROBD604|Chthoniu'
Group 185 (support = 0.978)
'CROPS455-22|Neobisium_maderi|CROBD916|Neobisium_ma'
Group 186 (support = 0.752)
'CROPS178-21|Neobisium_maderi|CROBD919|Neobisium_ma','CROPS381-21|Neobisium_maderi|CROBD901|Neobisium_ma','CROPS453-22|Neobisium_maderi|CROBD912|Neobisium_ma'
Group 187 (support = 0.800)
'CROPS407-22|Chthonius_ischnocheles_ischnocheles|CR'
Group 188 (support = 0.800)
'CROPS409-22|Chthonius_ischnocheles_ischnocheles|CR'
Group 189 (support = 0.321)
'CROPS026-21|Chthonius_subterraneus_subterraneus|CR','CROPS025-21|Chthonius_subterraneus_subterraneus|CR'
Group 190 (support = 0.659)
'CROPS161-21|Chthonius_subterraneus_subterraneus|CR'
Group 191 (support = 0.939)
'CROPS166-21|Neobisium_gentile_gentile|CROBD871|Neo'
Group 192 (support = 0.779)
'CROPS167-21|Neobisium_gentile_gentile|CROBD873|Neo','CROPS486-22|Neobisium_gentile_gentile|CROBD1168|Ne','CROPS485-22|Neobisium_gentile_gentile|CROBD1167|Ne','CROPS146-21|Neobisium_gentile_gentile|CROBD827|Neo','CROPS437-22|Neobisium_gentile_gentile|CROBD872|Neo'
Group 193 (support = 0.402)
'CROPS226-21|Neobisium_aff._elegans|CROBD256|Neobis'
Group 194 (support = 0.402)
'CROPS330-21|Neobisium_aff._elegans|CROBD645|Neobis'
Group 195 (support = 0.876)
'CROPS347-21|Chthonius_sp._nov._KO|CROBD669|Chthoni'
Group 196 (support = 0.876)
'CROPS164-21|Chthonius_sp._nov._D|CROBD868|Chthoniu'
Group 197 (support = 0.680)
'CROPS383-22|Neobisium_oculatum|CROBD1011|Neobisium'
Group 198 (support = 0.610)
'CROPS491-22|Neobisium_aff._oculatum|CROBD1176|Neob','CROPS492-22|Neobisium_aff._oculatum|CROBD1177|Neob','CROPS490-22|Neobisium_aff._oculatum|CROBD1174|Neob'
Group 199 (support = 0.769)
'CROPS466-22|Neobisium_oculatum|CROBD1144|Neobisium'

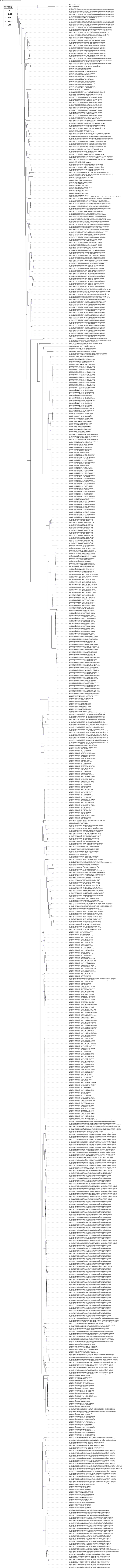
1
bootstrap
75
81.25
87.5
93.75
100
Platybunus pinetorum
Pandinus imperator
Euscorpius italicus
CROPS284-21 Chthoniidae CROBD355 Ephippiochthonius Ephippiochthonius tetrachelatus
CROPS289-21 Chthoniidae CROBD365 Ephippiochthonius Ephippiochthonius tetrachelatus
CROPS287-21 Chthoniidae CROBD362 Ephippiochthonius Ephippiochthonius tetrachelatus
CROPS286-21 Chthoniidae CROBD359 Ephippiochthonius Ephippiochthonius tetrachelatus
CROPS288-21 Chthoniidae CROBD364 Ephippiochthonius Ephippiochthonius tetrachelatus
Chthonius tetrachelatus beieri ZSM-BC-27399-D05 Baden-W
Chthonius tetrachelatus beieri GBOL10925 Baden-W
CROPS442-22 Chthoniidae CROBD885 Ephippiochthonius Ephippiochthonius tetrachelatus
CROPS282-21 Chthoniidae CROBD353 Ephippiochthonius Ephippiochthonius tetrachelatus
CROPS283-21 Chthoniidae CROBD354 Ephippiochthonius Ephippiochthonius tetrachelatus
CROPS372-21 Chthoniidae CROBD768 Ephippiochthonius Ephippiochthonius aff. insularis
CROPS252-21 Chthoniidae CROBD300 Ephippiochthonius Ephippiochthonius insularis
CROPS251-21 Chthoniidae CROBD298 Ephippiochthonius Ephippiochthonius insularis
CROPS499-22 Chthoniidae CROBD1186 Ephippiochthonius Ephippiochthonius insularis
CROPS010-21 Chthoniidae CROBD396 Globochthonius Globochthonius sp. n. B
CROPS450-22 Chthoniidae CROBD909 Globochthonius Globochthonius sp. nov. B
CROPS004-21 Chthoniidae CROBD386 Globochthonius Globochthonius sp. n. B
CROPS202-21 Chthonius sp. 1DH CROBD225 Chthonius sp. 1DH
CROPS429-22 Chthonius sp. nov. R CROBD806 Chthonius sp. nov. R
CROPS245-21 Chthonius aff. alpicola CROBD283 Chthonius aff. alpicola
CROPS212-21 Chthonius aff. alpicola CROBD241 Chthonius aff. alpicola
CROPS214-21 Chthonius aff. alpicola CROBD243 Chthonius aff. alpicola
CROPS096-21 Chthonius aff. absoloni CROBD618 Chthonius aff. absoloni
CROPS233-21 Chthonius aff. absoloni CROBD263 Chthonius aff. absoloni
CROPS232-21 Chthonius aff. absoloni CROBD262 Chthonius aff. absoloni
CROPS228-21 Chthonius aff. absoloni CROBD258 Chthonius aff. absoloni
CROPS369-21 Chthonius sp. nov. PLJ CROBD764 Chthonius sp. nov. PLJ
CROPS090-21 Chthonius sp. nov. PLJ CROBD611 Chthonius sp. nov. PLJ
CROPS379-21 Chthonius sp. nov. PLJ CROBD778 Chthonius sp. nov. PLJ
CROPS062-21 Chthonius sp. nov. PLJ CROBD577 Chthonius sp. nov. PLJ
CROPS029-21 Chthoniidae CROBD423 Ephippiochthonius Ephippiochthonius insularis
CROPS043-21 Chthoniidae CROBD557 Ephippiochthonius Ephippiochthonius insularis
CROPS408-22 Chthonius sp. ZB CROBD1113 Chthonius sp. ZB
CROPS042-21 Chthoniidae CROBD555 Ephippiochthonius Ephippiochthonius sp. 1DH
CROPS470-22 Chthonius sp. LI CROBD1151 Chthonius sp. LI
CROPS402-22 Chthonius sp. ZB CROBD1105 Chthonius sp. ZB
Chthonius tetrachelatus GBOL10929 Bavaria
Chthonius tetrachelatus GBOL19965 Bavaria
Chthonius tetrachelatus ZFMK-TIS-2528593 Meck-pomm
Chthonius tetrachelatus ZSM-BC-27570-H01 Bavaria
Chthonius tetrachelatus GBOL19967 Bavaria
Chthonius tetrachelatus ZFMK-TIS-2528594 Meck-pomm
Chthonius tetrachelatus GBOL18656 Bavaria
Chthonius tetrachelatus ZFMK-TIS-2501454 Hesse
Chthonius tetrachelatus ZFMK-TIS-2553342 Saxony
Chthonius tetrachelatus ZFMK-TIS-2576364 Thuringia
Chthonius tetrachelatus GBOL14967 Baden-W
Chthonius tetrachelatus ZFMK-TIS-2553341 Saxony
Chthonius tetrachelatus ZSM-BC-27399-F08 Baden-W
Chthonius alpicola GBOL10938 Bavaria
CROPS479-22 Chthonius sp. nov. PL CROBD1161 Chthonius sp. nov. PL
CROPS480-22 Chthonius sp. nov. PL CROBD1162 Chthonius sp. nov. PL
CROPS111-21 Chthonius absoloni CROBD690 Chthonius absoloni
CROPS118-21 Chthonius absoloni CROBD699 Chthonius absoloni
CROPS123-21 Chthonius absoloni CROBD742 Chthonius absoloni
CROPS422-22 Chthonius absoloni CROBD686 Chthonius absoloni
CROPS424-22 Chthonius absoloni CROBD705 Chthonius absoloni
CROPS423-22 Chthonius absoloni CROBD694 Chthonius absoloni
CROPS425-22 Chthonius absoloni CROBD706 Chthonius absoloni
CROPS394-22 Chthonius absoloni CROBD1023 Chthonius absoloni
CROPS106-21 Chthonius absoloni CROBD684 Chthonius absoloni
CROPS105-21 Chthonius absoloni CROBD683 Chthonius absoloni
CROPS116-21 Chthonius absoloni CROBD697 Chthonius absoloni
CROPS117-21 Chthonius absoloni CROBD698 Chthonius absoloni
CROPS393-22 Chthonius absoloni CROBD1022 Chthonius absoloni
CROPS107-21 Chthonius absoloni CROBD685 Chthonius absoloni
CROPS104-21 Chthonius absoloni CROBD682 Chthonius absoloni
CROPS102-21 Chthonius sp. LK CROBD680 Chthonius sp. LK
CROPS176-21 Chthoniidae CROBD907 Microchthonius Microchthonius elegantissimus
CROPS049-21 Chthonius sp. nov. V CROBD563 Chthonius sp. nov. V
CROPS258-21 Chthonius sp. nov. M2 CROBD310 Chthonius sp. nov. M2
CROPS260-21 Chthonius sp. nov. M2 CROBD315 Chthonius sp. nov. M2
CROPS385-22 Chthonius sp. nov. M1 CROBD1013 Chthonius sp. nov. M1
CROPS384-22 Chthonius sp. nov. M1 CROBD1012 Chthonius sp. nov. M1
Chthonius ischnocheles GBOL10919 Baden-W
Chthonius ischnocheles ZSM-BC-27399-F07 Baden-W
CROPS473-22 Chthonius aff. ischnocheles CROBD1155 Chthonius aff. ischnocheles
CROPS472-22 Chthonius aff. ischnocheles CROBD1154 Chthonius aff. ischnocheles
CROPS406-22 Chthonius ischnocheles reductus CROBD1109 Chthonius ischnocheles
CROPS407-22 Chthonius ischnocheles ischnocheles CROBD1110 Chthonius ischnocheles
CROPS409-22 Chthonius ischnocheles ischnocheles CROBD1114 Chthonius ischnocheles
CROPS285-21 Chthonius ischnocheles CROBD358 Chthonius ischnocheles
CROPS326-21 Chthonius ischnocheles CROBD638 Chthonius ischnocheles
CROPS325-21 Chthonius ischnocheles CROBD637 Chthonius ischnocheles
CROPS323-21 Chthonius ischnocheles CROBD635 Chthonius ischnocheles
CROPS435-22 Chthonius sp. nov. L CROBD865 Chthonius sp. nov. L
CROPS101-21 Chthonius sp. nov. L CROBD679 Chthonius sp. nov. L

Supplementary Figure 1: Representation of *COI* diversity at the spatial scale. The X-axis shows distances in km, and Y-axis shows uncorrected pairwise distances (*p*-distances) for the families Chthoniidae (A) and Neobisiidae (B).

Supplementary Figure 2: Phylogeographic structure of *Neobisium sylvaticum*.

A. ML phylogenetic tree. Terminal codes represent Sample IDs/BOLD IDs in BOLD. Numbers on nodes represent ultrafast bootstrap support values (BS). The tree was annotated in FigTree ver. 1.4.3 (Rambaut, 2009) and finished in Adobe Illustrator. B. MJ network of *COI* sequences. Colours indicate different sampling localities. Numbers of mutational steps are given as hatch marks. Black dots indicate extinct ancestral or unsampled haplotypes. Frequencies of haplotypes are proportional to the size of the circles. C. Sampling localities. D. Habitus of *Neobisium sylvaticum*. Abbreviations: CRO = Croatia, CG = Central Germany and SG = South Germany.

References

Hasegawa, M., Kishino, H., & Yano, T. Dating of the human-ape splitting by a molecular clock of mitochondrial DNA. *J. Mol. Evol.* **22**, 160–174 (1985).

Hebert, P. D. N., Cywinska, A., Ball, S. L., & deWaard, J. R. Biological identifications through DNA barcodes. *Proc. Royal Soc. B.* **270**, 313–321 (2003).

Hernández-Triana, L. M., Prosser, S. W., Rodríguez-Perez, M. A., Chaverri, L. G., Hebert, P. D. N., & Ryan Gregory, T. Recovery of DNA barcodes from blackfly museum specimens (Diptera: S imuliidae) using primer sets that target a variety of sequence lengths. *Mol. Ecol. Resour*. **14**, 508–518 (2014).

Folmer, O., Black, M., Hoeh, W., Lutz, R., Vrijenhoek, R. DNA primers for amplification of mitochondrial cytochrome c oxidase subunit I from diverse metazoan invertebrates. *Mol. Mar. Biol. Biotechnol*. **3**, 294–299 (1994).

Murienne, J., Harvey, M. S., & Giribet, G. First molecular phylogeny of the major clades of Pseudoscorpiones (Arthropoda: Chelicerata). *Mol. Phylogenet. Evol*. **49**, 170–184 (2008).

Tavaré, S. Some probabilistic and statistical problems in the analysis of DNA sequences. *Lect. Math. Life. Sci.* **17**, 57–86 (1986).

Tamura, K., & Nei, M. Estimation of the number of nucleotide substitutions in the control region of mitochondrial DNA in humans and chimpanzees. *Mol. Biol. Evol*. **10**, 512–526 (1993).
